# Supplementary material for: Novel 2-Amino-1,4-Naphthoquinone Derivatives Induce A549 Cell Death through Autophagy
Source: Molecules. 2023 Apr 7;28(8):3289. doi: 10.3390/molecules28083289 (PMC10143525; doi:10.3390/molecules28083289)
Supplement: Supplementary file 1 [file molecules-28-03289-s001.zip › molecules-2234868-supplementary.pdf]

Article

Novel 2-Amino-1,4-Naphthoquinone Derivatives Induce A549 Cell Death through Autophagy

Hua-Yuan Tan [1,†], Feng-Ming Liang [1,†], Wen-Jing Zhang [1], Yi Zhang [1], Jun-Hao Cui [1], Yu-Yu Dai [1], Xue-Mei Qiu [1], Wen-Hang Wang [1], Yue Zhou [1,2], Dan-Ping Chen [1,2,*] and Cheng-Peng Li [1,2,*]

1 School of Pharmaceutical Sciences, Guizhou University, Guiyang 550025, China; 13436002029@163.com (F.-M.L.)
2 Guizhou Engineering Laboratory for Synthetic Drugs, Guizhou University, Guiyang 550025, China
* Correspondence: dpchen@gzu.edu.cn (D.-P.C.); lichp11@163.com (C.-P.L.); Tel./Fax: +86-851-8830-8717 (D.-P.C.)
† These authors contributed equally to this work.

Content

1. Synthesis of intermediate 3a 3

2. Synthesis of target compounds 5a-5u 3

3. Synthesis of intermediate 8 4

4. Synthesis of target compounds 9a-9g 4

5. Analysis of anticancer activity of target compounds 5a-9g *in vitro* 5

6. The data of title compounds. 7

7. ^{1}H NMR, ^{13}C NMR, and HRMS spectra for target compounds. 16

8. Crystal raw data 59

9. Reference 60

1. Synthesis of intermediate 3a.

Taking intermediate **3a** as an example, the synthesis method of intermediate **3a** refers to the literature source [1], and 1.0g o-toluidine and 1.29g potassium carbonate are dissolved in 27mL DCM (Dichloromethane). The reaction mixture was stirred in the ice bath for about 10 minutes, and 2.07g of bromoacetyl bromide was added to make the reaction mixture react to room temperature. TLC tracking reaction ends. Then, spin dry the solvent, wash and filter with water, and dry to obtain 2-bromo-N- (2-benzyl) acetamide **3a**.

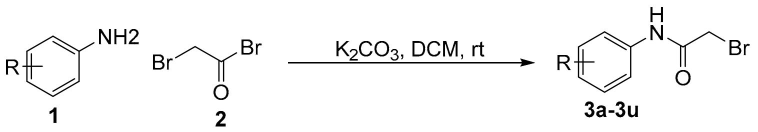

Fig S1. Synthesis of intermediate 3a-3u

2. Synthesis of target compounds 5a-5u

Take compound **5a** as an example, add 1g of 2-hydroxy-1,4 naphthoquinone in a 25mL round bottom flask, then add 1.87g of cesium carbonate to dissolve in 15mL of DMF, stir at room temperature (25 ℃), react for 1h, add 1.31g of the first step product, compound **3a**, and stir overnight. Stir at room temperature (25 ℃). After another 12-24 hours of reaction, TLC tracks the reaction to the end. Then prepare a 500mL beaker, add saturated salt ice water, and stir for about 10 minutes. Filter the red sediment in the beaker, wash it with water, dry it, select a suitable solvent for recrystallization, and purify it with thin chromatography to obtain a pure product **5a**.

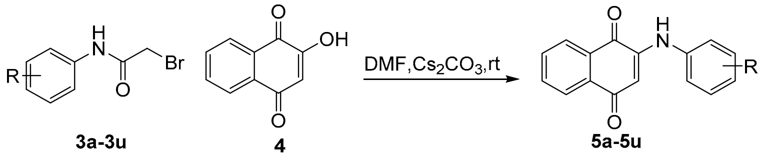

Fig S2. Synthesis of target compounds 5a-5u

3. Synthesis of intermediate 8

The synthesis method of intermediate **8** refers to the literature source [2]. First, in a 100mL round bottom flask, add 1,4-naphthoquinone (0.5g, 6.32mmol) and Cu (OAC)$_2$ (0.054g, 0.63mmol) to 7mL of AcOH solvent, stir slowly for 10min, then heat gently to dissolve it (the reaction temperature is mainly controlled at 65℃-70℃), slowly add 3-amino-4-methylbenzoic acid 7 (0.48g, 6.32mmol), and TLC tracks the reaction to the end. Then add the mixture into the beaker filled with saturated salt ice water, drop 10% NaOH (aq) into the beaker to make it alkaline, the suspension changes from reddish brown to blue black, filter to obtain the filtrate, adjust the filtrate to strong acidity, that is, the PH value is about 2-3, and a large amount of red precipitate rapidly appears in the filtrate. Filter, wash and precipitate, and dry to obtain the intermediate **8**.

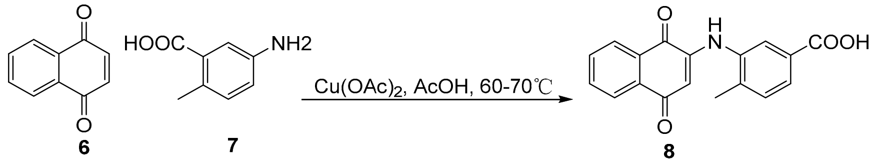

Fig S3. Synthesis of intermediate 8

4. Synthesis of target compounds 9a-9g

Taking compound **9f** as an example, the intermediate **8** (0.3g, 6.63mmol) was dissolved in 5mL of DCM solvent, stirred at room temperature (25℃), reacted for 1h, and then N-ethyldiisopropylamine (DIPEA, 0.4g, 4.88mmol) was added dropwise, followed by HATU (0.6g, 2.44mmol). At room temperature (25℃), stir for 1h, add 3-fluoroaniline (0.14g, 1.10mmol), continue to stir at room temperature, and then react for 12-24h. After the reaction is detected by TLC, the solvent is removed by rotary evaporator, the inorganic salt is removed by washing with saturated salt water (if the product is oily, it can be solidified with methanol), filtered, dried, recrystallized by ethyl acetate, and purified by column chromatography to obtain the target compound **9f**.

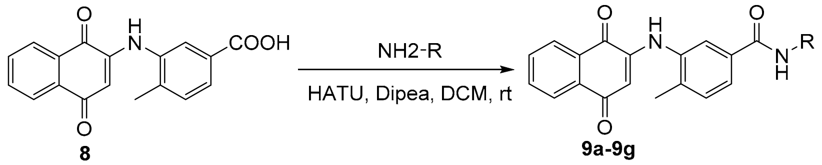

8 **9a-9g**

Fig S4. Synthesis of target compounds 9a-9g

5. Analysis of anticancer activity of target compounds 5a-9g *in vitro* .

Table S1. *In vitro* anticancer activity of target compounds 5a-5u (10 μM)

compd.	Inhibition rate [a] /%				
	R	**A549**	**PC-3**	**HepG2**	**K562**
**5a**	2-CH_3	19.59±6.83	26.71±5.54	20.60±8.01	19.96±2.13
**5b**	3-CH_3	13.30±2.47	18.3±3.32	5.52±5.65	7.71±4.37
**5c**	4-CH_3	13.16±2.91	26.21±2.82	15.82±4.95	9.54±3.41
**5d**	2-Cl	29.44±3.78	21.63±1.54	9.68±5.99	12.24±5.29
**5e**	3-Cl	28.32±8.75	31.04±1.50	10.11±3.96	21.32±2.65

**5f**	4-Cl	9.03±9.09	26.31±4.25	14.55±0.10	6.05±1.37
**5g**	2,4-diCl	23.45±7.16	25.64±1.49	18.54±3.81	9.20±4.15
**5h**	2-F	23.30±7.10	33.71±3.47	12.23±10.19	16.30±2.69
**5i**	3-F	34.80±3.58	44.65±4.84	38.14±3.56	24.26±3.93
**5j**	4-F	15.03±8.73	24.45±2.06	29.48±3.37	7.04±0.82
**5k**	2,4-diF	12.06±7.47	34.71±5.01	25.85±6.10	10.51±2.13
**5l**	2,6-diF	15.84±7.46	26.14±3.31	10.28±5.49	28.90±4.08
**5m**	3,5-diF	34.98±9.91	43.13±0.70	29.54±4.27	20.07±6.11
**5n**	2-Br	37.35±2.74	20.23±1.39	10.17±3.02	20.91±7.01
**5o**	3-Br	50.70±1.81	47.96±7.46	13.44±5.64	34.00±2.79
**5p**	4-Br	23.24±3.18	4.11±3.50	8.72±3.83	10.26±6.05
**5q**	2-OCH_3	34.03±5.13	18.74±5.78	19.40±5.78	18.77±4.54
**5r**	3-OCH_3	13.31±4.45	29.93±2.74	24.81±3.89	21.04±5.11
**5s**	4-OCH_3	19.69±2.70	17.33±2.24	15.67±4.17	17.92±2.02
**5t**	2-NO_2	14.36±3.50	23.42±2.33	8.76±2.39	5.91±2.45
**5u**	3-NO_2	14.76±4.49	27.47±3.10	11.88±2.14	5.75±2.58
	Gefitinib	29.48±4.24	21.88±6.58	30.41±2.37	30.51±3.42
	5-Fluorouracil	50.63±4.79	43.64±4.60	50.63±4.79	46.93±0.81

[a]: The average of three trials.

Table S2. *In vitro* anticancer activity of target compounds 9a-9g (10 μM)

compd.	Inhibition rate ^{a} /%				
	R	**A549**	**PC-3**	**HepG2**	**K562**
**9a**	Propylamine	37.47±1.52	28.10±4.84	17.26±5.09	26.93±1.38
**9b**	Butylamine	40.51±0.80	31.66±1.19	18.00±5.06	44.26±1.37
**9c**	phenyl	29.54±3.99	25.83±4.53	27.18±5.53	25.99±0.85
**9d**	Cyclopropyl	31.69±5.04	20.65±2.62	30.65±6.47	29.52±5.14
**9e**	2-fluorophenyl	27.11±1.41	6.37±6.83	23.20±3.66	40.41±3.01
**9f**	3-fluorophenyl	35.72±1.15	15.21±4.63	8.87±8.78	38.62±3.21
**9g**	3-bromophenyl	18.93±3.86	36.25±1.80	36.25±5.48	36.25±4.04

Gefitinib	29.48±4.24	35.05±6.58	30.41±2.37	30.51±3.42
5-Fluorouracil	54.24±3.69	43.64±4.60	41.26±3.26	46.93±0.81

[a]: The average of three trials.

6. The data of title compounds.

5a, yield of 62%, red solid, m.p 144-145 ℃; ^{1}H NMR (400 MHz, DMSO-*d6*) δ ppm: 9.06 (s, 1H), 8.07 (d, *J* = 7.5 Hz, 1H), 7.94 (dd, *J* = 7.6, 1.3 Hz, 1H), 7.86 (t, *J* = 7.4 Hz, 1H), 7.78 (t, *J* = 7.5 Hz, 1H), 7.31 (dddd, *J* = 13.7, 8.9, 7.3, 3.7 Hz, 4H), 5.32 (s, 1H), 2.21 (s, 3H). ^{13}C NMR (101 MHz, DMSO-*d6*) δ (ppm): 181.91, 148.23, 136.57, 135.33, 132.33, 131.131-129.44, 127.31, 126.09, 101.70, 17.88. HRMS (AP-ESI) m/z calcd for $C_{17}H_{13}NO_2$ [M+K]$^+$ 302.05779; found 302.05759.

5b, yield of 62%, red solid, m.p 157-158 ℃; ^{1}H NMR (500 MHz, DMSO-*d6*) δ (ppm): 9.16 (s, 1H), 8.04 − 7.99 (m, 1H), 7.90 (dd, *J* = 7.8, 1.0 Hz,1H), 7.81 (td, *J* = 7.5, 1.1 Hz, 1H), 7.74 (td, *J* = 7.5, 1.2 Hz,1H), 7.29 (dd, *J* = 14.0, 6.3 Hz,1H), 7.14 (d, *J* = 11.3 Hz, 2H), 7.00 (d, *J* = 7.6 Hz, 1H), 6.05 (s, 1H), 2.29 (s, 3H). ^{13}C NMR (126 MHz, DMSO-*d6*) δ (ppm): 183.08, 182.10, 146.73, 139.28, 138.46, 135.43, 133.12, 133.10, 130.91, 129.65, 126.61, 126.57, 125.79, 124.65, 121.37, 102.40, 21.53. HRMS (AP-ESI) m/z calcd for $C_{17}H_{13}NO_2$ [M+K]$^+$ 302.05779; found 302.05762.

5c, yield of 78%, red solid, m.p 177-178 ℃; ^{1}H NMR (500 MHz, DMSO-*d6*) δ (ppm):9.15 (s,1H), 8.00 (dd, *J* = 7.6, 1.1 Hz, 1H), 7.89 (dd, *J* = 7.6, 1.1 Hz, 1H), 7.81 (td, *J* = 7.5, 1.3 Hz, 1H), 7.73 (td, *J* = 7.5, 1.3 Hz, 1H), 7.20 (s, 4H), 5.97 (s, 1H), 2.26 (s, 3H). ^{13}C NMR (126 MHz, DMSO-*d6*) δ (ppm): 183.11, 147.01, 135.76, 135.50,

135.37, 133.18, 130.88, 130.32, 126.67, 125.81, 124.29, 101.93, 21.08. HRMS (AP-ESI) m/z calcd for $C_{17}H_{13}NO_2$ $[M+K]^+$ 302.05779: found 302.05692.

5d, yield of 78%, orange solid, m.p 165-166 ℃; 1H NMR (400 MHz, DMSO-*d6*) *δ* (ppm): 9.14 (s, 1H), 8.07 (dd, *J* = 7.6, 1.0 Hz, 1H), 7.94 (dd, *J* = 7.6, 1.1 Hz, 1H), 7.87 (td, *J* = 7.5, 1.4 Hz, 1H), 7.80 (td, *J* = 7.5, 1.5 Hz, 1H), 7.64 (d, *J* = 7.7 Hz, 1H), 7.48 (dd, *J* = 4.2, 1.2 Hz, 2H), 7.39 (ddd, *J* = 8.0, 5.6, 3.4 Hz, 1H), 5.44 (s, 1H). ^{13}C NMR (126 MHz, DMSO-*d6*) *δ* (ppm): 182.95, 181.72, 147.22, 135.59, 135.32, 133.37, 133.01, 130.86, 130.36, 128.97, 128.67, 126.70, 125.95, 103.40. HRMS (AP-ESI) m/z calcd for $C_{16}H_{10}ClNO_2$ $[M+H]^+$ 284.04728; found 284.04669.

5e, yield of 78%, red solid, m.p 197-198 ℃; ^1H NMR (400 MHz, DMSO-*d6*) *δ* (ppm):9.34 (s, 1H), 8.08 (dd, *J* = 7.6, 1.0 Hz, 1H), 8.00 – 7.96 (m, 1H), 7.88 (td, *J* = 7.5, 1.4 Hz, 1H), 7.81 (td, *J* = 7.5, 1.4Hz, 1H), 7.48 (dd, *J* = 4.9, 2.9 Hz, 2H), 7.40 (d, *J* = 8.1 Hz, 1H), 7.27 (ddd, *J* = 7.9, 2.0, 1.0 Hz, 1H), 6.17 (s, 1H). ^{13}C NMR (101 MHz, DMSO-*d6*) *δ* (ppm): 183.45, 146.30, 140.21, 135.48, 134.00, 133.36, 132.83, 131.44, 130.81, 126.69, 125.81, 125.40, 123.65, 122.41, 103.40. HRMS (AP-ESI) m/z calcd for $C_{16}H_{10}ClNO_2$ $[M+H]^+$ 284.04728; found 284.04767.

5f, yield of 46%, red solid, m.p 143-144 ℃; ^1H NMR (500 MHz, DMSO-*d6*) *δ* (ppm):9.29 (s, 1H), 8.03 (d, *J* = 6.8 Hz,1H), 7.91 (d, *J* = 7.3 Hz, 1H), 7.83 (dd, *J* = 7.4, 6.3 Hz, 1H), 7.76 (dd, *J* = 7.4, 6.3 Hz, 1H), 7.47 – 7.42 (m, 2H), 7.38 (q, *J* = 7.6 Hz, 2H), 6.11 (d, *J* = 18.8 Hz, 1H). ^{13}C NMR (126 MHz, DMSO-*d6*) *δ* (ppm): 182.83, 176.82, 158.35, 141.50, 139.11, 136.19, 135.45, 135.24, 134.99, 129.92, 125.79,

101.05, 100.00, 90.30. HRMS (AP-ESI) m/z calcd for $C_{16}H_{10}ClNO_2$ $[M+H]^+$ 284.04728; found 284.04617.

5g, yield of 61%, orange solid, m.p 175-176 ℃; ^{1}H NMR (400 MHz, DMSO-*d6*) *δ* (ppm): 9.11 (s, 1H), 8.07 (dd, *J* = 7.6, 1.0 Hz, 1H), 7.94 (dd, *J* = 7.6, 1.1 Hz, 1H), 7.90 − 7.85 (m, 2H), 7.83 − 7.78 (m, 2H), 7.54 (ddd, *J* = 18.0, 11.0, 4.7 Hz, 1H), 5.50 (d, *J* = 11.0 Hz, 1H). ^{13}C NMR (101 MHz, DMSO-*d6*) *δ* (ppm): 183.06, 181.53, 146.98, 135.58, 133.41, 130.70, 130.29, 129.80, 129.47, 129.03, 128.14, 126.66, 125.93, 103.74. HRMS (AP-ESI) m/z calcd for $C_{16}H_9Cl_2NO_2$ $[M+NH_3-H]^-$ 333.01921; found 333.02087.

5h, yield of 95%, orange solid, m.p 138-139 ℃; ^{1}H NMR (500 MHz, DMSO-*d6*) *δ* (ppm): 9.12 (s, 1H), 8.01 (dd, *J* = 7.5, 0.9 Hz,1H), 7.89 (dd, *J* = 7.5, 1.0 Hz, 1H), 7.81 (td, *J* = 7.5, 1.1 Hz, 1H), 7.74 (td, *J* = 7.5, 1.2 Hz, 1H), 7.41 (t, *J* = 7.8 Hz, 1H), 7.37 − 7.32 (m, 2H), 7.29 − 7.24 (m, 1H), 5.51 (d, *J* = 3.0 Hz, 1H). ^{13}C NMR (126 MHz, DMSO-*d6*) *δ* (ppm):182.95, 181.69, 157.66, 155.68, 147.31, 135.47, 133.29, 132.98, 130.85, 128.84, 128.35, 126.66, 125.90, 125.71, 125.72, 103.42. HRMS (AP-ESI) m/z calcd for $C_{16}H_{10}FNO_2$ $[M+Na]^+$ 290.05878; found 290.06018.

5i, yield of 78%, red solid, m.p 191-192 ℃; ^{1}H NMR (400 MHz, DMSO-*d6*) *δ* (ppm): 9.35 (s,1H), 8.07 (dd, *J* = 7.6, 1.2 Hz, 1H), 7.96 (dd, *J* = 7.6, 1.2 Hz, 1H), 7.88 (td, *J* = 7.5, 1.3 Hz, 1H), 7.80 (td, *J* = 7.5, 1.4 Hz, 1H), 7.53 − 7.43 (m, 1H), 7.31 − 7.24 (m, 2H), 7.10 − 7.00 (m, 1H), 6.22 (s, 1H). ^{13}C NMR (101 MHz, DMSO-*d6*) *δ* (ppm): 183.35, 181.88, 164.05, 146.09, 135.43, 133.30, 132.86, 131.46,

131.37, 130.86, 126.67, 125.79, 119.63, 112.02, 110.90, 110.66. HRMS (AP-ESI) m/z calcd for $C_{16}H_{10}FNO_2$ $[2M+K]^+$ 573.10227; found 573.10040.

5j, yield of 76%, red solid, m.p 242-243 ℃; ^{1}HNMR (500 MHz, DMSO-*d6*) δ (ppm):9.23 (s,1H), 8.02 (dd, *J* = 7.6, 1.1 Hz, 1H), 7.91 (dd, *J* = 7.6, 1.1 Hz, 1H), 7.82 (td, *J* = 7.5, 1.3 Hz, 1H), 7.75 (td, *J* = 7.5, 1.4 Hz, 1H), 7.41 – 7.35 (m, 2H), 7.27 – 7.22 (m, 2H), 5.96 (s, 1H). ^{13}C NMR (126 MHz, DMSO-*d6*) δ (ppm): 183.07, 182.05, 147.11, 135.45, 134.88, 134.14, 130.95, 126.90, 126.45, 125.82 116.71, 116.53, 102.24. HRMS (AP-ESI) m/z calcd for $C_{16}H_{10}FNO_2$ $[2M+Mg]^+$ 558.12231; found 558.12361.

5k, yield of 68%, orange solid, m.p 254-255 ℃; ^{1}HNMR (500 MHz, DMSO-*d6*) δ (ppm): 9.10 (s, 1H), 8.04 – 8.01 (m, 1H), 7.90 (dd, *J* = 7.7, 1.1 Hz,1H), 7.82 (td, *J* = 7.5, 1.2 Hz, 1H), 7.76 (td, *J* = 7.6, 1.3 Hz, 1H), 7.48 – 7.40 (m, 2H), 7.17 (ddd, *J* = 8.2, 2.9, 1.6 Hz, 1H), 5.45 (d, *J* = 2.7 Hz, 1H). ^{13}CNMR (126 MHz, DMSO-*d6*) δ (ppm): 182.95, 181.66, 155.98, 147.74, 135.50, 133.33, 132.97, 130.86, 126.66, 125.92, 112.91, 112.73, 105.94, 105.73, 105.54, 103.31. HRMS (AP-ESI) m/z calcd for $C_{16}H_9F_2NO_2$ $[M+H]^+$ 286.06741; found 286.06744.

5l, yield of 42%, orange solid, m.p 161-162 ℃; ^{1}HNMR (500 MHz, DMSO-*d6*) δ (ppm): 9.07 (s, 1H), 8.02 (dd, *J* = 7.5, 1.0 Hz,1H), 7.89 (dd, *J* = 7.5, 1.0 Hz, 1H), 7.82 (td, *J* = 7.5, 1.4 Hz, 1H), 7.76 (td, *J* = 7.5, 1.4 Hz, 1H), 7.44 (dq, *J* = 8.4, 6.4 Hz, 1H), 7.24 (dd, *J* = 13.7, 5.4 Hz, 2H), 5.31 (t, *J* = 1.7 Hz,1H). ^{13}C NMR (126 MHz, DMSO-*d6*) δ (ppm): 183.05, 181.42, 159.37, 157.38, 157.34, 147.68, 135.58, 133.49,

126.73, 125.99, 115.00, 114.87, 114.74, 113.23, 113.05, 103.83. HRMS (AP-ESI) m/z calcd for $C_{16}H_9F_2NO_2$ $[M+H]^+$ 286.06741; found 286.06732.

5m, yield of 50%, orange solid, m.p 258-259 ℃; ^{1}HNMR (500 MHz, DMSO-*d6*) δ (ppm): 9.36 (s, 1H), 8.08 – 8.01 (m, 1H), 7.93 (dd, *J* = 7.7, 1.1 Hz, 1H), 7.84 (td, *J* = 7.7, 1.4 Hz, 1H), 7.77 (td, *J* =7.5, 1.5 Hz, 1H), 7.17 – 7.11 (m, 2H), 7.01 (ddd, *J* = 9.4, 5.8, 2.3 Hz, 1H), 6.29 (s, 1H). ^{13}C NMR (126 MHz, DMSO-*d6*) δ (ppm): 183.62, 145.53, 135.51, 133.52, 130.89, 126.78, 125.89, 106.41, 105.24. HRMS (AP-ESI) m/z calcd for $C_{16}H_9F_2NO_2$ $[M+H]^+$ 286.06741; found 286.06741.

5n, yield of 67%, orange solid, m.p 134-135 ℃; ^{1}H NMR (400 MHz, DMSO-*d6*) δ (ppm): 9.07 (s, 1H), 8.07 – 7.73 (m, 5H), 7.39 (d, *J* = 74.7 Hz, 3H), 5.46 (s, 1H). ^{13}C NMR (101 MHz, DMSO-*d6*) δ (ppm): 182.82, 181.65, 147.13, 136.75, 135.52, 133.94, 133.27, 132.98, 130.74, 129.50, 129.19, 128.69, 126.61, 125.89, 121.02, 103.33. HRMS (AP-ESI) m/z calcd for $C_{16}H_{10}BrNO_2$ $[2M-H]^-$ 652.97061; found 652.97205.

5o, yield of 63%, red solid, m.p 197-198 ℃; ^{1}H NMR (400 MHz, DMSO-*d6*) δ (ppm): 9.32 (s, 1H), 8.07 (t, *J* = 7.2 Hz, 1H), 7.96 (t, *J* = 7.1 Hz, 1H), 7.89 – 7.79 (m, 2H), 7.60 (s, 1H), 7.44 – 7.39 (m, 3H), 6.16 (d, *J* = 8.0 Hz, 1H). ^{13}C NMR (126 MHz, DMSO-*d6*) δ (ppm): 146.28, 140.51, 135.47, 133.34, 131.70, 131.44, 128.24, 126.72, 126.55, 125.84, 122.80, 122.40, 119.12, 111.40, 103.54, 47.87. HRMS (AP-ESI) m/z calcd for $C_{16}H_{10}BrNO_2$ $[M+Na]^+$ 349.97871; found 349.98016.

5p, yield of 66%, red solid, m.p 266-267 ℃; ^{1}H NMR (500 MHz, DMSO-*d6*) δ (ppm): 9.28 (s, 1H), 8.02 (dt, *J* = 2.7, 1.4 Hz, 1H), 7.91 (dt, *J* = 2.6, 1.2 Hz, 1H), 7.85

-7.81 (m, 1H), 7.75 (ddd, *J* =7.2, 4.9, 1.1 Hz, 1H), 7.59 – 7.56 (m, 2H), 7.35 – 7.31 (m, 2H), 6.12 – 6.10 (m, 1H). ^{13}C NMR (101 MHz, DMSO-*d6*) *δ* (ppm):169.71, 166.78, 153.41, 148.03, 138.52, 138.21, 132.14, 132.09, 132.02, 131.87, 126.01, 121.69, 115.46, 114.70, 107.58, 47.40. HRMS (AP-ESI) m/z calcd for $C_{16}H_{10}BrNO_2$ $[2M-H]^-$ 652.97061; found 652.97101.

5q, yield of 81%， red soild , m.p 135-136 ℃; ^{1}H NMR (500 MHz, DMSO-*d6*) *δ* (ppm): 8.64 (s, 1H), 8.01 (dd, *J* = 7.5, 0.9 Hz, 1H), 7.90 (dd, *J* = 7.8, 1.0 Hz, 1H), 7.82 (td, *J* = 7.5, 1.2 Hz, 1H), 7.74 (td, *J* = 7.5, 1.2 Hz, 1H), 7.32 (dd, *J* = 7.9, 1.5 Hz, 1H), 7.26 – 7.21 (m, 1H), 7.13 (dd, *J* = 8.4, 1.0 Hz, 1H), 7.00 (td, *J* = 7.7, 1.2 Hz, 1H), 5.73 (s, 1H), 3.32 (s, 3H). ^{13}C NMR (101 MHz, DMSO-*d6*) *δ* (ppm): 182.81, 181.98, 152.80, 145.98, 135.48, 133.13, 130.72, 127.42, 126.62, 126.53, 125.83, 124.76, 121.32, 112.59, 102.97, 56.21. HRMS (AP-ESI) m/z calcd for $C_{17}H_{13}NO_3$ $[M+K]^+$ 318.05270; found 318.05289.

5r, yield of 78%, red soild, m.p 155-156 ℃; ^{1}H NMR (500 MHz, DMSO-*d6*) *δ* (ppm): 9.16 (s, 1H), 8.01 (dd, *J* = 7.6, 1.1 Hz, 1H), 7.90 (dd, *J* = 7.6, 1.1 Hz, 1H), 7.83 – 7.79 (m, 1H), 7.73 (td, *J* = 7.5, 1.2 Hz, 1H), 7.30 (t, *J* = 8.1 Hz, 1H), 6.93 (dt, J = 4.7, 1.9 Hz, 2H), 6.77 – 6.74 (m,1H), 6.11 (s, 1H), 3.34 (s, 3H). ^{13}C NMR (126 MHz, DMSO-*d6*) *δ* (ppm): 183.15, 182.05, 160.41, 146.49, 139.80, 135.42, 133.18, 133.04, 130.91, 130.60, 126.66, 125.79, 116.03, 111.23, 109.95, 103.00, 55.73. HRMS (AP-ESI) m/z calcd for $C_{17}H_{13}NO_3$ $[2M+Na]^+$ 581.16831; found 581.16901.

5s, yield of 85%, red soild, m.p 134-135 ℃; ^{1}H NMR (400 MHz, DMSO-*d6*) *δ* (ppm): 9.19 (s, 1H), 8.05 (dd, *J* = 7.6, 1.0 Hz, 1H), 7.94 (dd, *J* = 7.6, 1.0 Hz, 1H),

7.85 (td, *J* = 7.5, 1.4 Hz, 1H), 7.77 (td, *J* = 7.5, 1.4 Hz, 1H),7.32 – 7.25 (m, 2H) 7.04 – 6.99 (m, 2H), 5.92(s, 1H), 3.37 (s, 3H). ^{13}C NMR (101 Hz, DMSO-*d6*) *δ* (ppm): 182.70, 182.14, 157.38, 147.36, 135.36, 133.21, 132.96, 131.05, 130.90, 126.54, 126.08, 125.72, 121.18, 114.98, 101.46, 55.78. HRMS (AP-ESI) m/z calcd for $C_{17}H_{13}NO_3$ [M+Na]$^+$ 302.07876; found 302.07928.

5t, yield of 51%, orange soild, m.p 198-199 ℃;^{1}H NMR (400 MHz, DMSO-*d6*) *δ* (ppm): 9.76 (s, 1H), 8.21 (dd, *J* = 8.3, 1.3 Hz, 1H), 8.12 (dd, *J* = 7.6, 1.1 Hz, 1H), 7.99 (dd, *J* = 7.6, 1.1 Hz, 1H), 7.91 (td, *J* = 7.5, 1.4 Hz, 1H), 7.87 – 7.78 (m, 3H), 7.45 (ddd, *J* = 8.5, 7.0, 1.7 Hz, 1H), 6.26 (s, 1H). ^{13}C NMR (101 MHz, DMSO-*d6*) *δ* (ppm): 183.65, 146.63, 145.22, 141.90, 136.27, 135.85, 135.70, 133.69, 132.65, 126.88, 126.76, 126.00, 125.85, 125.58, 119.61, 116.04, 105.47. HRMS (AP-ESI) m/z calcd for $C_{16}H_{10}O_4N_2$ [M+H]$^+$ 295.07133; found 295.07104.

5u, yield of 52%, orange soild, m.p 277-278 ℃; ^{1}H NMR (500 MHz, DMSO-*d6*) *δ* (ppm): 9.52 (s, 1H), 8.20 (t, *J* = 2.1 Hz, 1H), 8.05 (dd, *J* = 7.6, 1.1 Hz, 1H), 8.00 – 7.97 (m, 1H), 7.93 (dd, *J* = 7.7, 1.1 Hz, 1H), 7.84 (ddd, *J* = 7.8, 5.5, 1.7 Hz, 2H), 7.78 (td, *J* = 7.5, 1.4 Hz, 1H), 7.67 (t, *J* = 8.3 Hz, 1H), 6.26 (s, 1H). ^{13}C NMR (126 MHz, DMSO-*d6*) *δ* (ppm): 186.30, 184.96, 181.07, 166.40, 162.78, 158.73, 153.54, 150.56, 135.34, 134.02, 119.24, 105.46, 100.00, 94.15, 87.17, 75.73. HRMS (AP-ESI) m/z calcd for $C_{16}H_{10}O_4N_2$ [M+H]$^+$ 295.07133; found 295.07104.

9a, yield of 50.2%, orange soild, m.p 175-176 ℃; ^{1}H NMR (500 MHz, DMSO-*d6*) *δ* (ppm): 9.07 (s, 1H), 8.45 (t, *J* = 5.6 Hz, 1H), 8.03 (dd, = 7.6, 1.2 Hz, 1H), 7.89 (dd, *J* = 7.6, 1.2 Hz, 1H), 7.81 (ddd, *J* = 7.5, 5.0, 2.6 Hz,1H), 7.76 – 7.69 (m, 3H), 7.41

(d, *J* = 8.1 Hz, 1H), 5.27 (s, 1H), 3.19 – 3.15 (m, 2H), 2.20 (s, 3H), 1.52 – 1.44 (m, 2H), 0.84 (dd, *J* = 9.3, 5.6 Hz, 3H). ^{13}C NMR (126 MHz, DMSO-*d6*) *δ* 182.67, 181.89, 165.66, 148.31, 138.55, 136.69, 135.45, 133.99, 133.28, 133.11, 131.50, 130.98, 126.60, 126.32, 125.88, 102.04, 41.54, 22.89, 17.94, 12.01. HRMS (AP-ESI) m/z calcd for $C_{21}H_{20}N_2O_3$ $[M+H]^+$ 349.15467; found 349.15448.

9b, yield of 57.13%, orange soild, m.p 136-138 ℃; ^{1}H NMR (500 MHz, DMSO-*d6*) *δ* (ppm): 9.06 (s, 1H), 8.43 (t, *J* = 5.4 Hz, 1H), 8.01 (d, *J* = 7.6 Hz, 1H), 7.88 (d, *J* = 7.6 Hz, 1H), 7.79 (t, *J* = 7.5 Hz, 1H), 7.74 – 7.71 (m, 3H), 7.39 (t, *J* = 7.3 Hz, 1H), 5.28 (s, 1H), 3.21 (dd, *J* = 12.9, 6.7 Hz, 2H), 2.20 (s, 3H), 1.47 – 1.43 (m, 2H), 1.29-1.25 (m, 2H), 0.84 (t, *J* = 6.9 Hz, 3H). ^{13}C NMR (126 MHz, DMSO-*d6*) *δ* (ppm): 182.65, 181.88, 165.62, 148.27, 138.51, 136.69, 135.39, 134.00, 133.29, 133.16, 131.47, 130.95, 126.56, 126.31, 125.86, 102.06, 31.74, 20.20, 17.93, 14.23. HRMS (AP-ESI) m/z calcd for $C_{22}H_{22}N_2O_3$ $[M+H]^+$ 363.17032; found 363.16971.

9c, yield of 62.93%, brown soild, m.p 176-177 ℃; ^{1}H NMR (500 MHz, DMSO-*d6*) *δ* (ppm): 9.05 (s, 1H), 8.03 (d, J = 7.5 Hz, 2H), 7.89 (d, *J* = 7.5 Hz, 2H), 7.84 – 7.80 (m, 2H), 7.80 – 7.77 (m, 2H), 7.75 (dd, *J* = 10.2, 3.7 Hz, 3H), 7.45 (d, *J* = 8.0 Hz, 2H), 5.27 (s, 1H), 2.22 (s, 3H). ^{13}C NMR (126 MHz, DMSO-*d6*) *δ* (ppm): 182.79, 181.80, 167.22, 148.32, 140.73, 136.96, 135.42, 133.15, 131.99, 131.02, 130.18, 128.41, 128.17, 126.61, 125.87, 102.02, 18.16. HRMS (AP-ESI) m/z calcd for $C_{24}H_{18}N_2O_3$ $[M+H]^+$ 383.13902; found 383.13840.

9d, yield of 60.64%, brown soild, m.p 216-217 ℃; ^{1}H NMR (500 MHz, DMSO-*d6*) *δ* (ppm): 9.08 (s, 1H), 8.44 (d, *J* = 4.2 Hz, 1H), 8.04 – 8.01 (m, 1H), 7.90 –

7.88 (m, 1H), 7.83 – 7.79 (m, 1H), 7.76 – 7.67 (m, 3H), 7.40 (d, *J* = 8.1 Hz, 1H), 5.25 (s, 1H), 2.82 – 2.78 (m, 1H), 2.19 (s, 3H), 0.66 – 0.62 (m, 2H), 0.54 – 0.51 (m, 2H). ^{13}C NMR (126 MHz, DMSO-*d6*) δ (ppm): 182.67, 181.89, 166.99, 148.35, 138.73, 136.67, 135.46, 133.64, 133.19, 131.48, 130.97, 126.60, 126.37, 125.88, 102.01, 23.60, 17.95, 6.24. HRMS (AP-ESI) m/z calcd for $C_{21}H_{18}N_2O_3$ $[M+H]^+$ 347.13902; found 347.13889.

9e, yield of 43.24%, yellow soild, m.p 162-163 ℃; ^1H NMR (400 MHz, DMSO-*d6*) δ (ppm): 10.16 (s, 1H), 9.16 (s, 1H), 8.09 (dd, *J* = 7.6, 1.0 Hz, 1H), 7.93 (ddd, *J* = 9.7, 7.8, 1.4 Hz, 2H), 7.89 – 7.85 (m, 3H), 7.80 (td, *J* = 7.5, 1.4 Hz, 1H), 7.60 (t, *J* = 7.1 Hz, 1H), 7.54 (d, *J* = 7.9 Hz, 1H), 7.29 (ddd, *J* = 6.0, 3.4, 1.6 Hz, 2H), 5.37 (s, 1H), 2.29 (s, 3H). ^{13}C NMR (101 MHz, DMSO-*d6*) δ (ppm): 182.68, 181.84, 164.92, 148.26, 139.54, 136.88, 135.41, 133.12, 131.69, 130.96, 127.77, 126.85, 126.57, 125.84, 124.76, 116.40, 102.15, 18.01. HRMS (AP-ESI) m/z calcd for $C_{24}H_{17}FN_2O_3$ $[M+H]^+$ 401.12960; found 401.12958.

9f, yield of 51.66%, red soild, m.p 173.0-174.0 ℃; ^1H NMR (400 MHz, DMSO-*d6*) δ (ppm): 10.44 (s, 1H), 9.19 (s, 1H), 8.09 (dd, *J* = 7.6, 0.9 Hz, 1H), 7.96 – 7.85 (m, 4H), 7.82 – 7.74 (m, 2H), 7.56 (dd, *J* = 10.8, 4.4 Hz, 2H), 7.38 (dt, *J* = 14.2, 7.1 Hz, 1H), 6.94 (td, *J* = 8.3, 2.3 Hz, 1H), 5.37 (s, 1H), 2.29 (s, 3H). ^{13}C NMR (101 MHz, DMSO-*d6*) δ (ppm): 182.67, 181.84, 165.19, 163.69, 161.30, 148.27, 141.29, 139.60, 136.88, 135.42, 133.69, 133.15, 131.66, 131.13, 130.38, 126.84, 126.57, 125.84, 116.53, 110.65, 107.68, 107.42, 102.12, 18.03. HRMS (AP-ESI) m/z calcd for $C_{24}H_{17}FN_2O_3$ $[M+H]^+$ 401.12960; found 401.12878.

9g, yield of 51.66%, fuchsia soild, m.p 238-239 ℃; ^{1}H NMR (500 MHz, DMSO-*d6*) *δ* (ppm): 10.34 (s, 1H), 9.14 (s, 1H), 8.05 (s, 2H), 7.90 (dd, *J* = 5.0, 2.7 Hz, 1H), 7.91 – 7.89 (m, 2H), 7.82 (dd, *J* =4.5 Hz, 2.8 Hz, 1H), 7.76 (d, *J* = 6.1 Hz, 1H), 7.74 – 7.71 (m, 1H), 7.51 (s, 1H), 7.30 – 7.24 (m, 2H), 5.32 (s, 1H), 2.24 (s, 3H). ^{13}C NMR (126 MHz, DMSO-*d6*) *δ* (ppm): 182.73, 181.89, 165.21, 148.33, 141.19, 139.70, 136.94, 135.48, 133.66, 133.21, 131.74, 131.18, 131.00, 126.90, 126.63, 125.91, 123.20, 121.92, 119.63, 118.27, 116.35, 102.17, 18.09. HRMS (AP-ESI) m/z calcd for $C_{24}H_{17}BrN_2O_3$ [M+H]$^+$ 461.04953; found 461.04977.

7.^{1}H NMR, ^{13}C NMR, and HRMS spectra for target compounds.

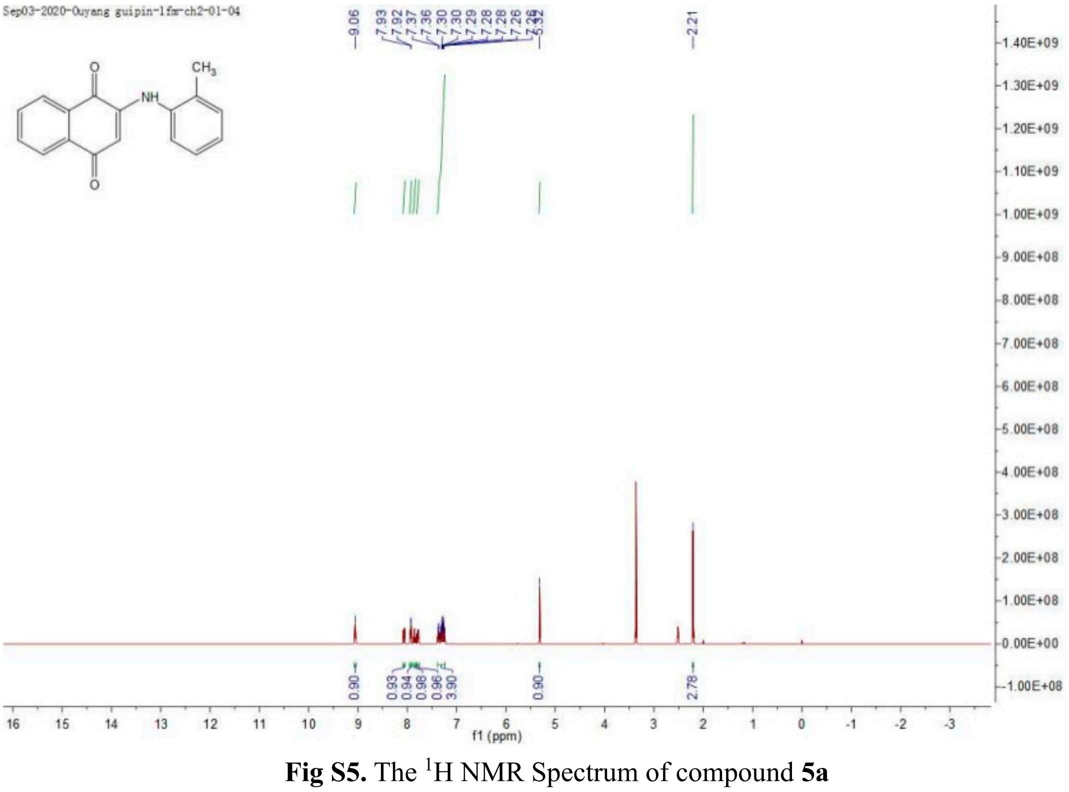

Fig S5. The ^{1}H NMR Spectrum of compound **5a**

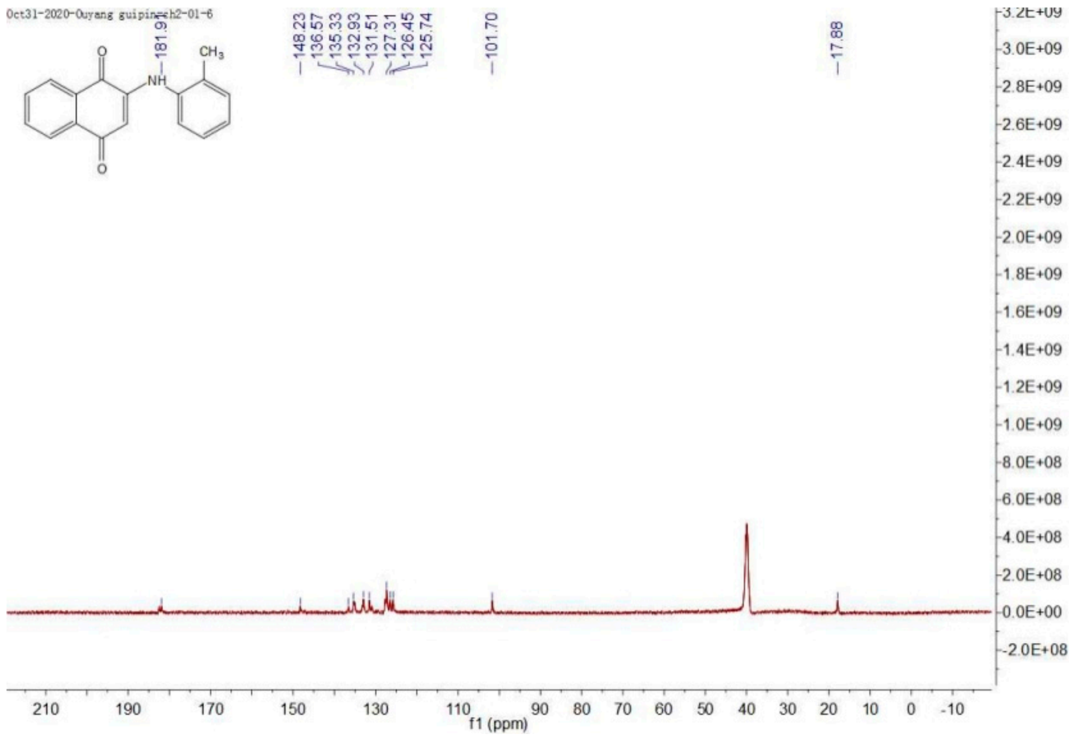

Fig S6. The ^{13}C NMR Spectrum of compound **5a**

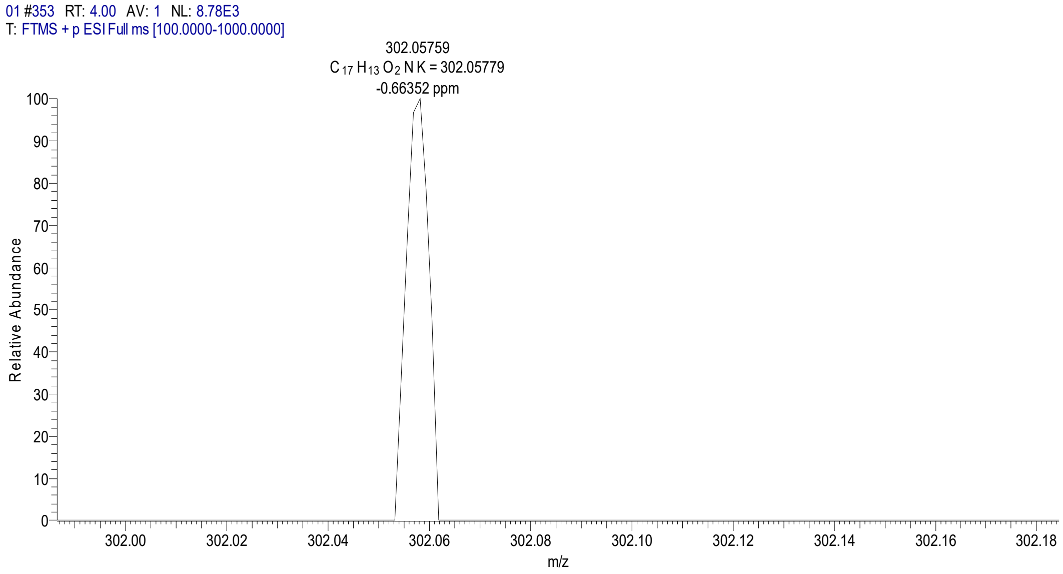

Fig S7. The HRMS spectrum of compound **5a**

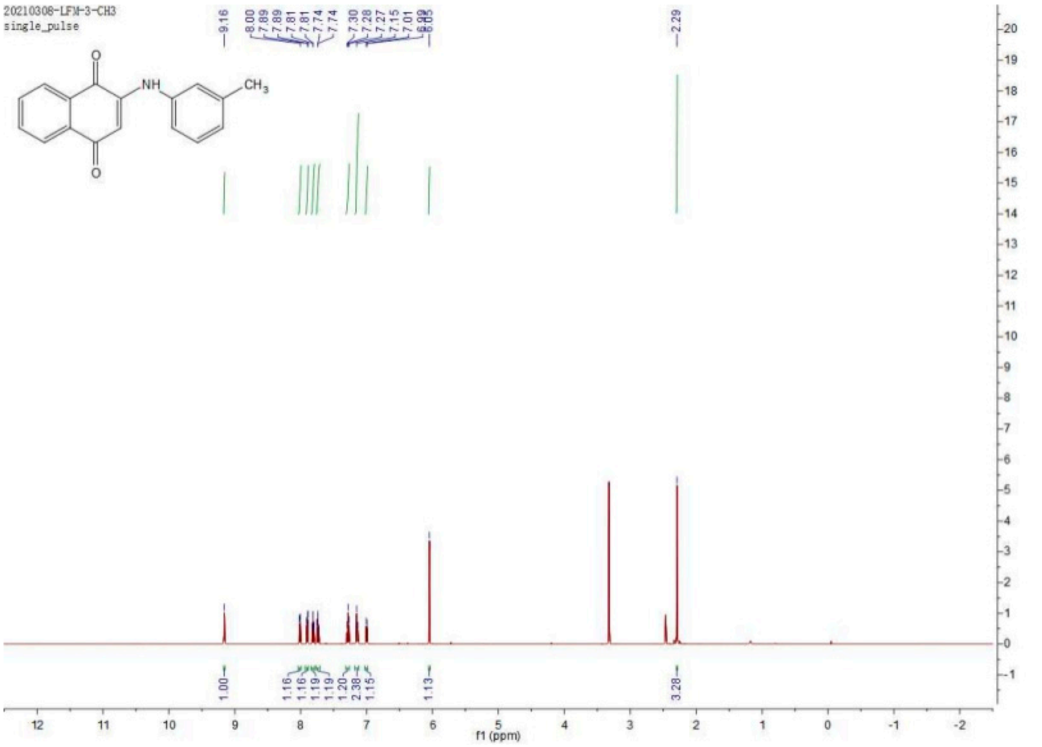

Fig S8. The ^{1}H NMR Spectrum of compound **5b**

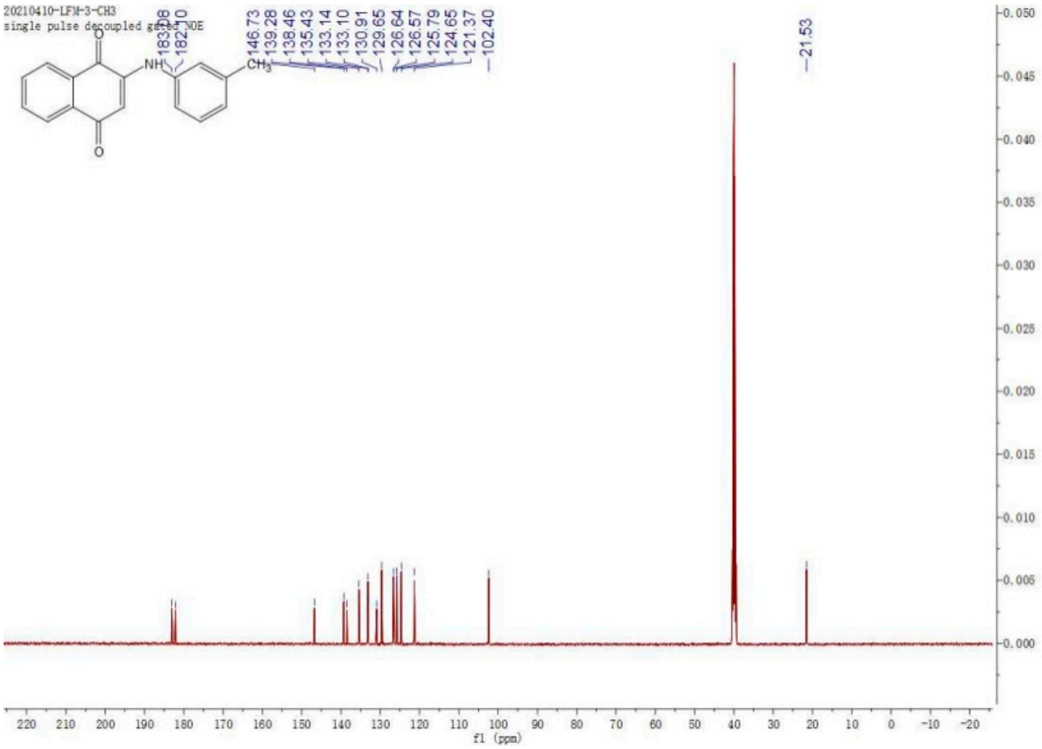

Fig S9. The ^{13}C NMR Spectrum of compound **5b**

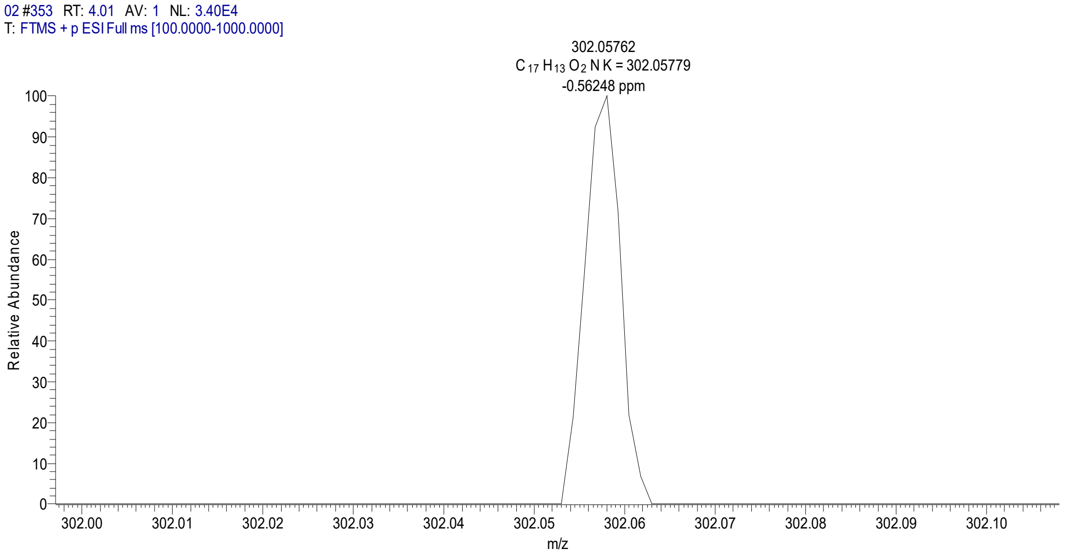

Fig S10. The HRMS spectrum of compound **5b**

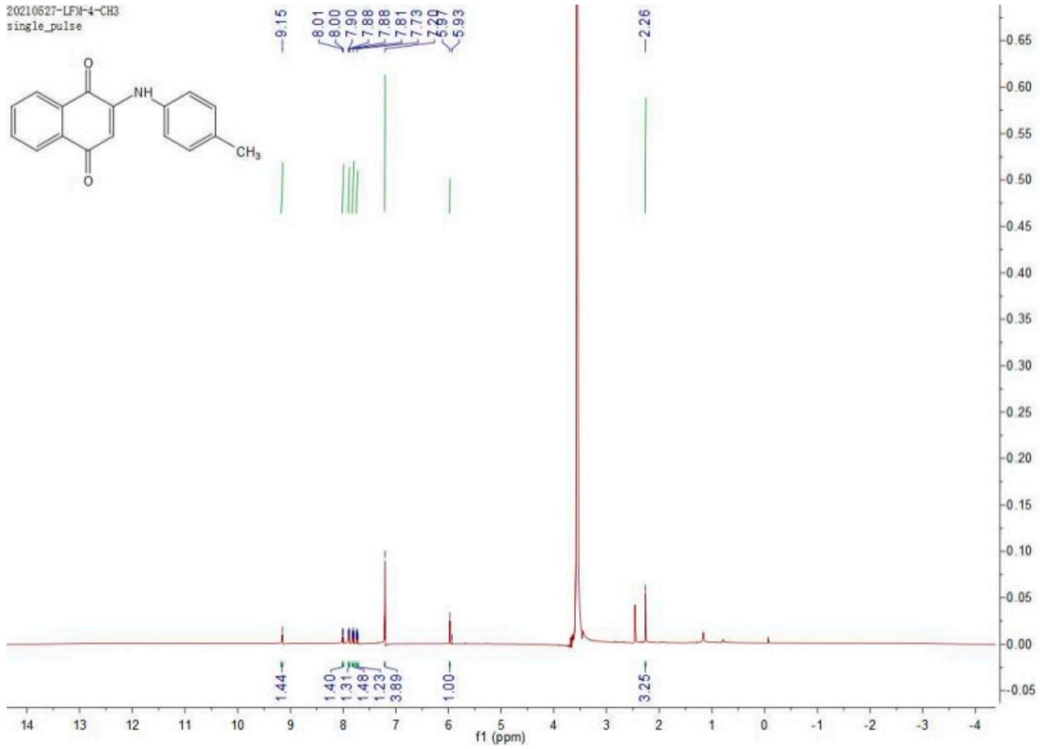

Fig S11. The ^{1}H NMR Spectrum of compound **5c**

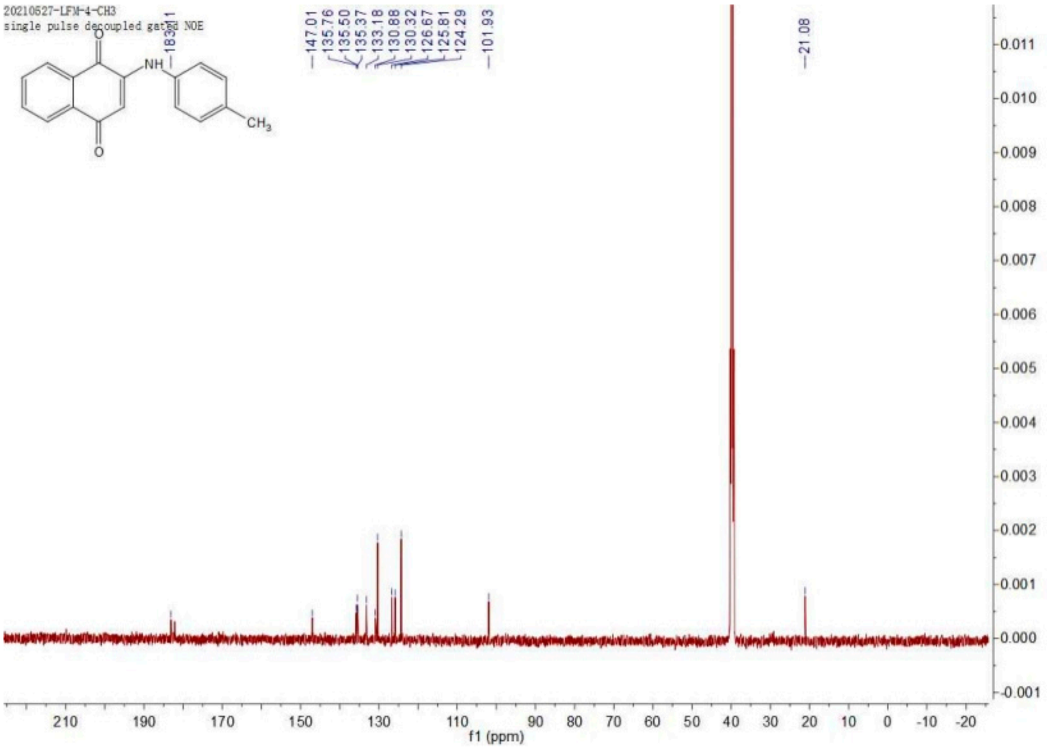

Fig S12. The ^{13}C NMR Spectrum of compound **5c**

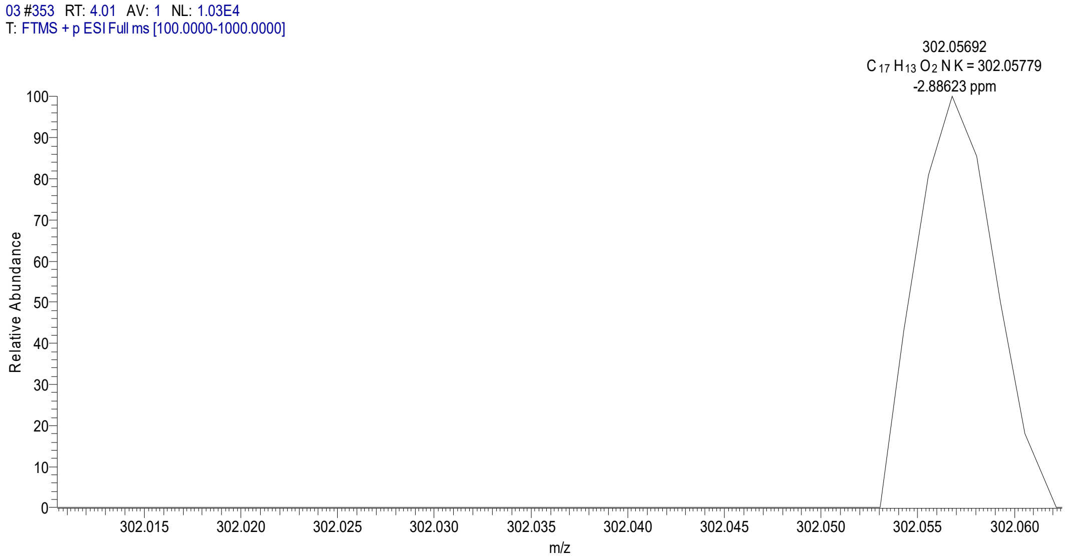

Fig S13. The HRMS spectrum of compound **5c**

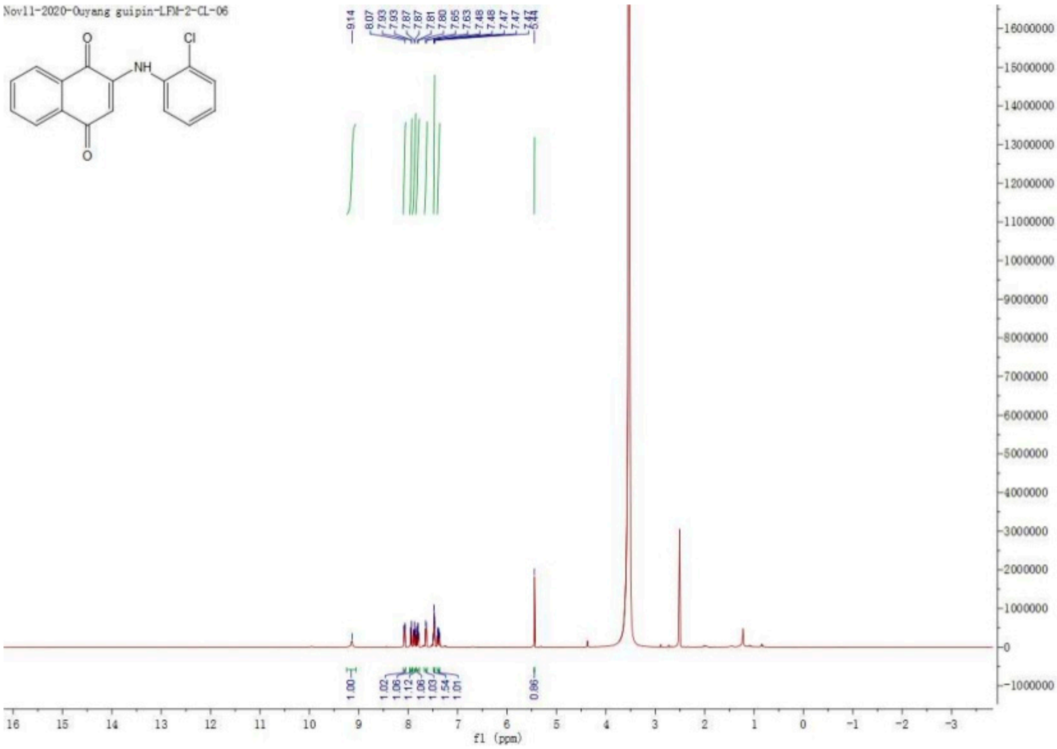

Fig S14. The ^{1}H NMR Spectrum of compound **5d**

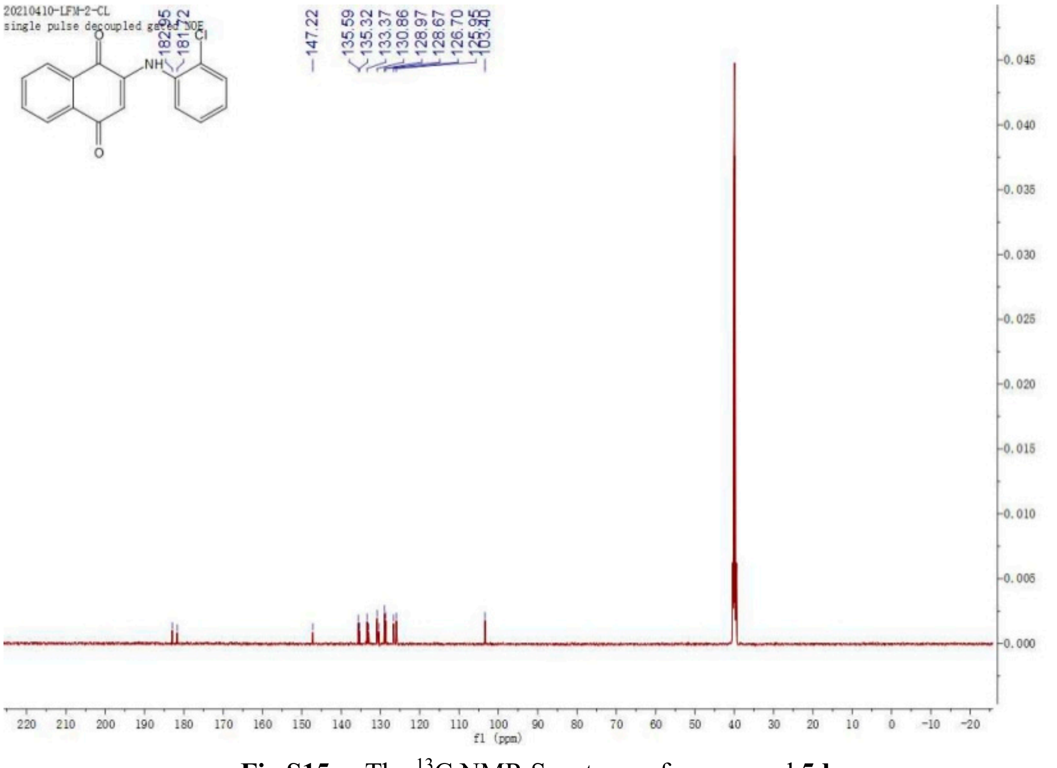

Fig S15. The ^{13}C NMR Spectrum of compound **5d**

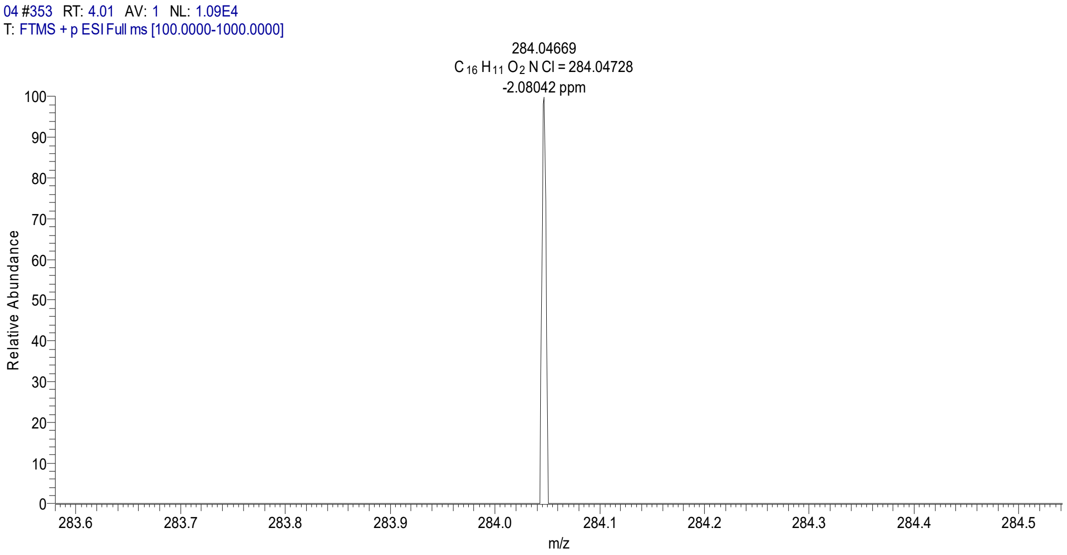

Fig S16. The HRMS spectrum of compound **5d**

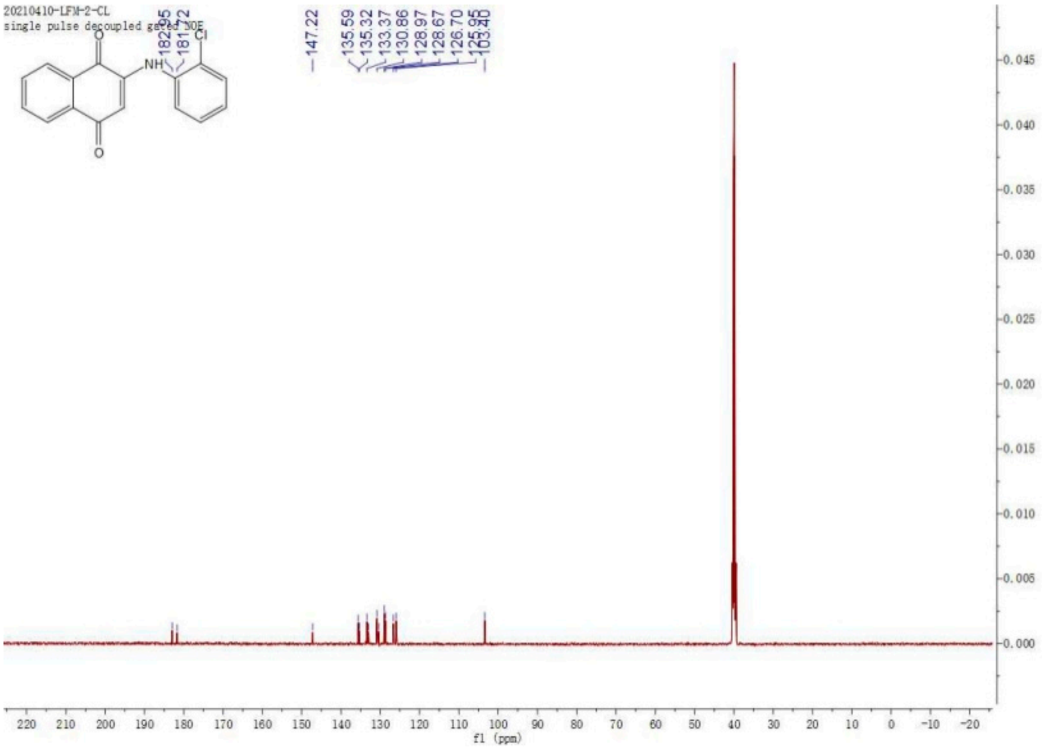

Fig S17. The ^{13}C NMR Spectrum of compound **5d**

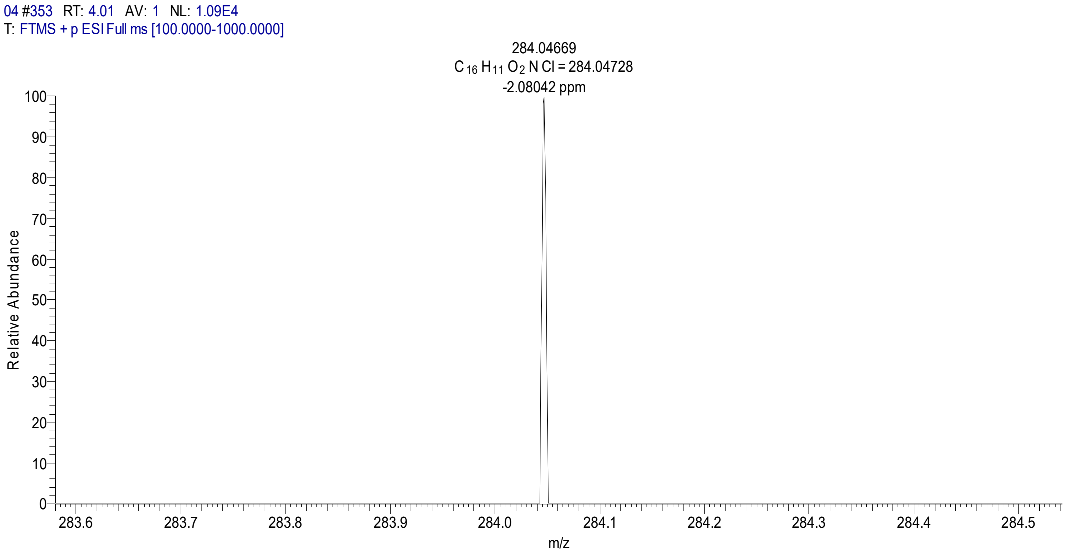

Fig S18. The HRMS spectrum of compound **5d**

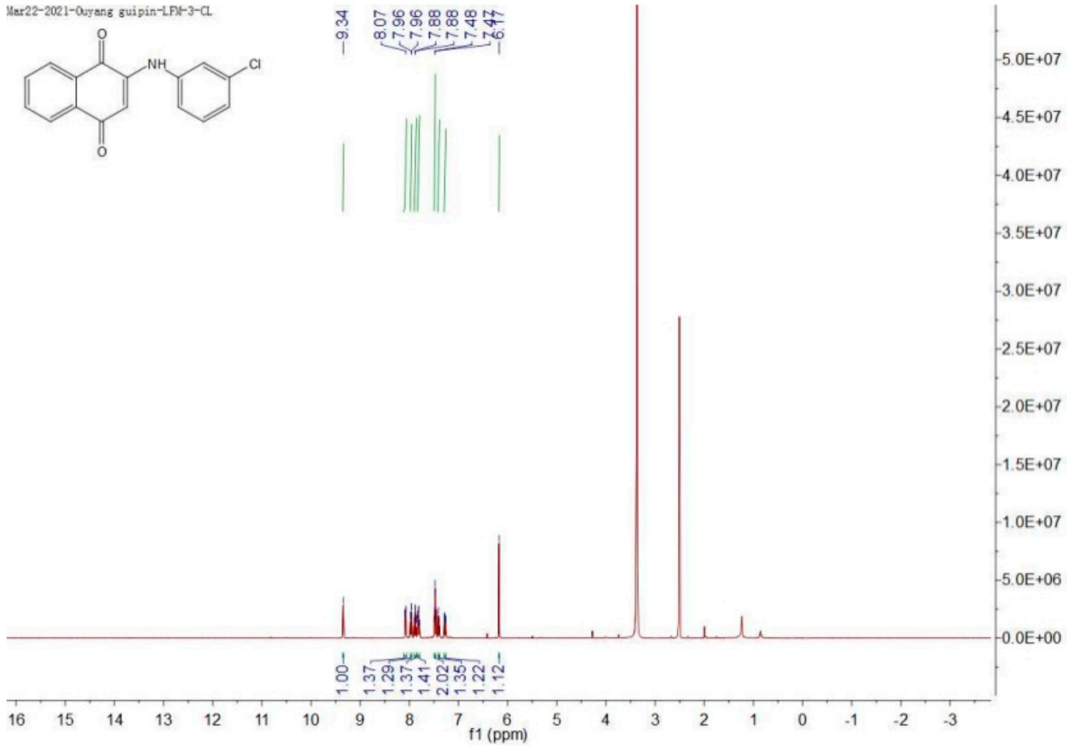

Fig S19. The ^{1}H NMR Spectrum of compound **5e**

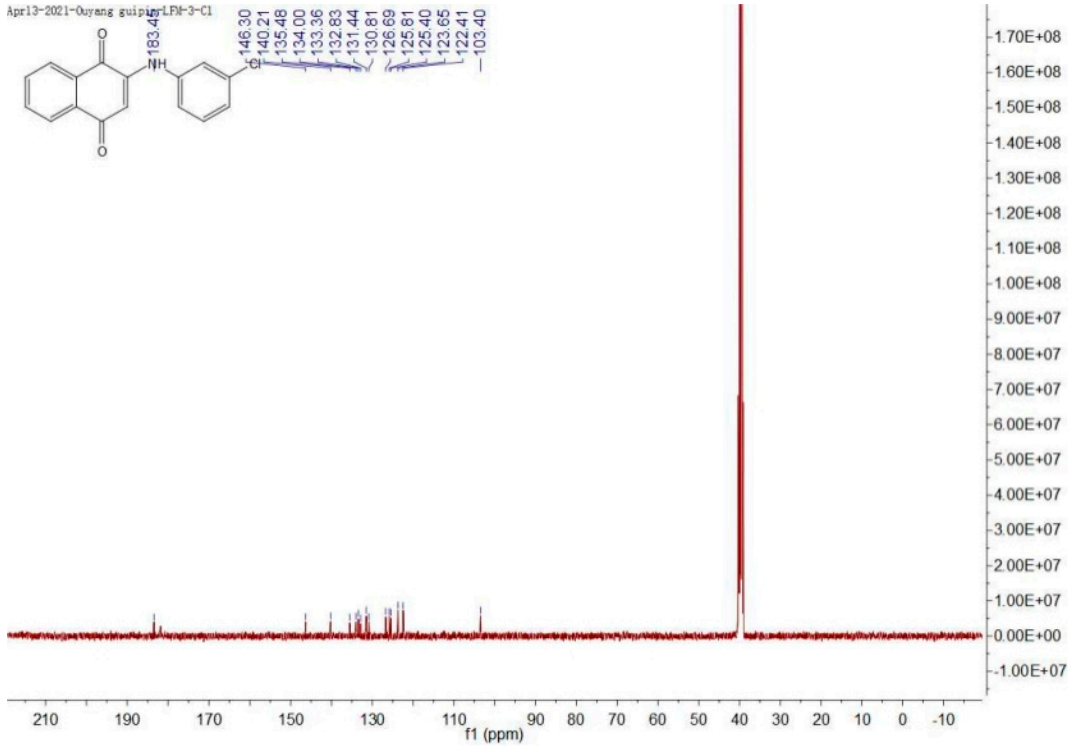

Fig S20. The ^{13}C NMR Spectrum of compound **5e**

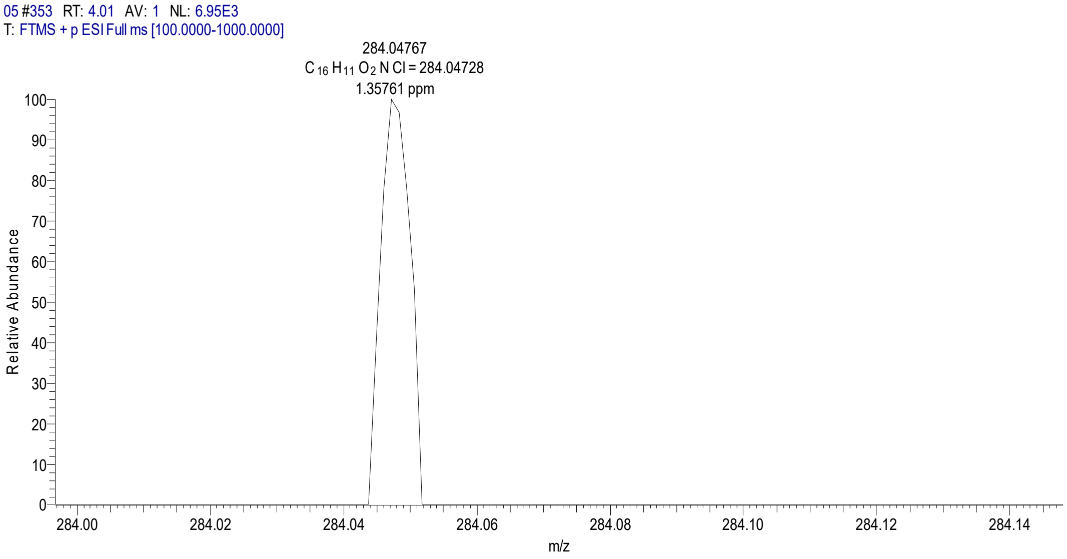

Fig S21. The HRMS spectrum of compound **5e**

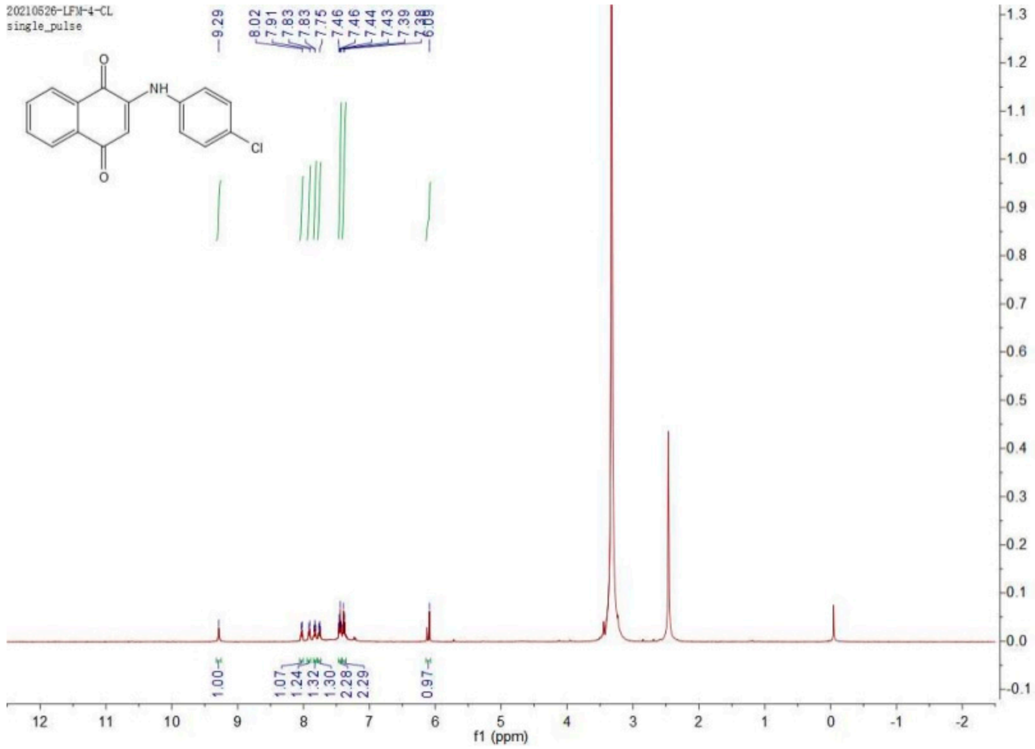

Fig S22. The ^{1}H NMR Spectrum of compound **5f**

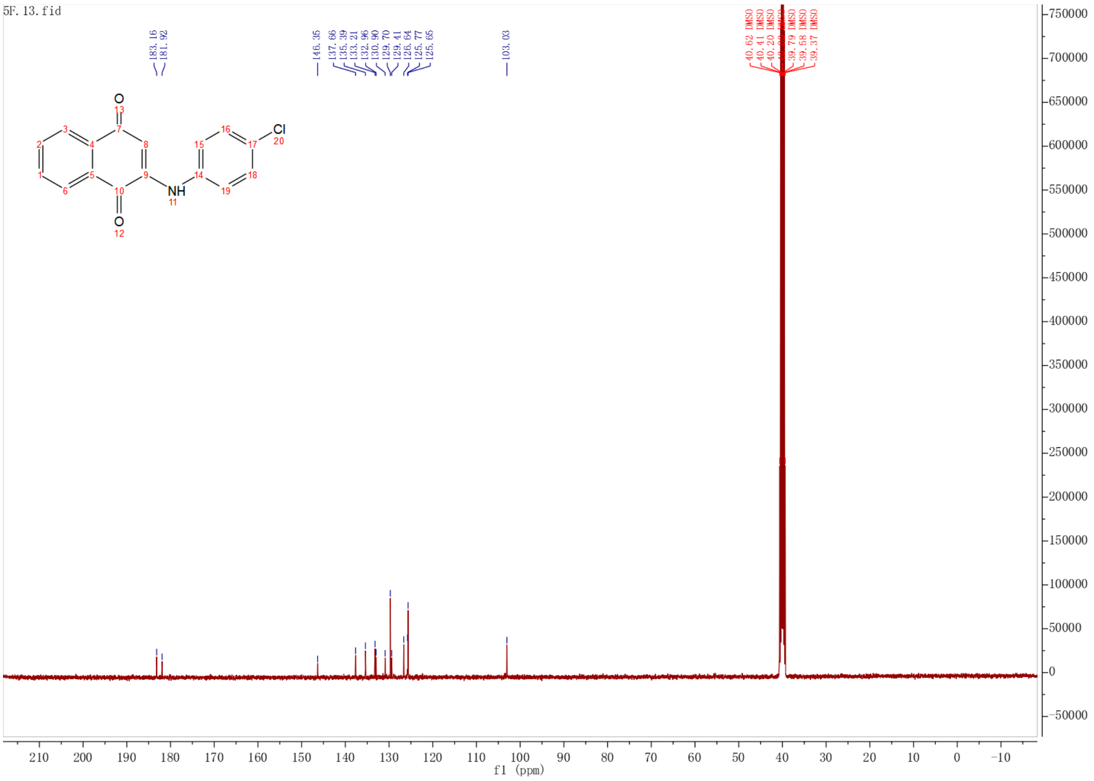

Fig S23. The ^{13}C NMR Spectrum of compound **5f**

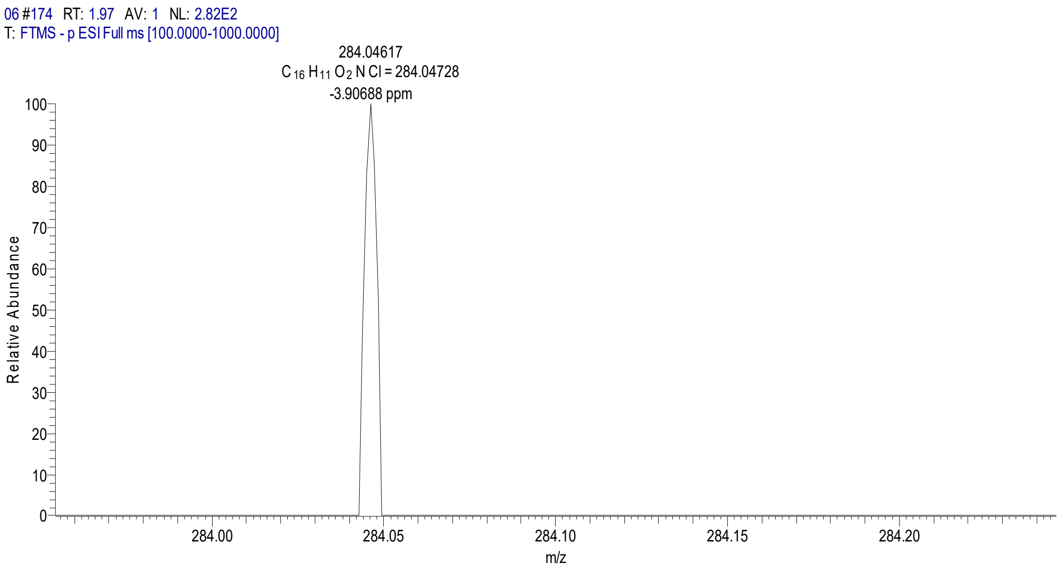

Fig S24. The HRMS spectrum of compound **5f**

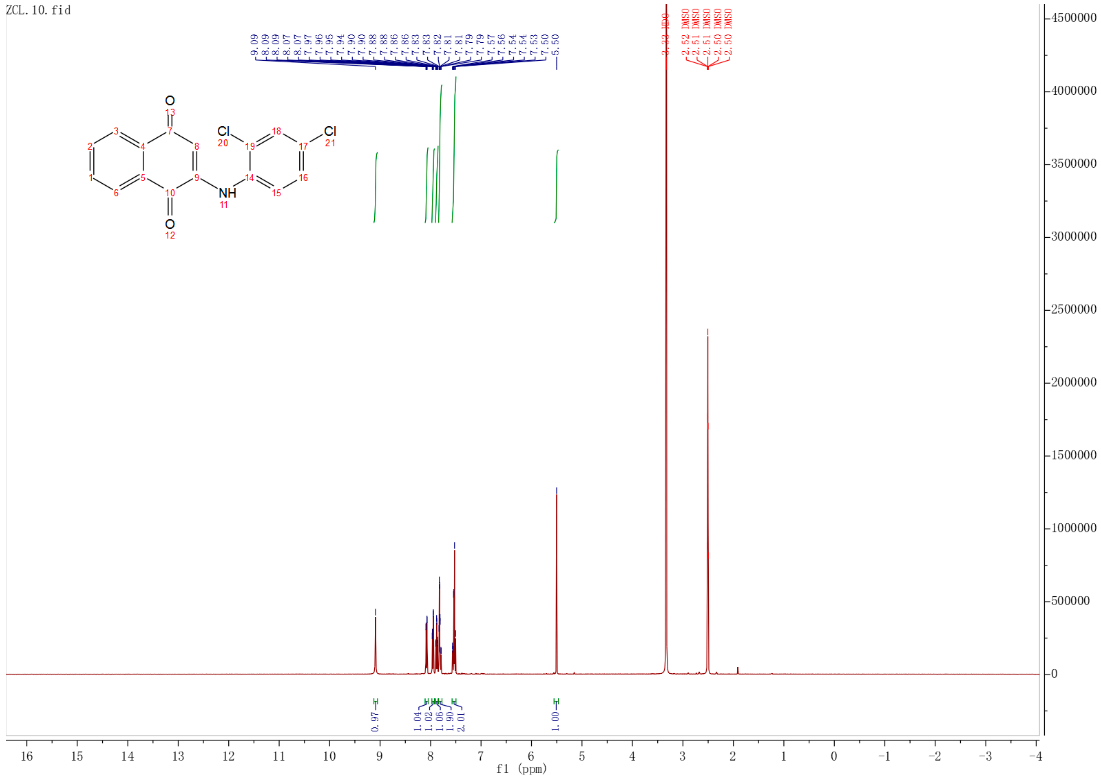

Fig S25. The ^{1}H NMR Spectrum of compound **5g**

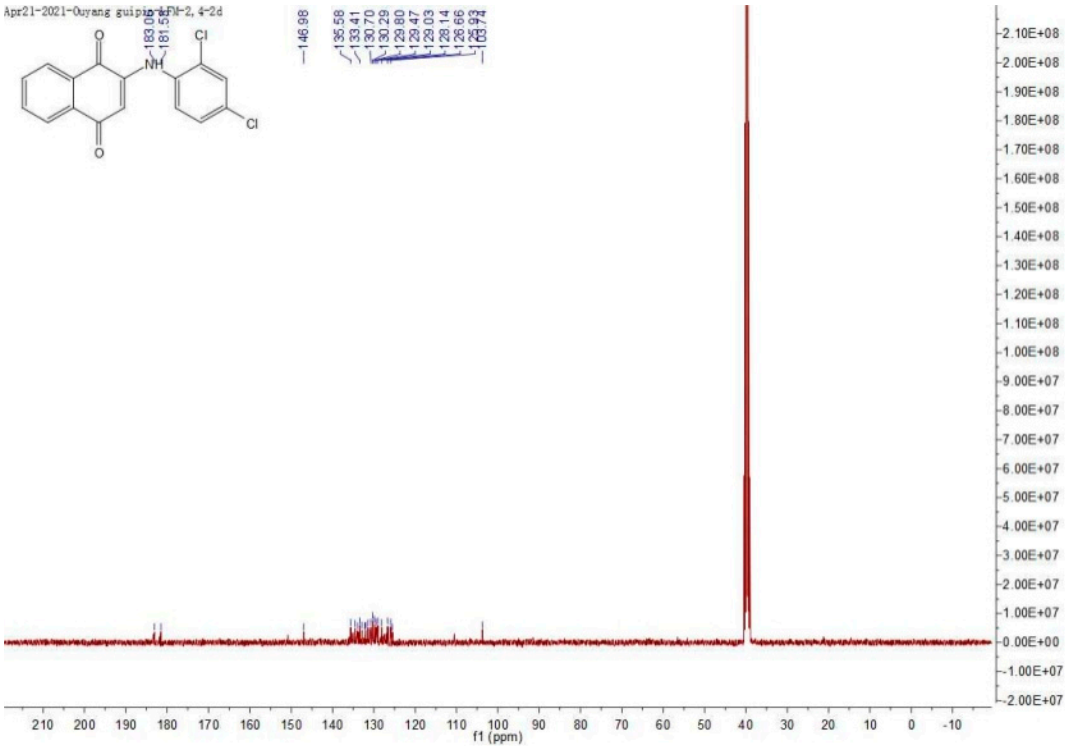

Fig S26. The ^{13}C NMR Spectrum of compound **5g**

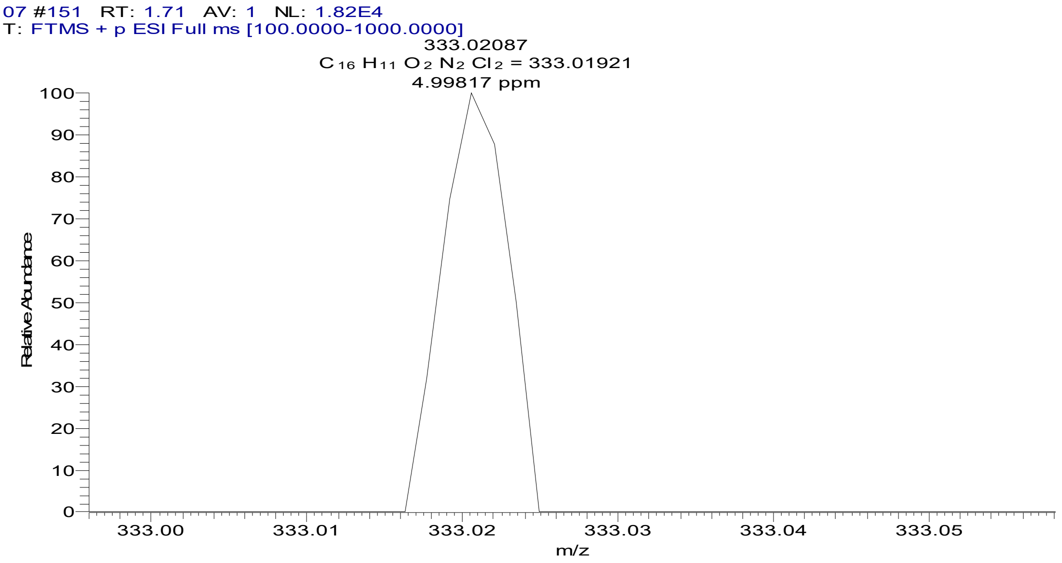

Fig S27. The HRMS spectrum of compound **5g**

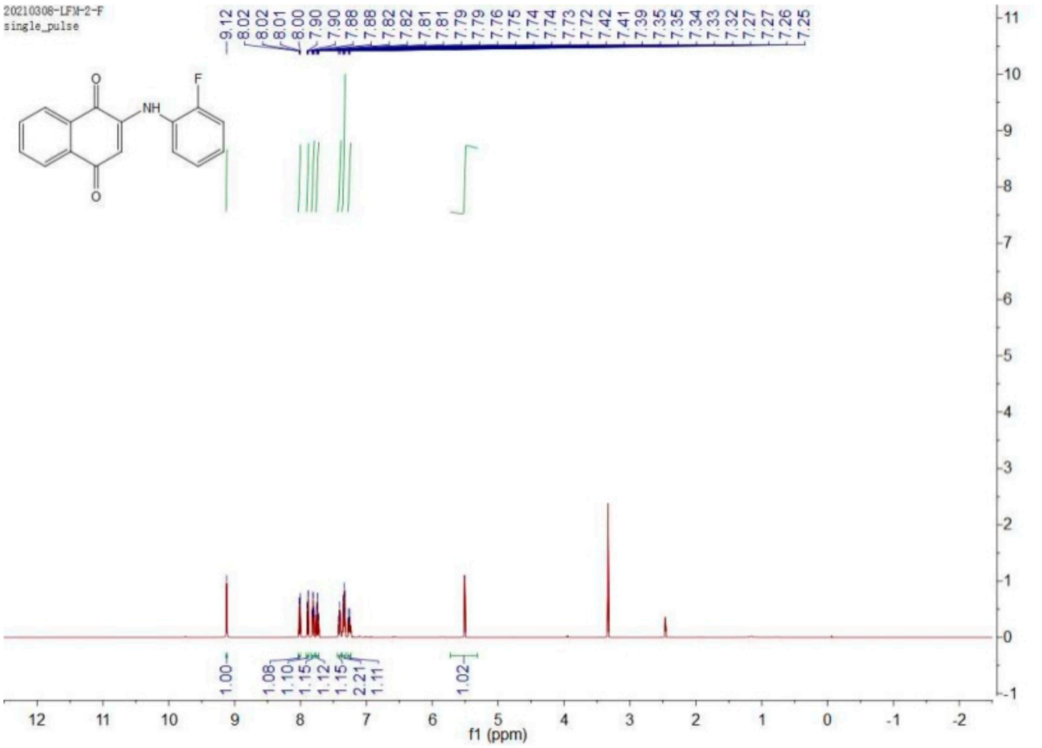

Fig S28. The ^{1}H NMR Spectrum of compound **5h**

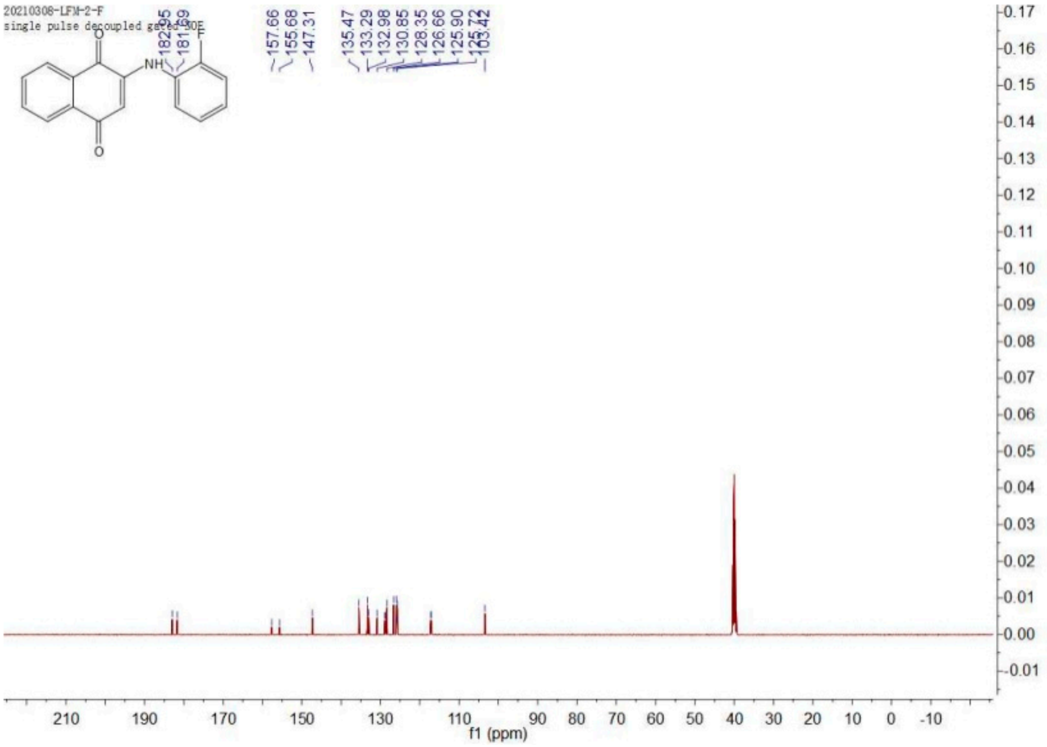

Fig S29. The ^{13}C NMR Spectrum of compound **5h**

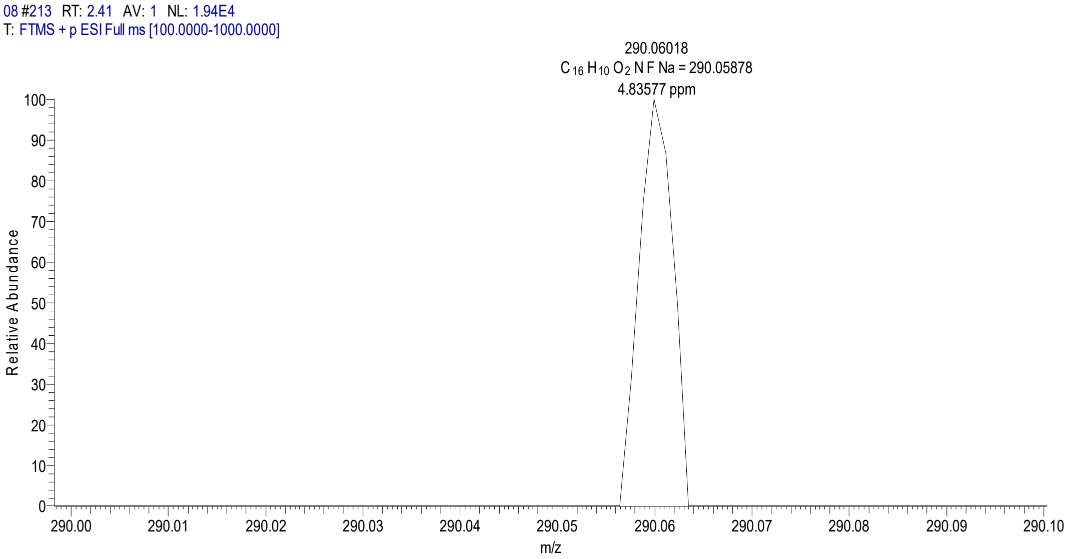

Fig S30. The HRMS spectrum of compound **5h**

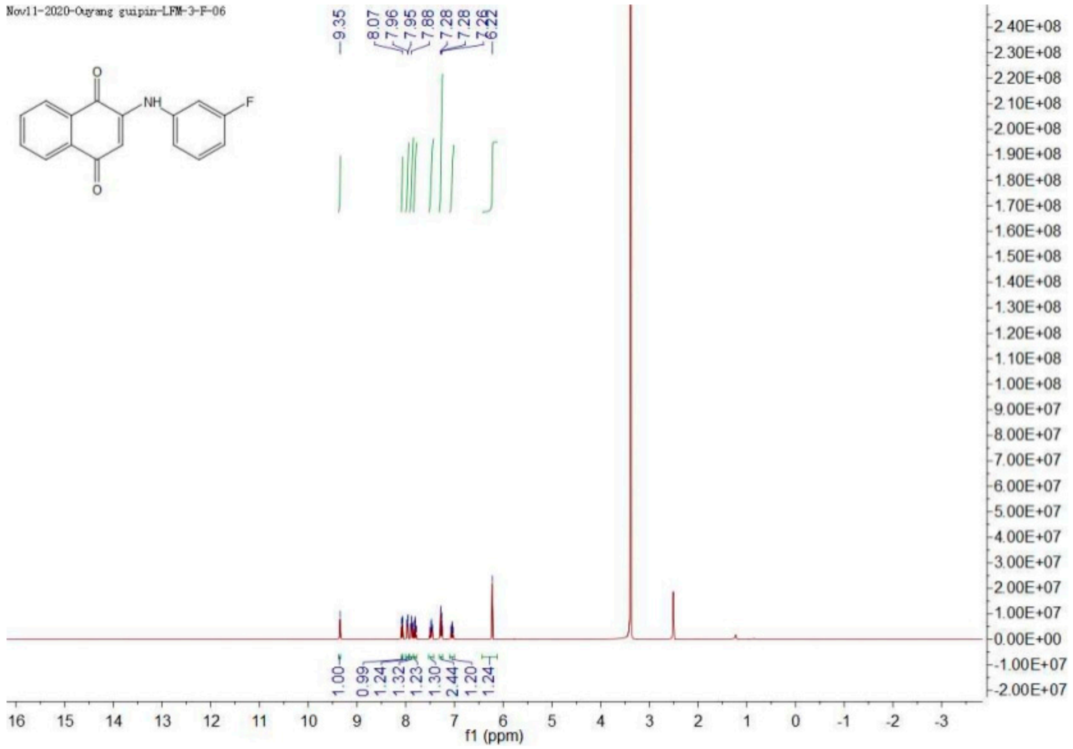

Fig S31. The ^{1}H NMR Spectrum of compound **5i**

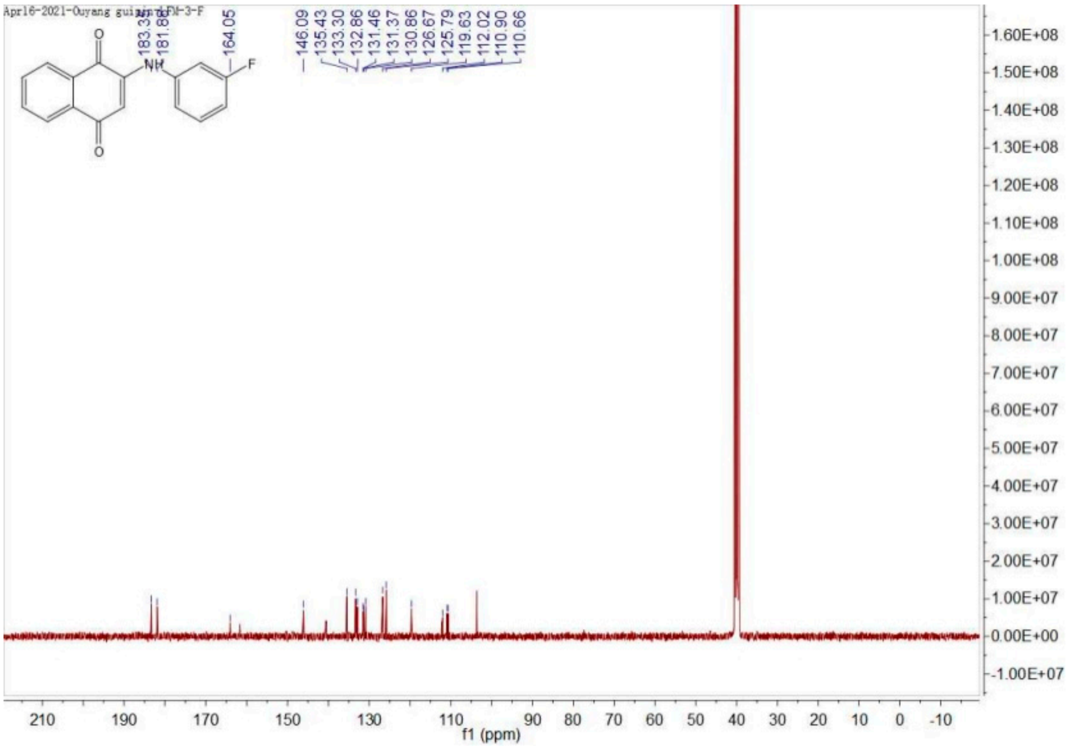

Fig S32. The ^{13}C NMR Spectrum of compound **5i**

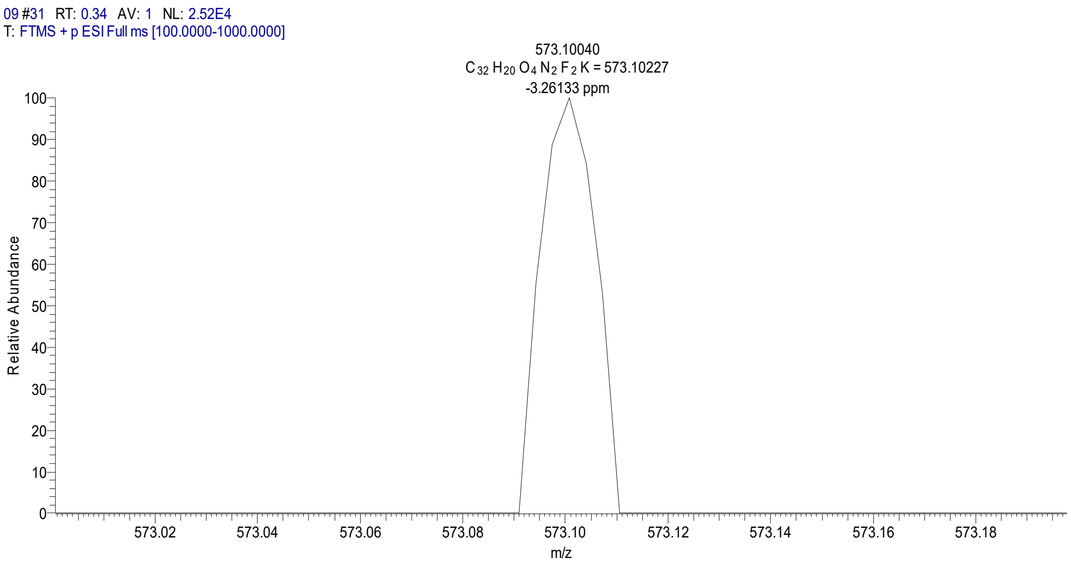

Fig S33. The HRMS spectrum of compound **5i**

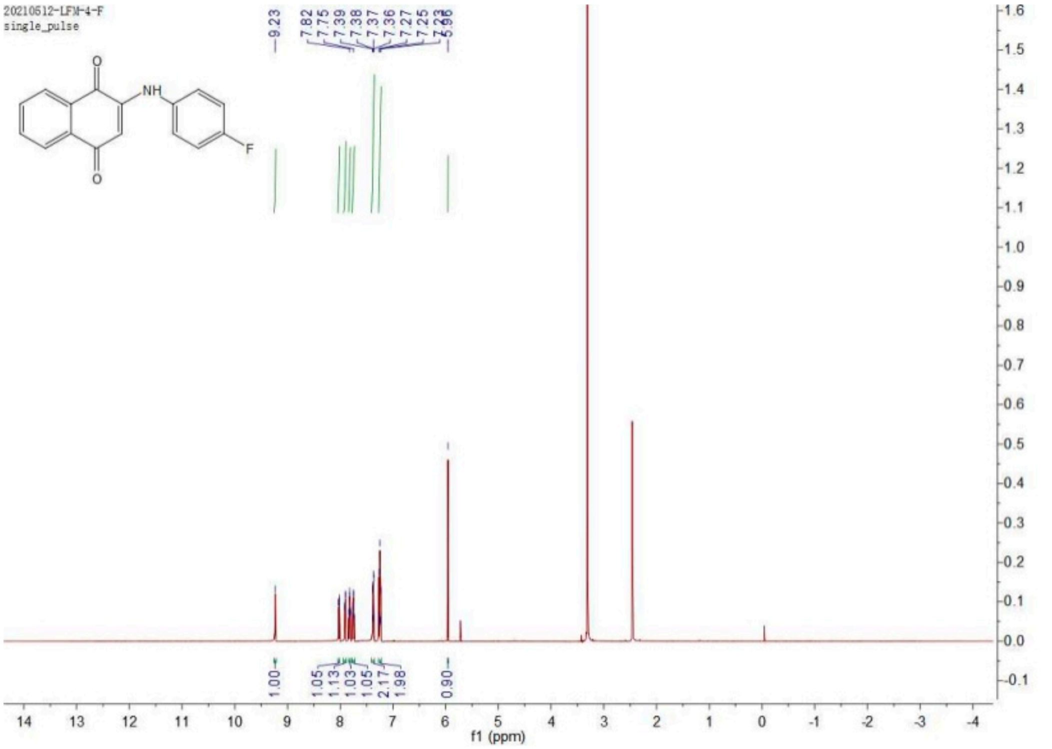

Fig S34. The ^{1}H NMR Spectrum of compound **5j**

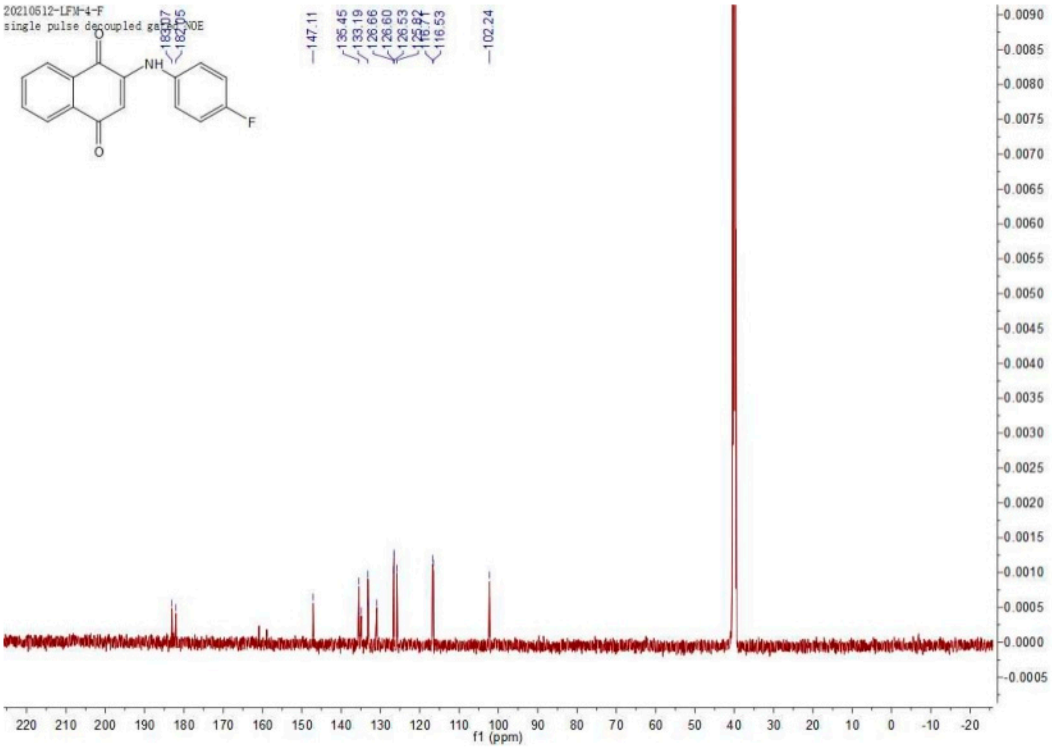

Fig S35. The ^{13}C NMR Spectrum of compound **5j**

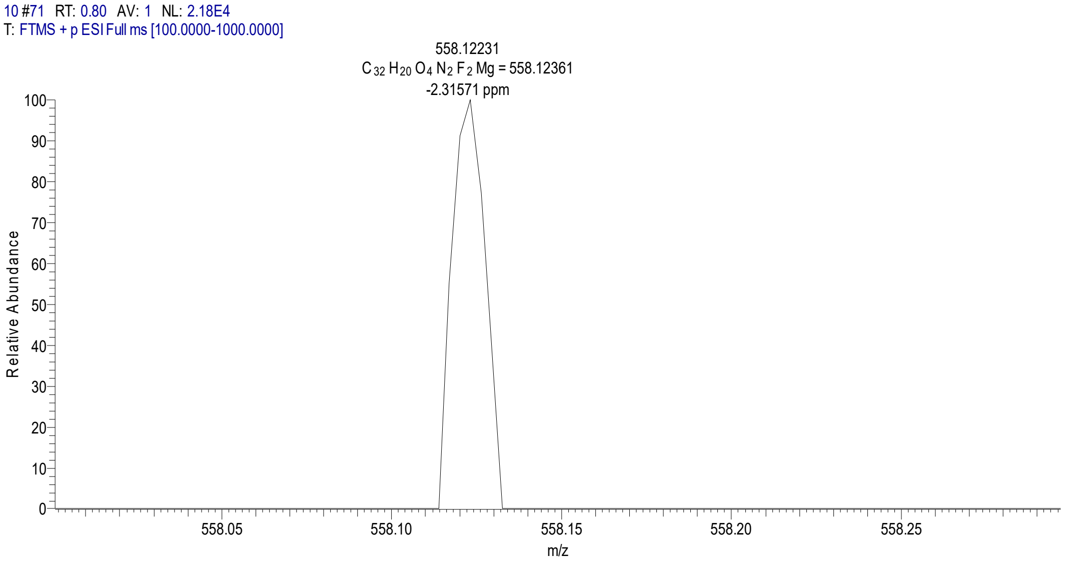

Fig S36. The HRMS spectrum of compound **5j**

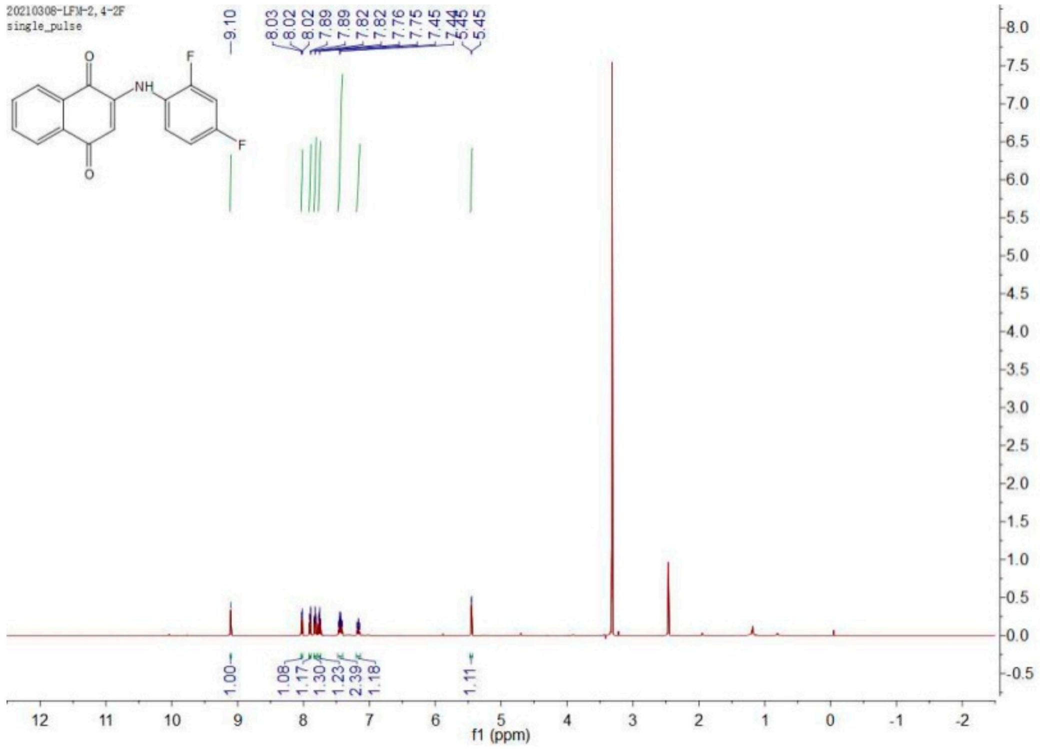

Fig S37. The ^{1}H NMR Spectrum of compound **5k**

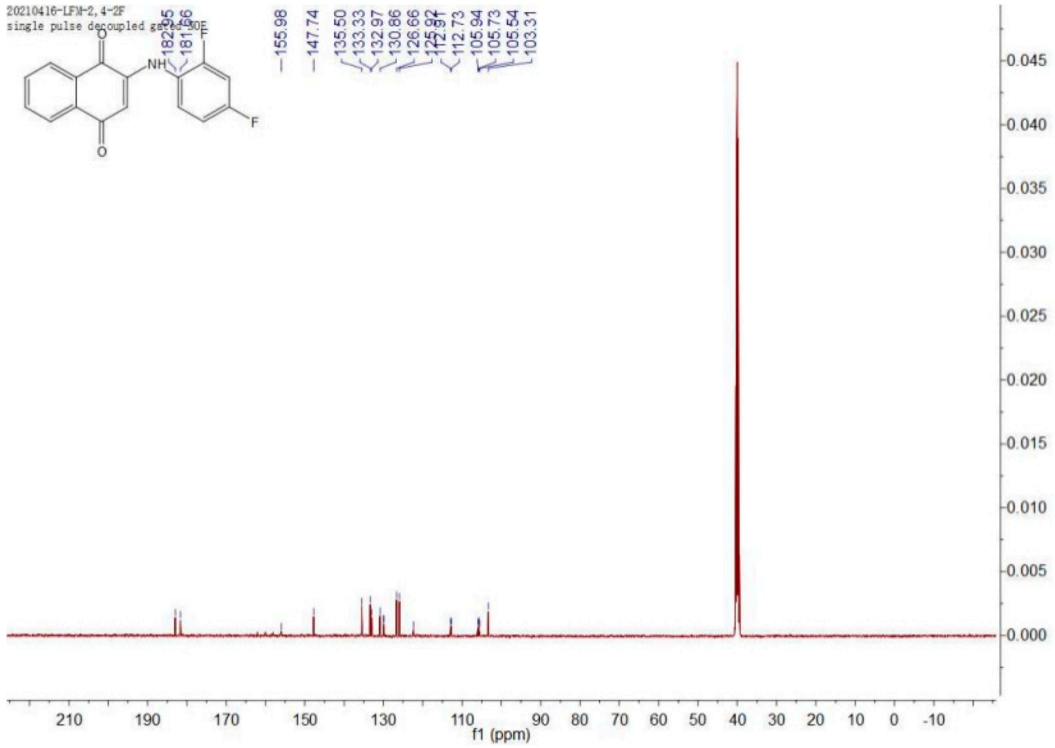

Fig S38. The ^{13}C NMR Spectrum of compound **5k**

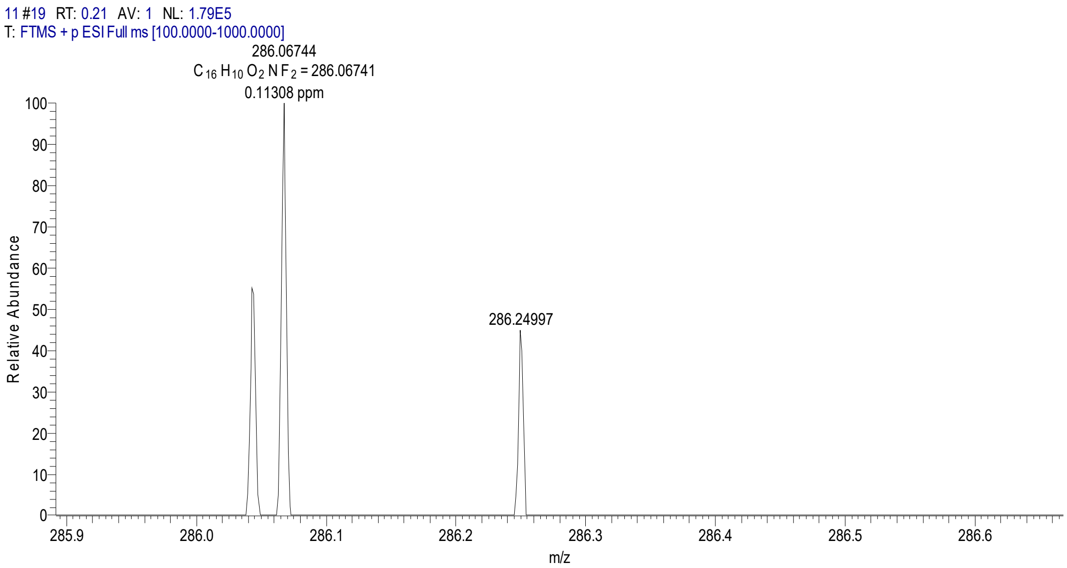

Fig S39. The HRMS spectrum of compound **5k**

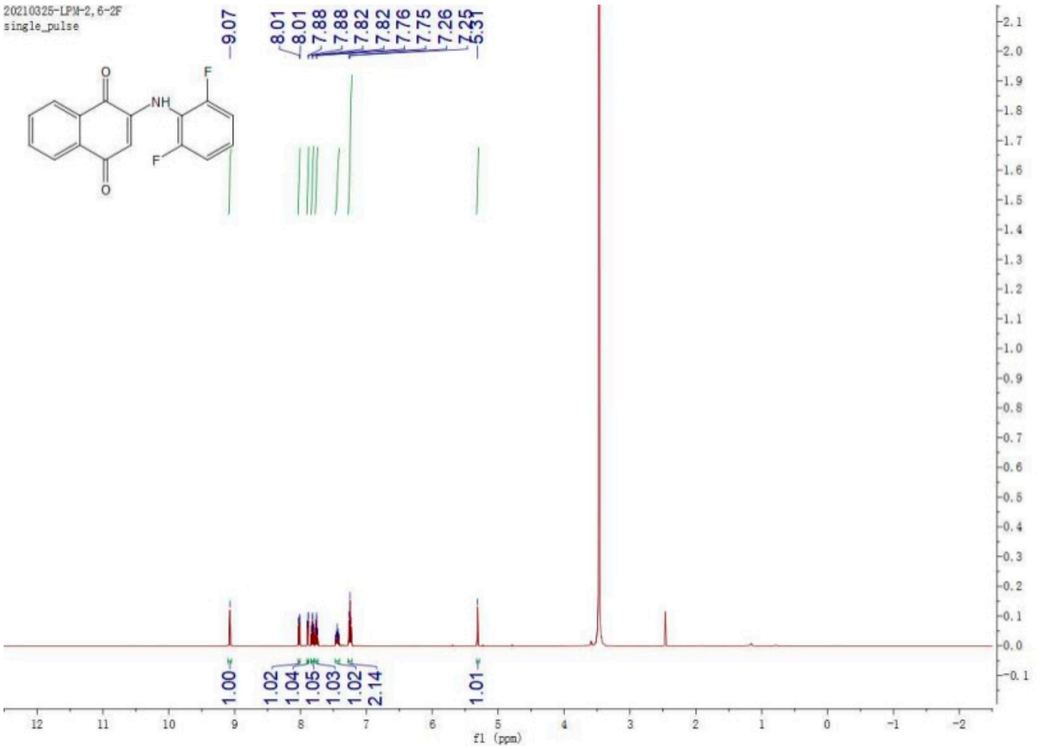

Fig S40. The ^{1}H NMR Spectrum of compound **5l**

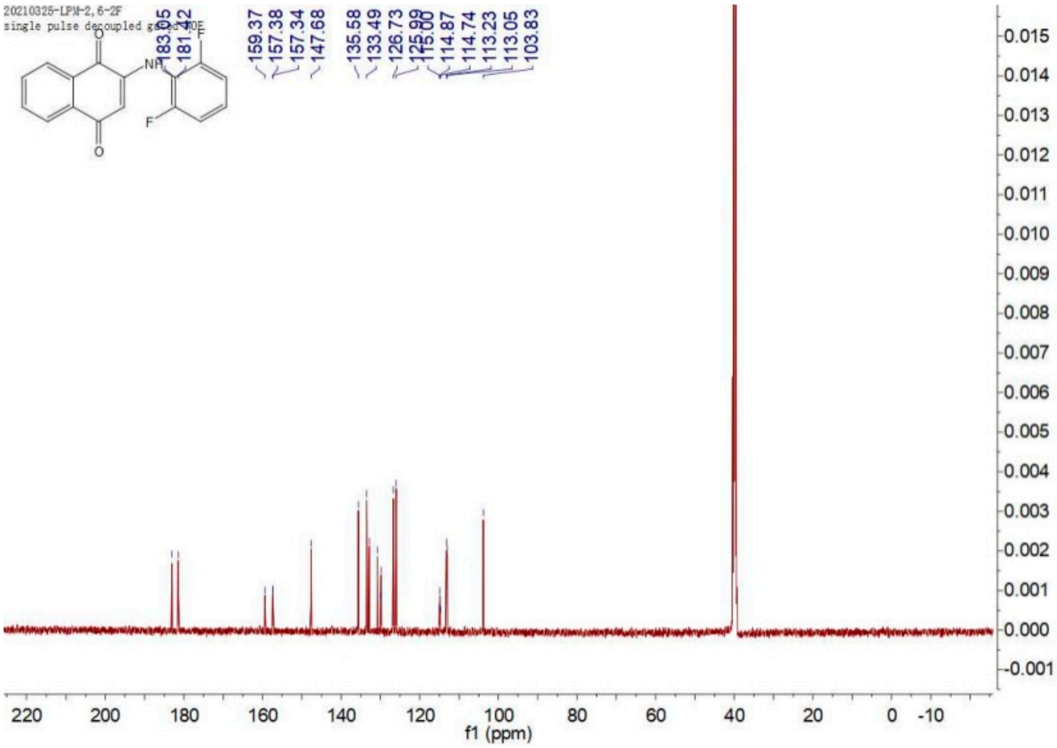

Fig S41. The ^{13}C NMR Spectrum of compound **5l**

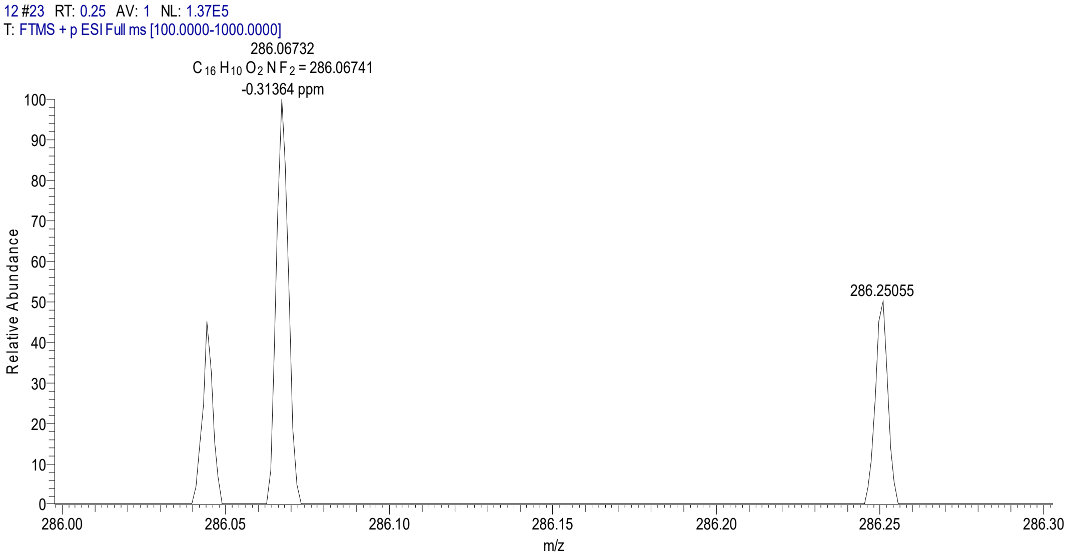

Fig S42. The HRMS spectrum of compound **5l**

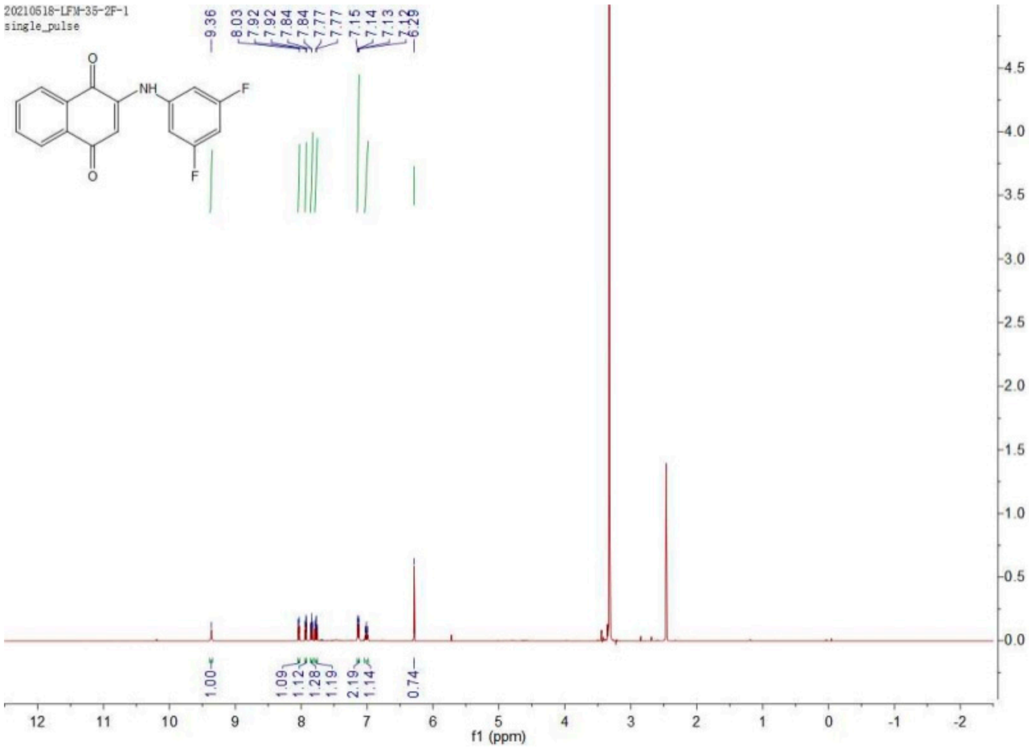

Fig S43. The ^{1}H NMR Spectrum of compound **5m**

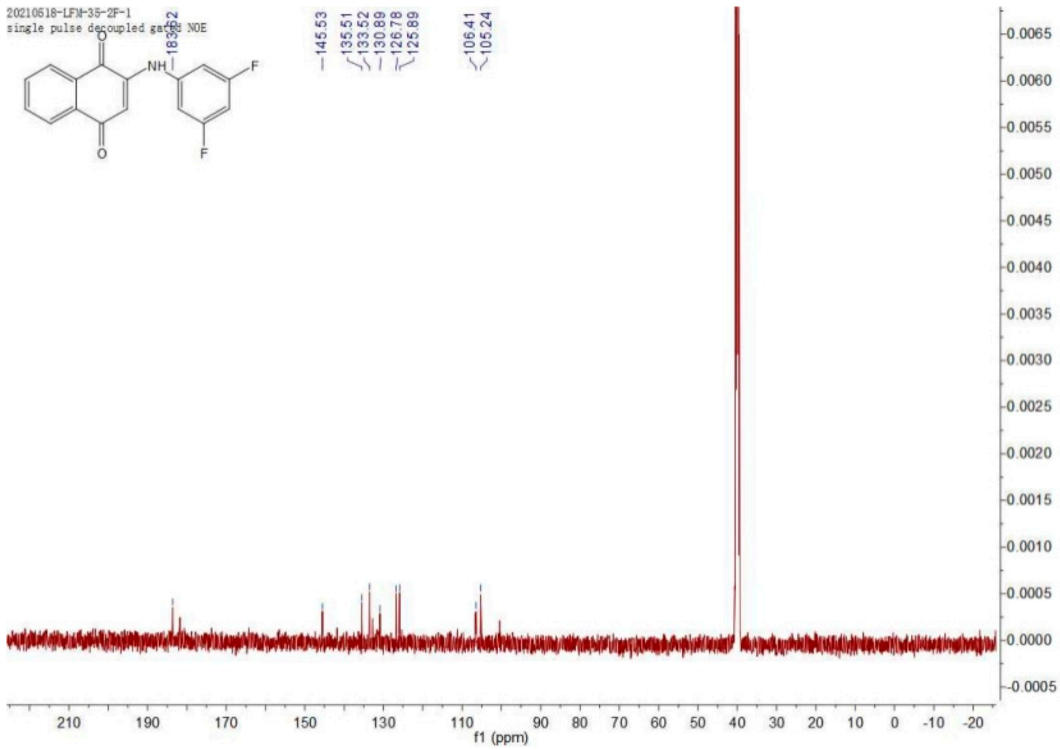

Fig S44. The ^{13}C NMR Spectrum of compound **5m**

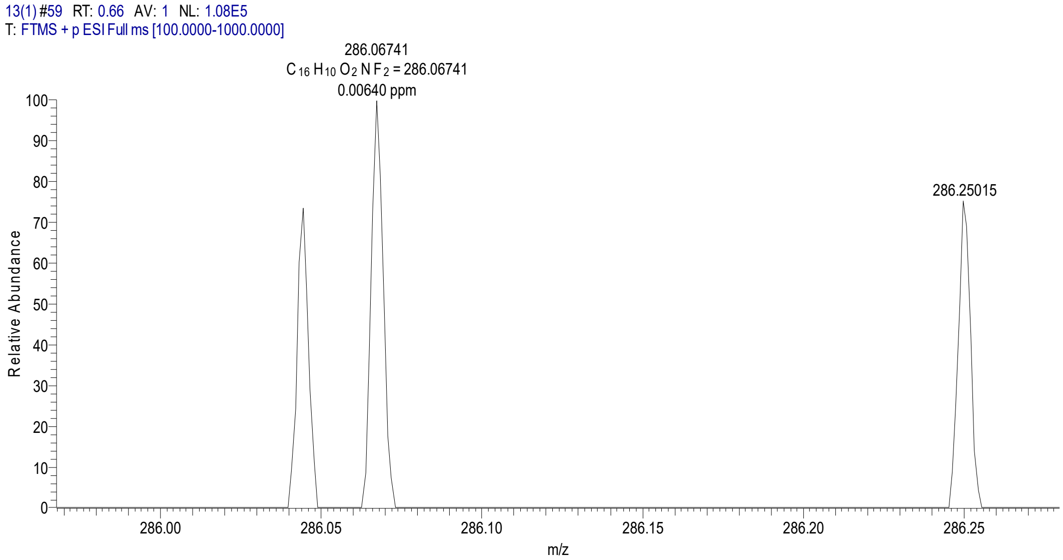

Fig S45. The HRMS spectrum of compound **5m**

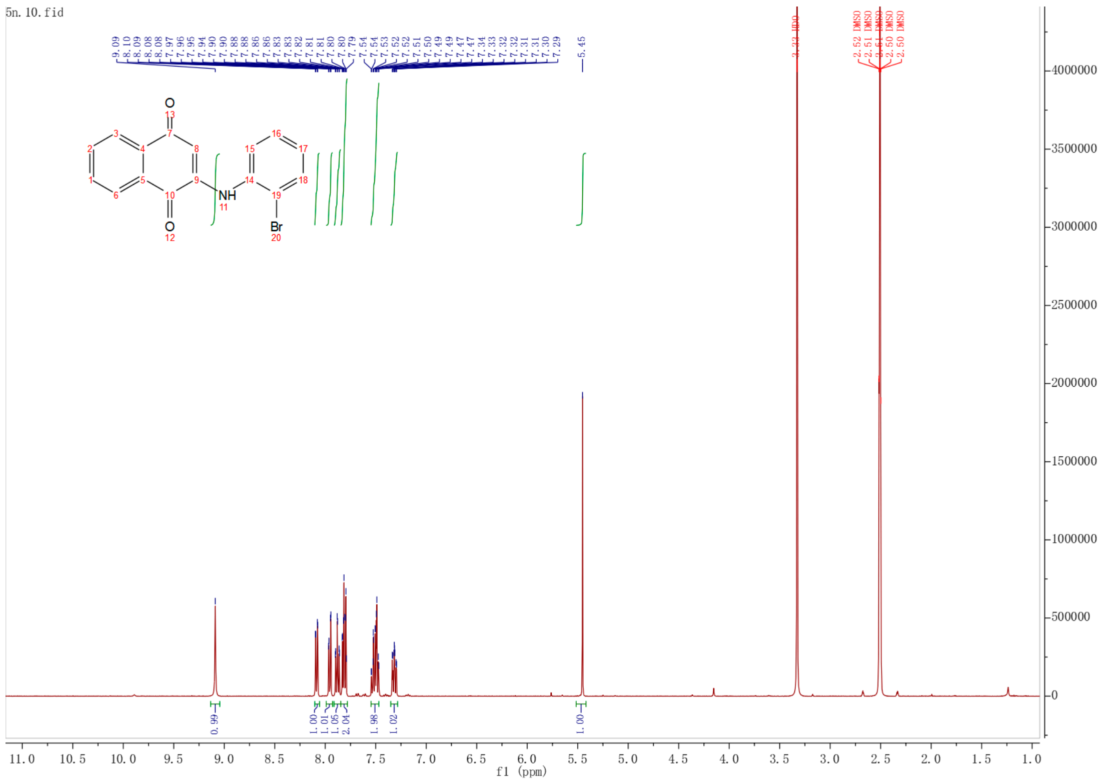

Fig S46. The ^{1}H NMR Spectrum of compound **5n**

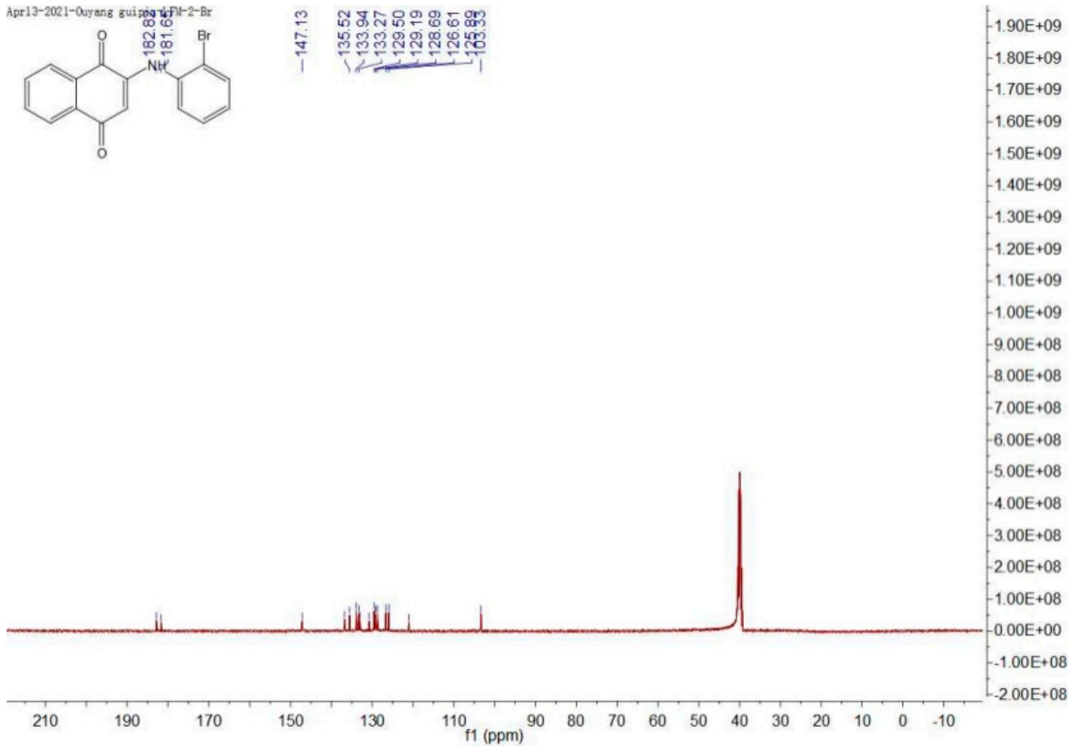

Fig S47. The ^{13}C NMR Spectrum of compound **5n**

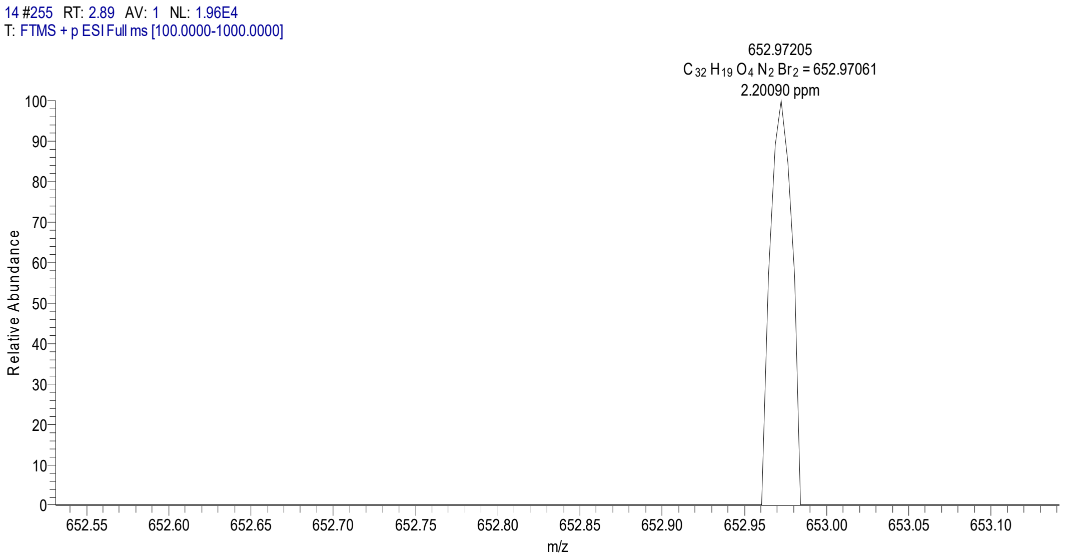

Fig S48. The HRMS spectrum of compound **5n**

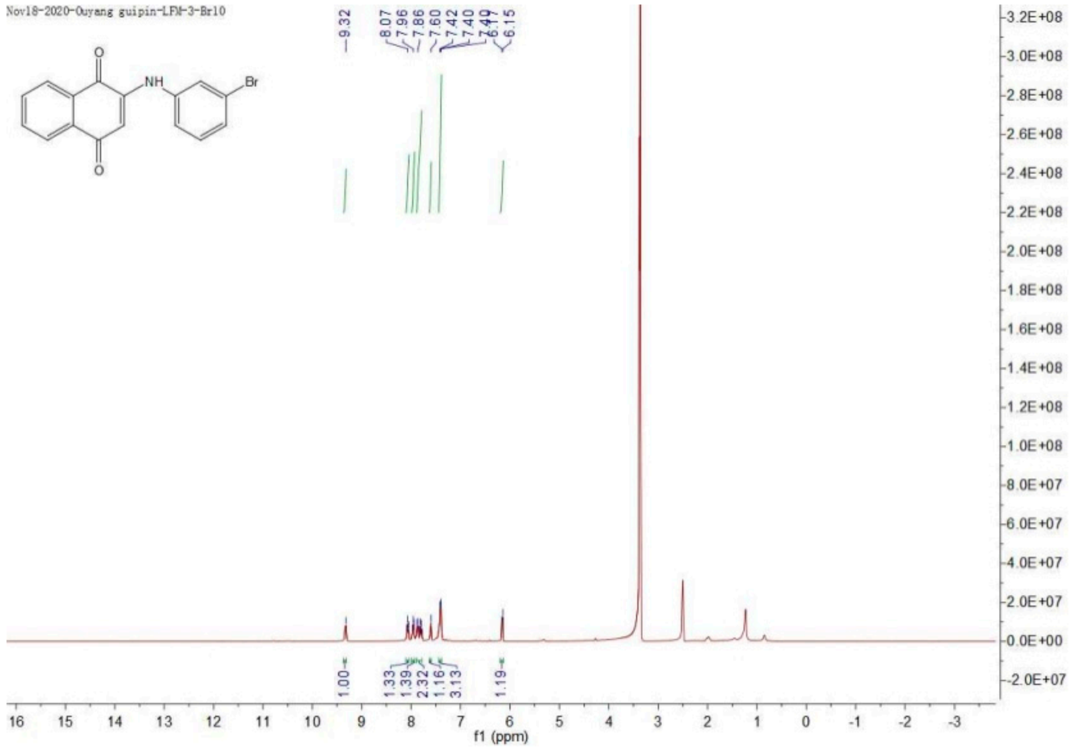

Fig S49. The ^{1}H NMR Spectrum of compound **5o**

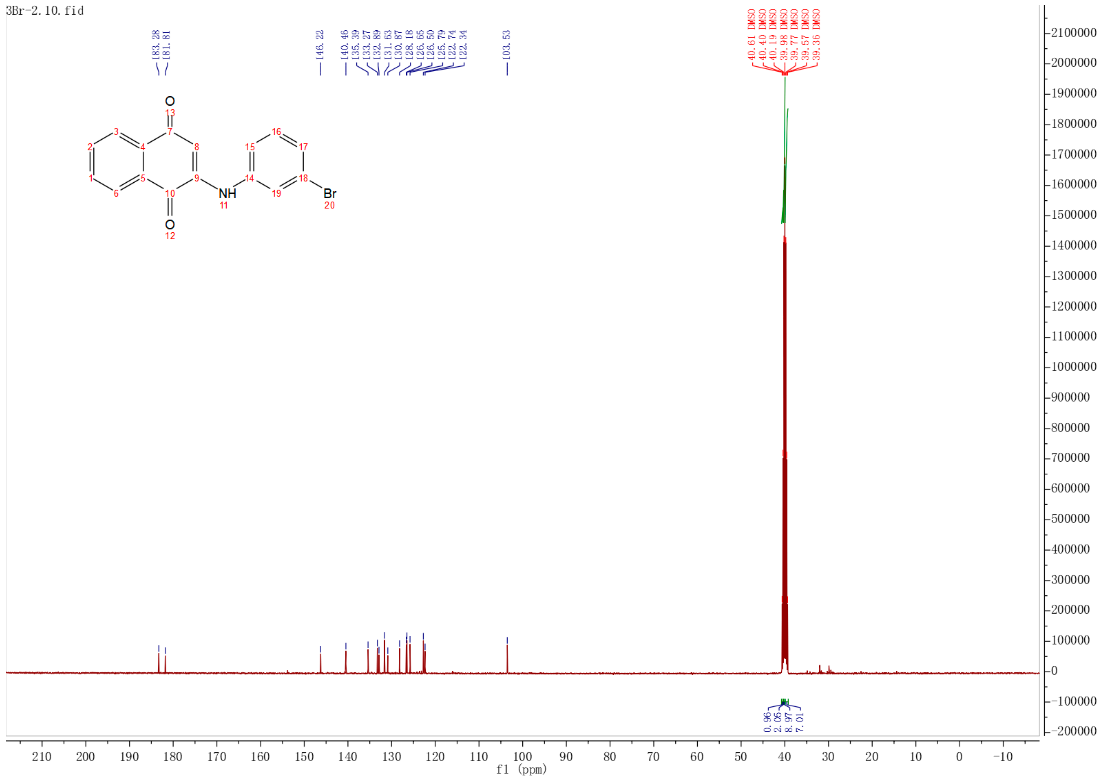

Fig S50. The ^{13}C NMR Spectrum of compound **5o**

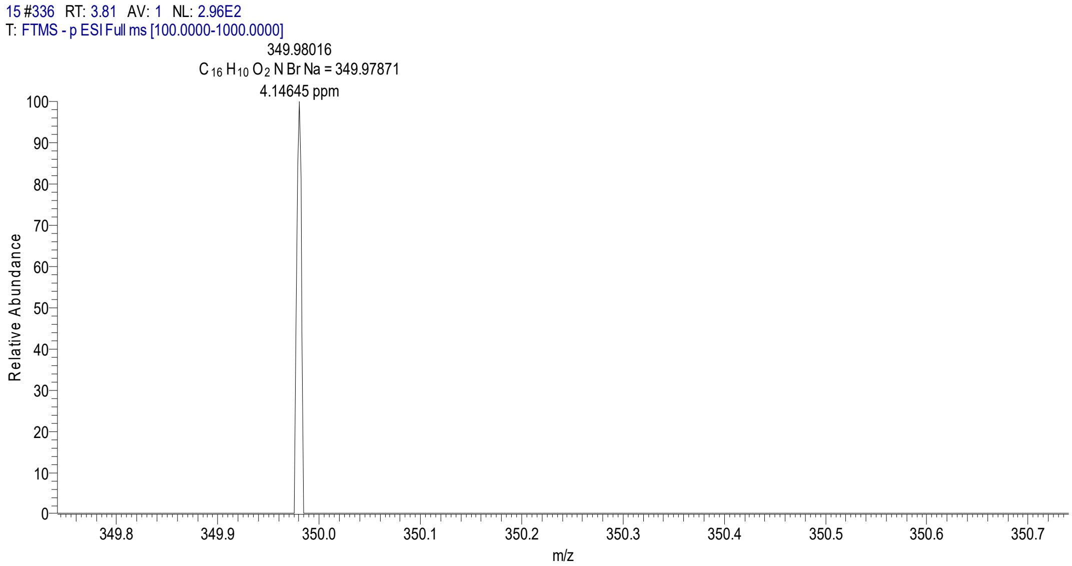

Fig S51. The HRMS spectrum of compound **5o**

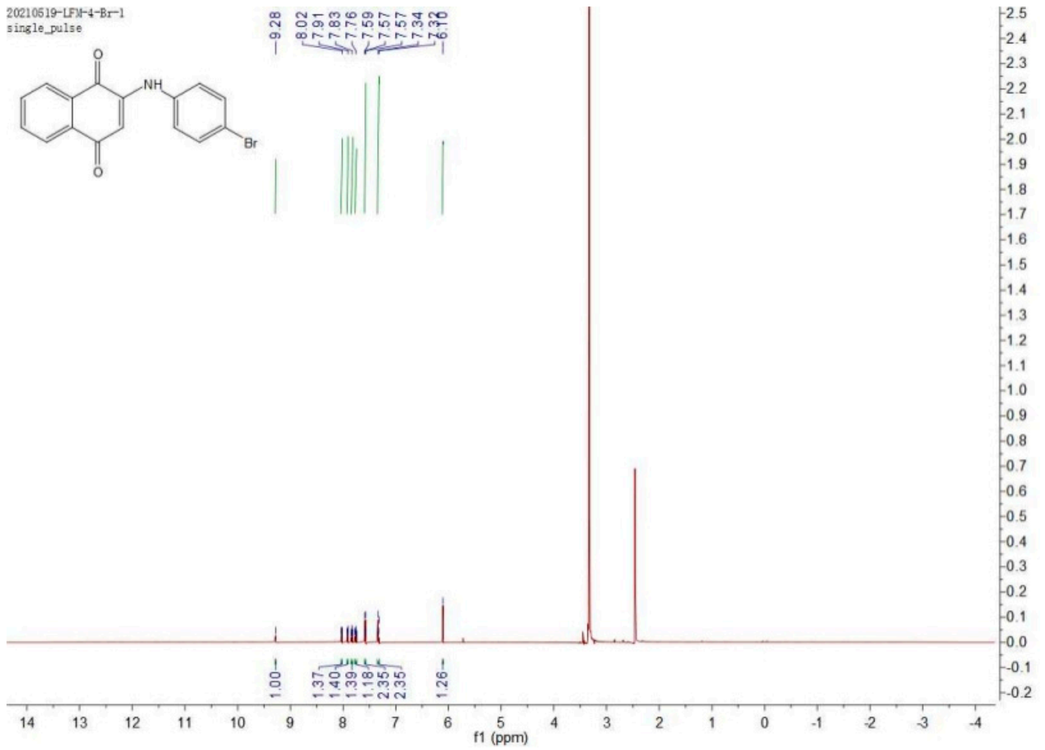

Fig S52. The ^{1}H NMR Spectrum of compound **5p**

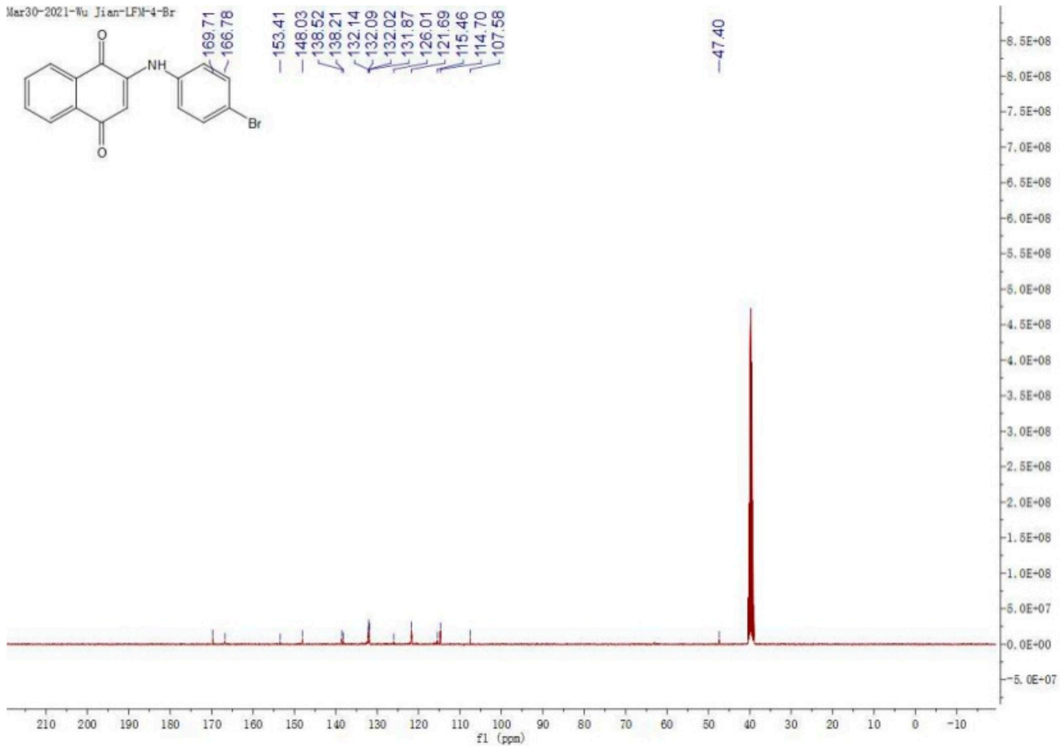

Fig S53. The ^{13}C NMR Spectrum of compound **5p**

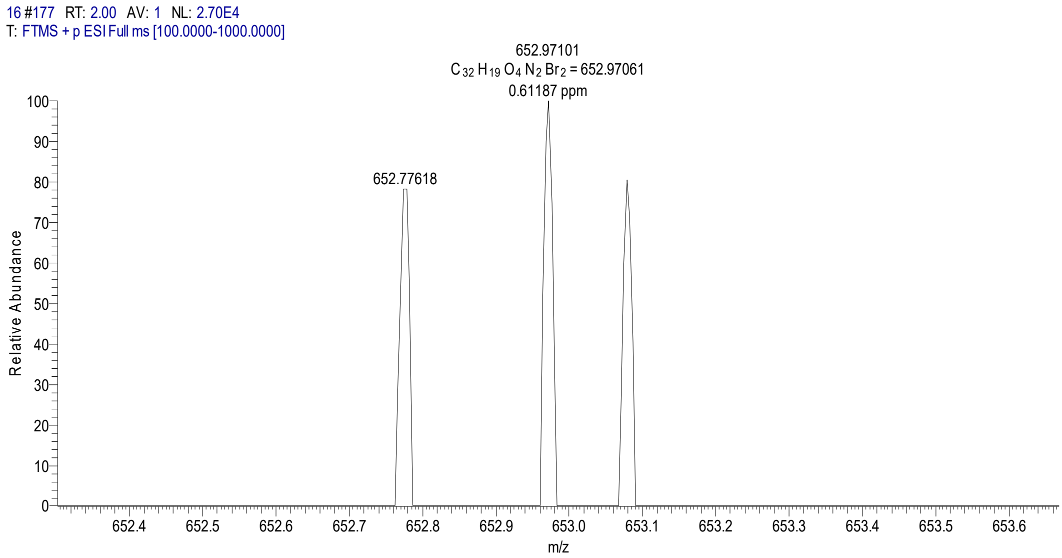

Fig S54. The HRMS spectrum of compound **5p**

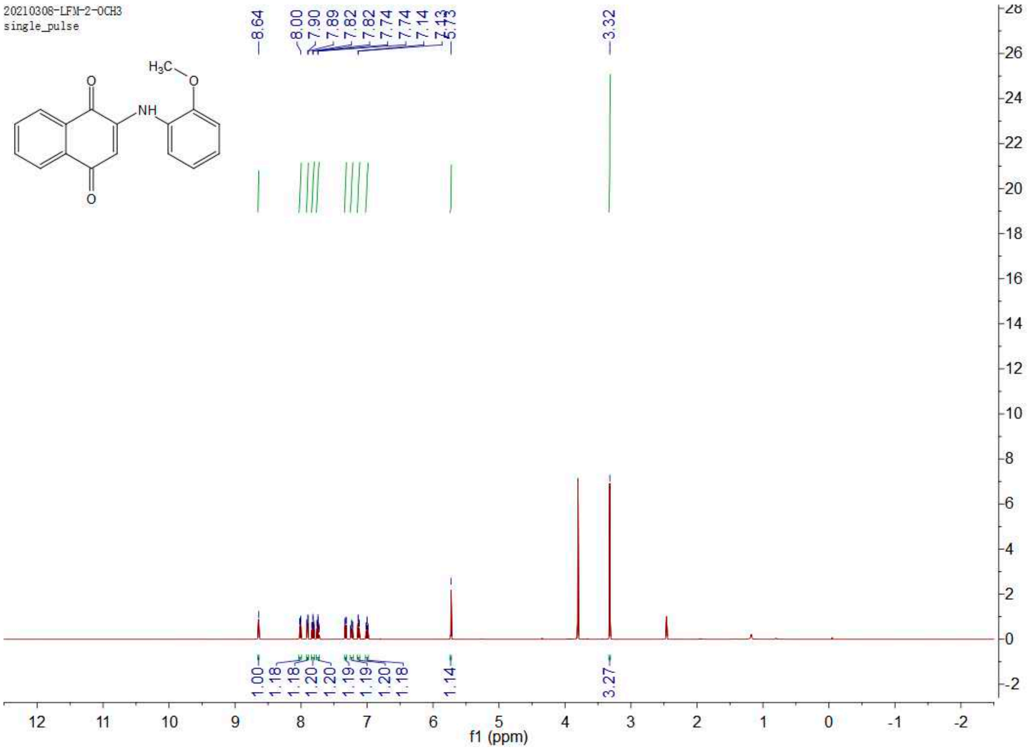

Fig S55. The ^{1}H NMR Spectrum of compound **5q**

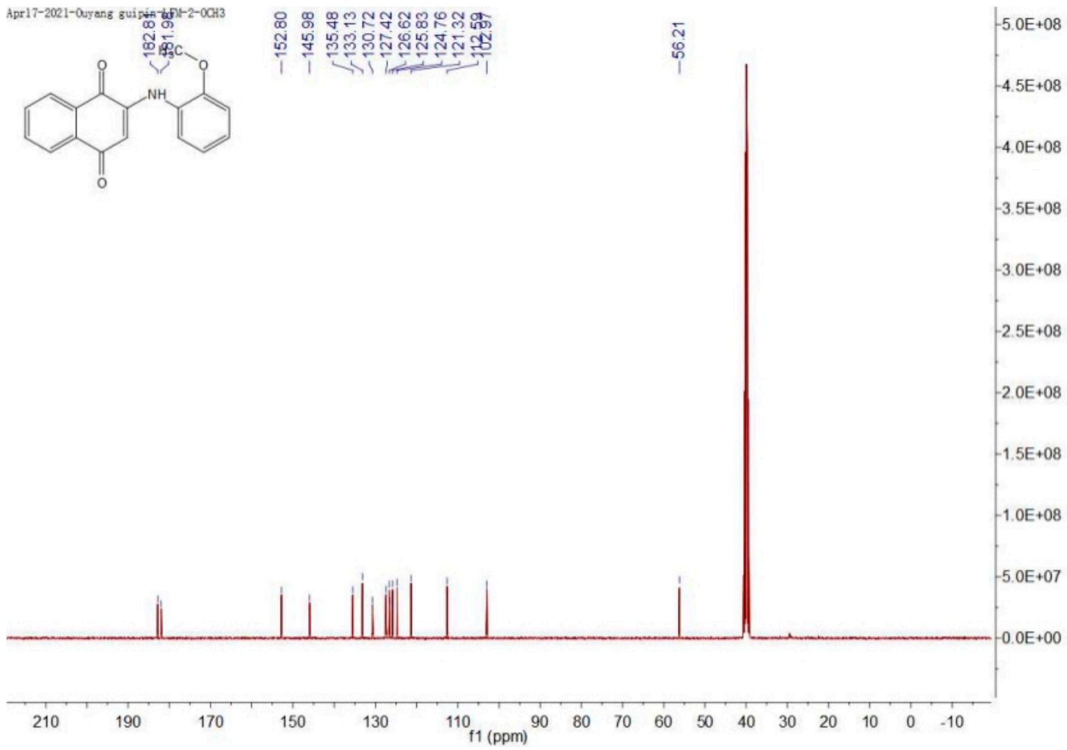

Fig S56. The ^{13}C NMR Spectrum of compound **5q**

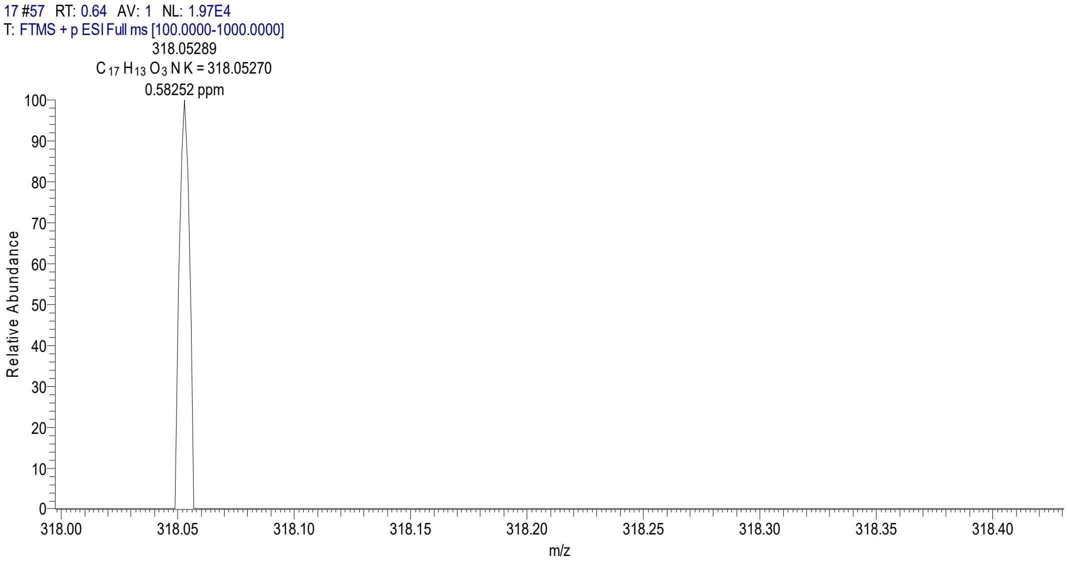

Fig S57. The HRMS spectrum of compound **5q**

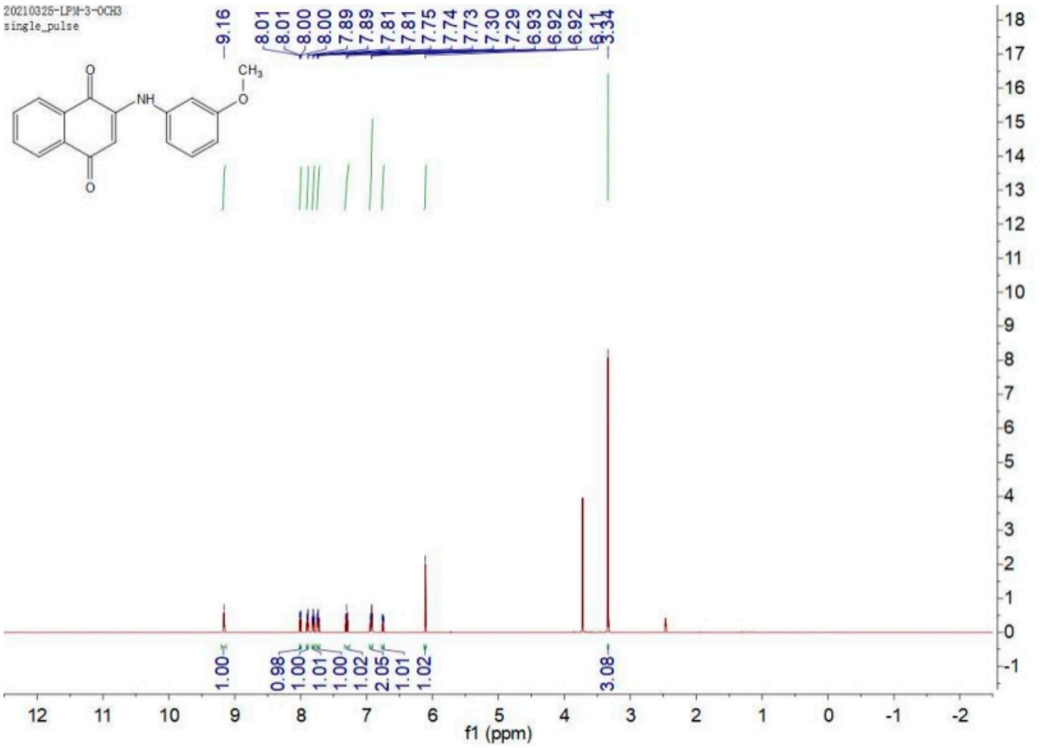

Fig S58. The ^{1}H NMR Spectrum of compound **5r**

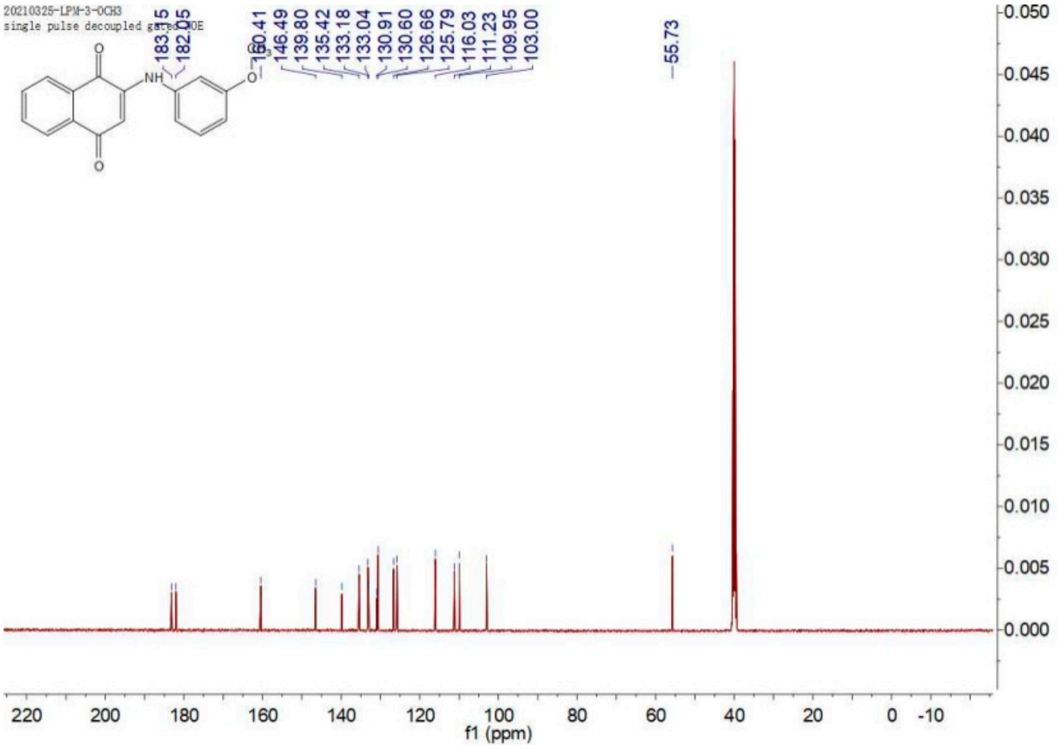

Fig S59. The ^{13}C NMR Spectrum of compound **5r**

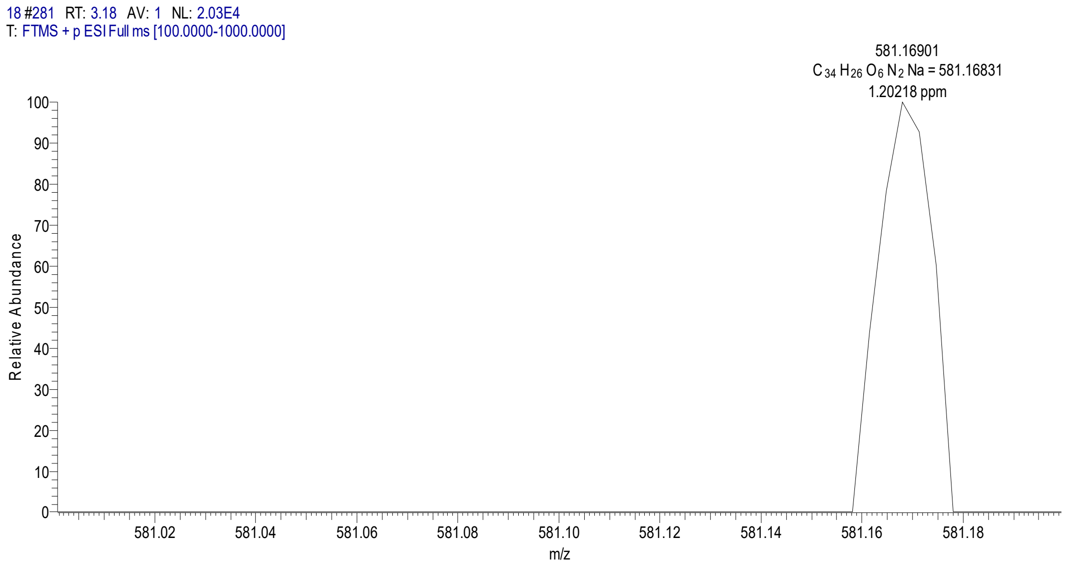

Fig S60. The HRMS spectrum of compound **5r**

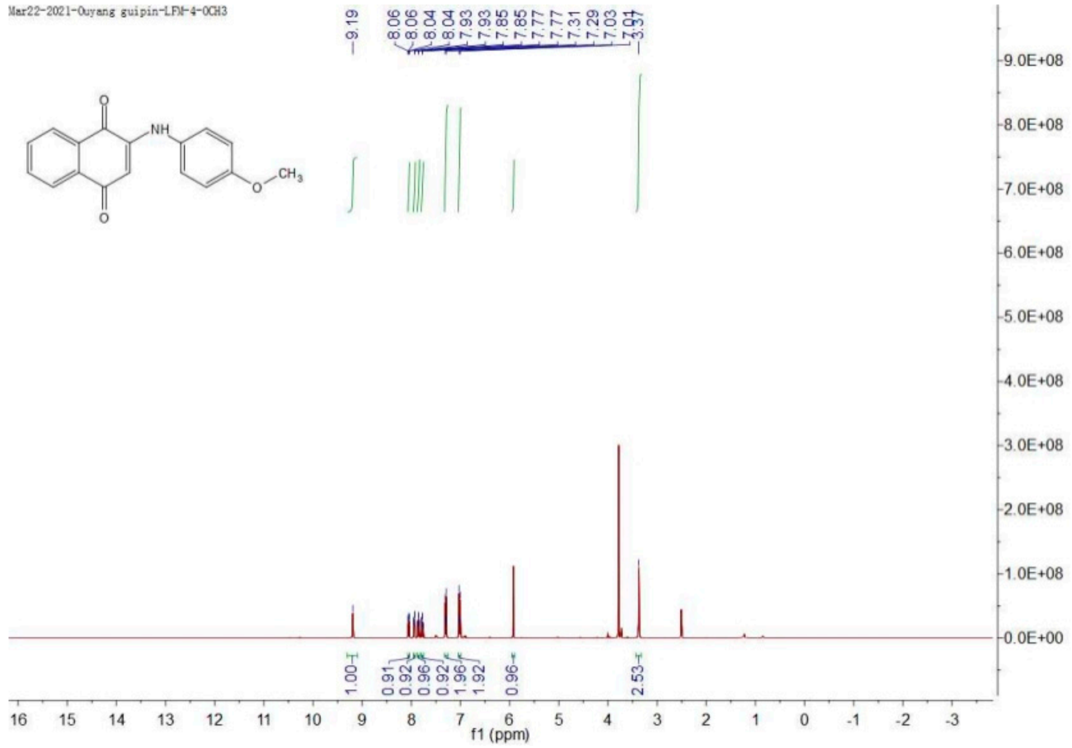

Fig S61. The ^{1}H NMR Spectrum of compound **5s**

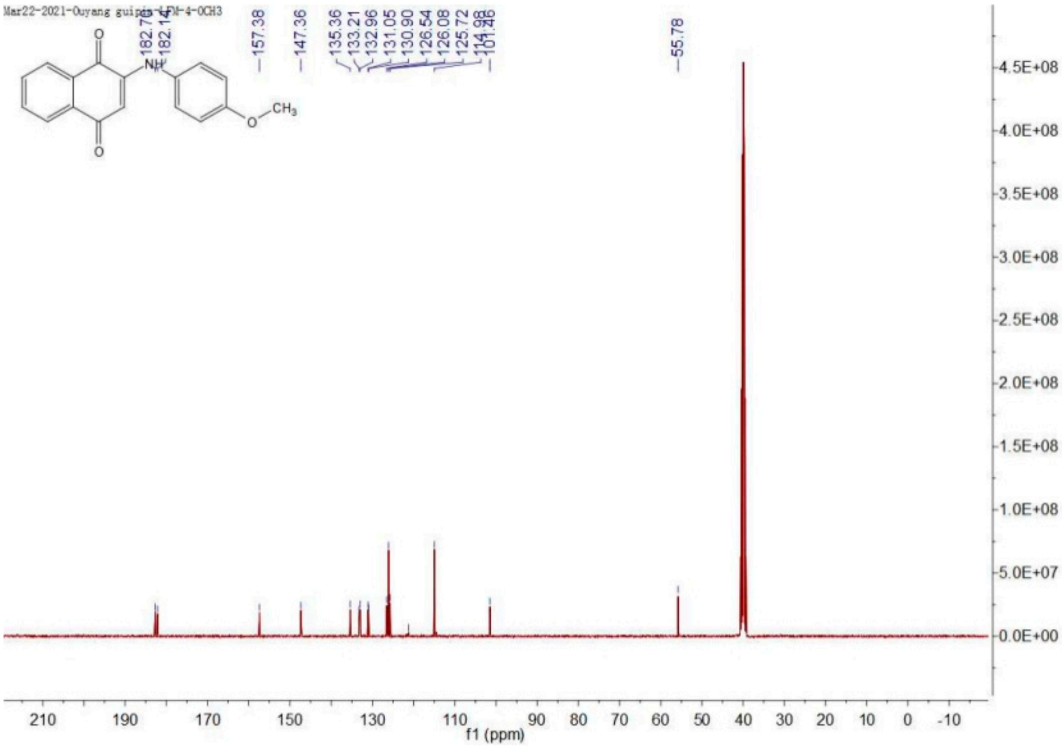

Fig S62. The ^{13}C NMR Spectrum of compound **5s**

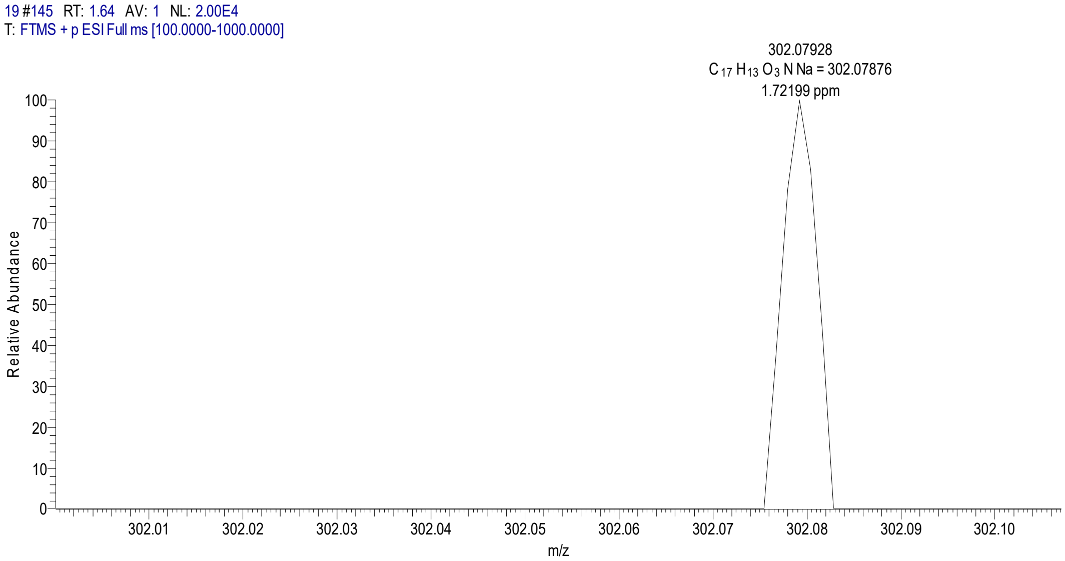

Fig S63. The HRMS spectrum of compound **5s**

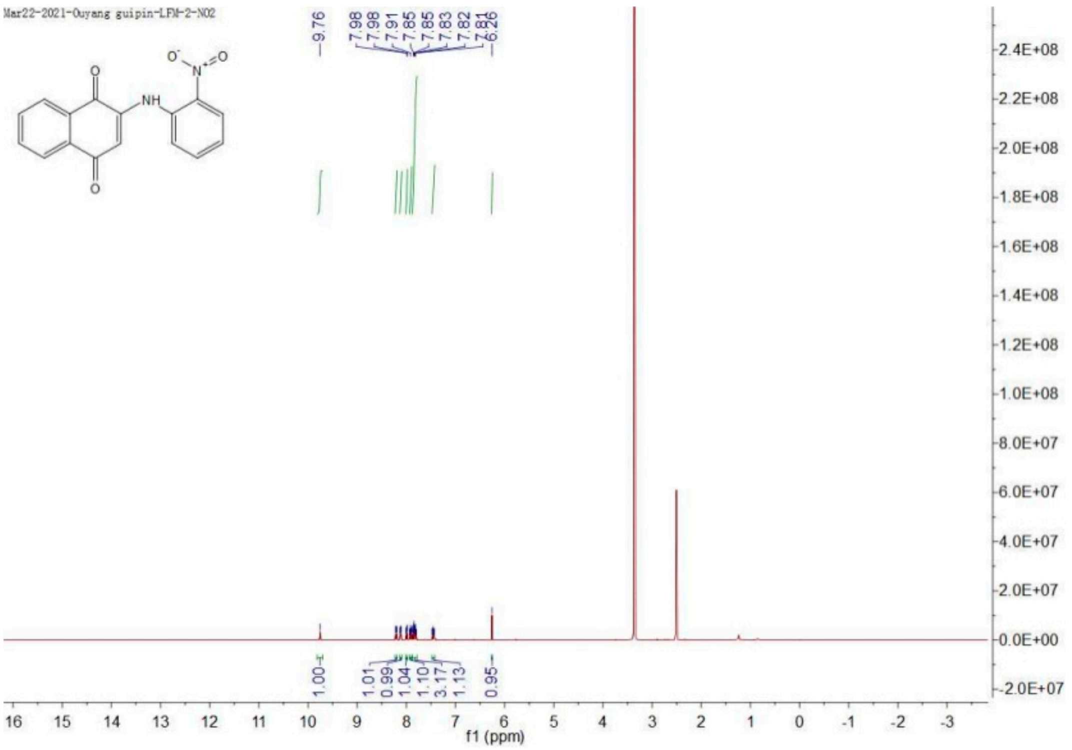

Fig S64. The ^{1}H NMR Spectrum of compound **5t**

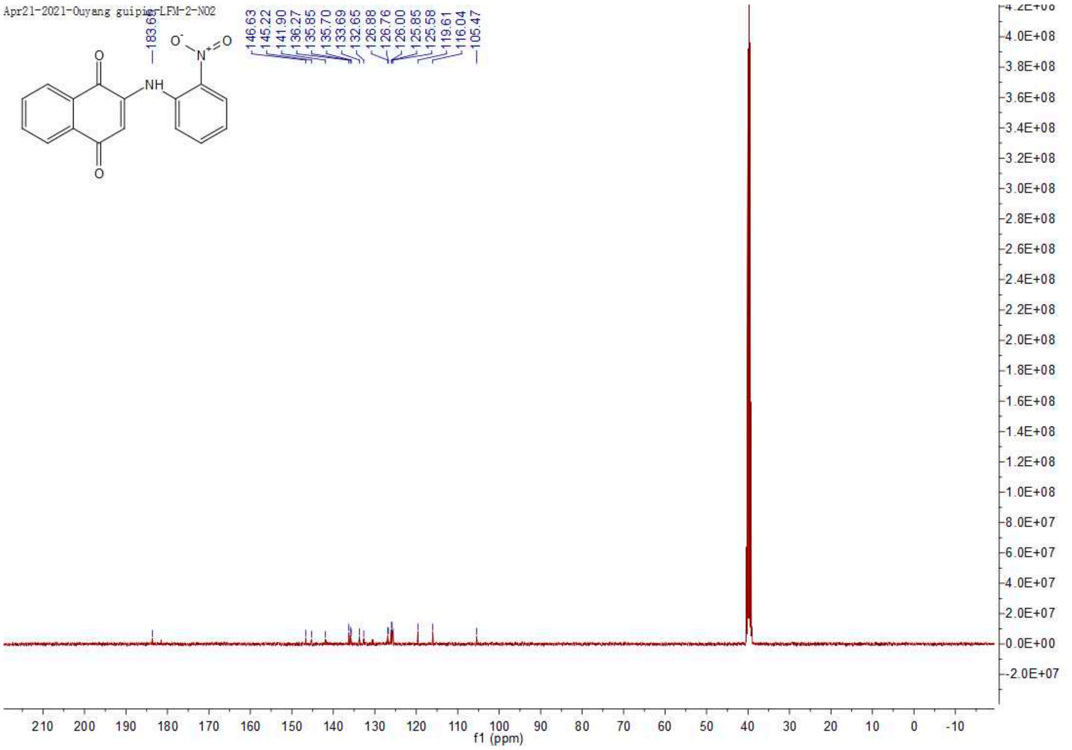

Fig S65. The ^{13}C NMR Spectrum of compound **5t**

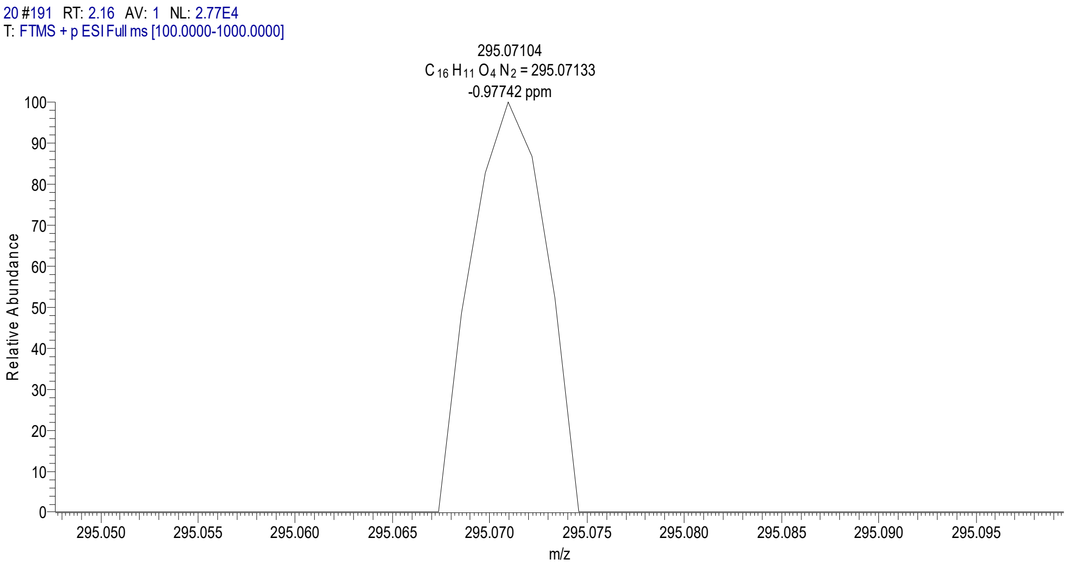

Fig S66. The HRMS spectrum of compound **5t**

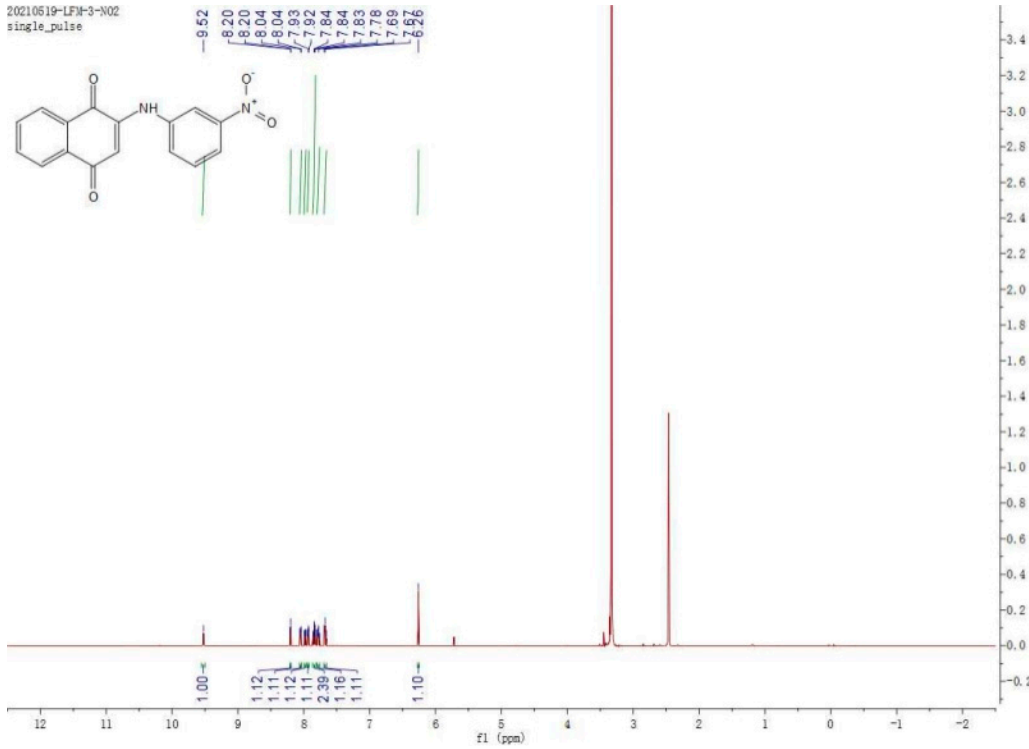

Fig S67. The ^{1}H NMR Spectrum of compound **5u**

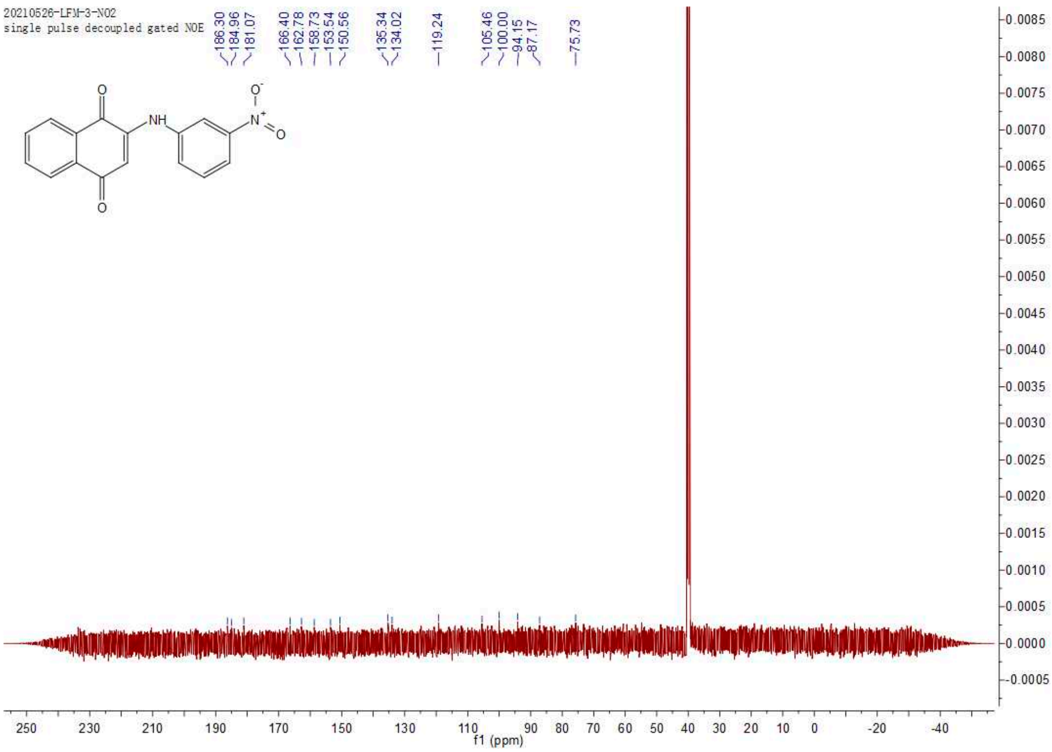

Fig S68. The ^{13}C NMR Spectrum of compound **5u**

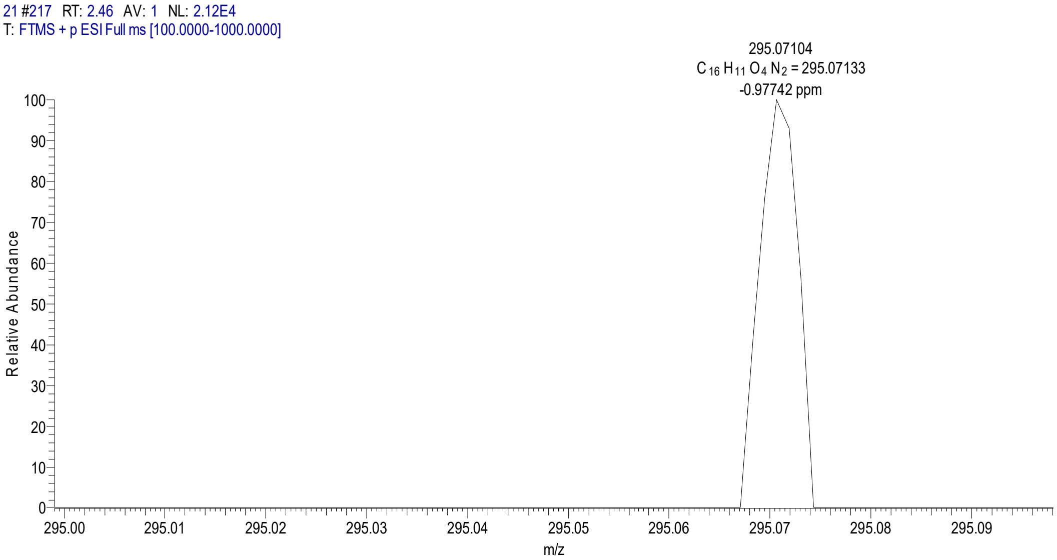

Fig S69. The HRMS spectrum of compound **5u**

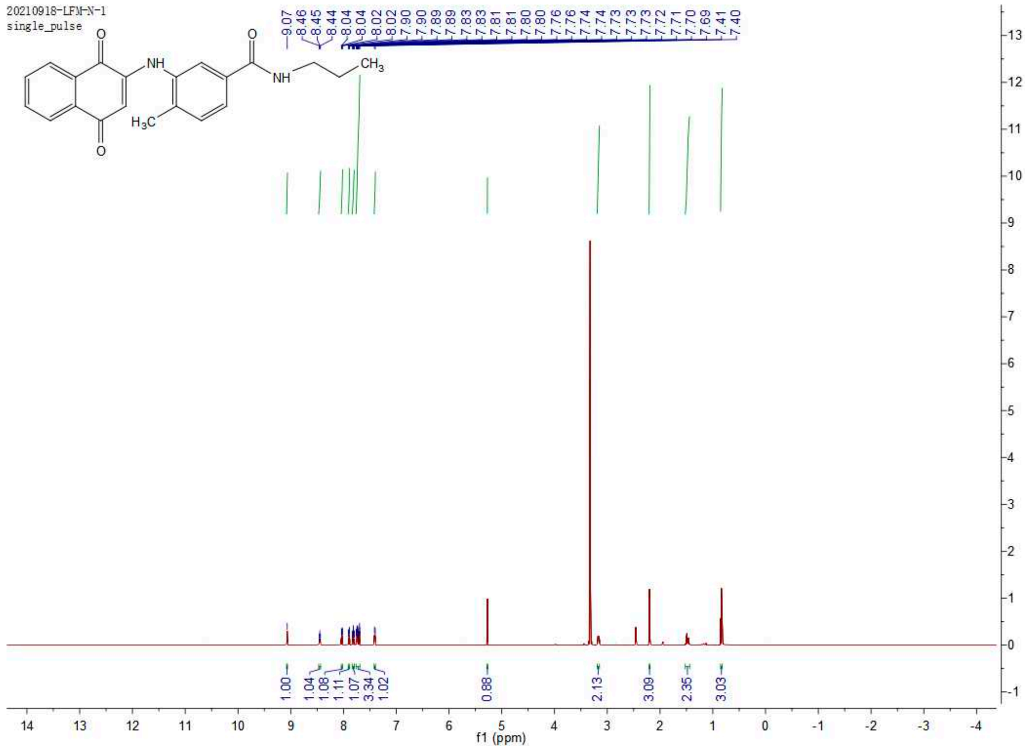

Fig S70. The ^{1}H NMR Spectrum of compound **9a**

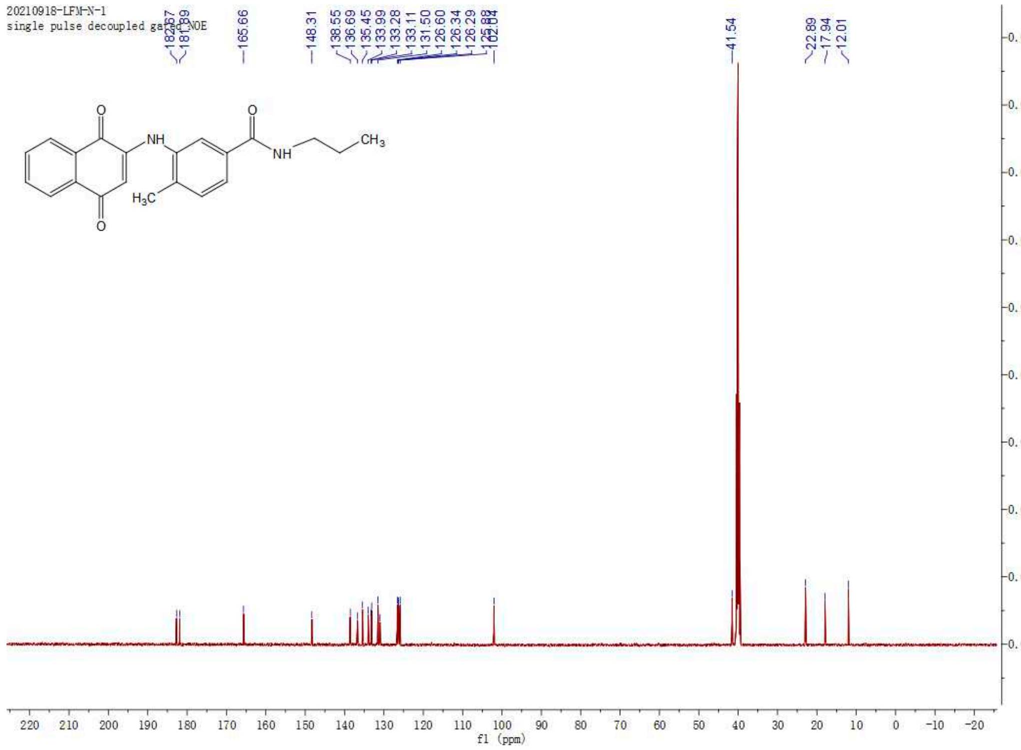

Fig S71. The ^{13}C NMR Spectrum of compound **9a**

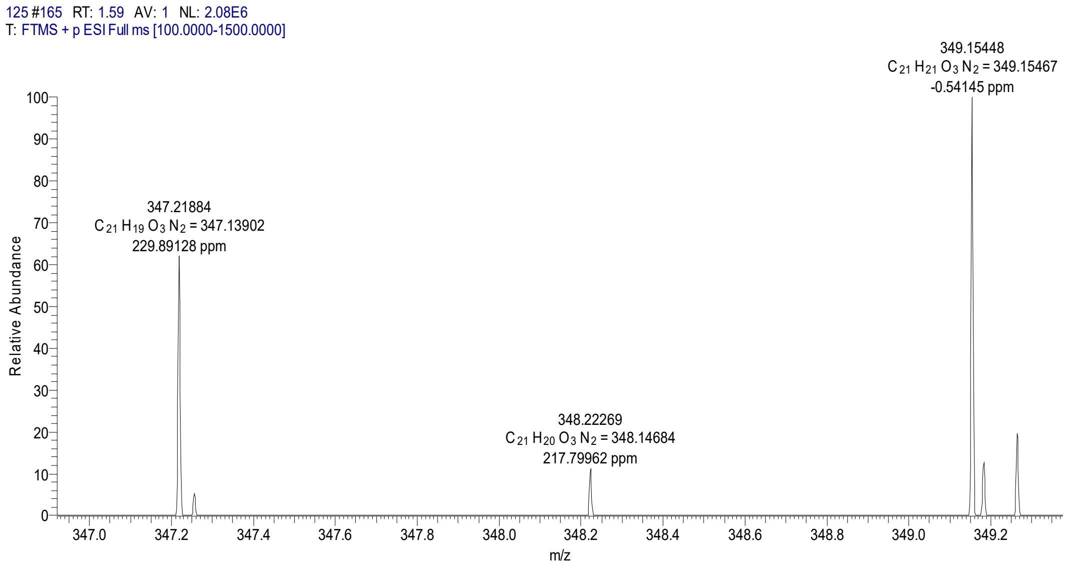

Fig S72. The HRMS spectrum of compound **9a**

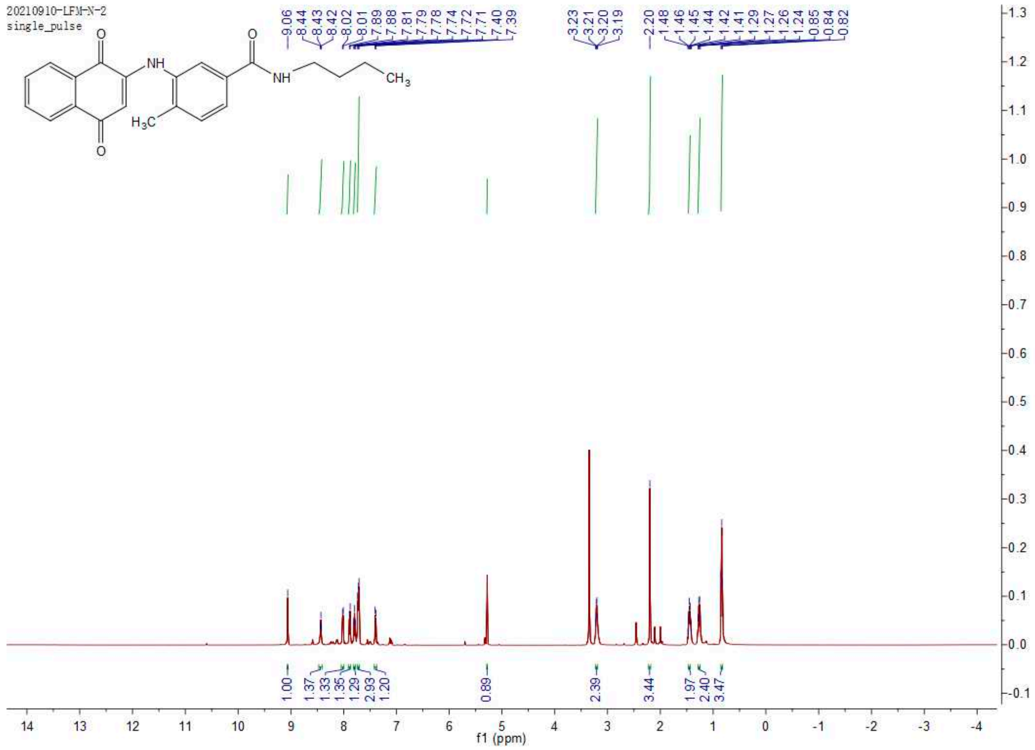

Fig S73. The ^{1}H NMR Spectrum of compound **9b**

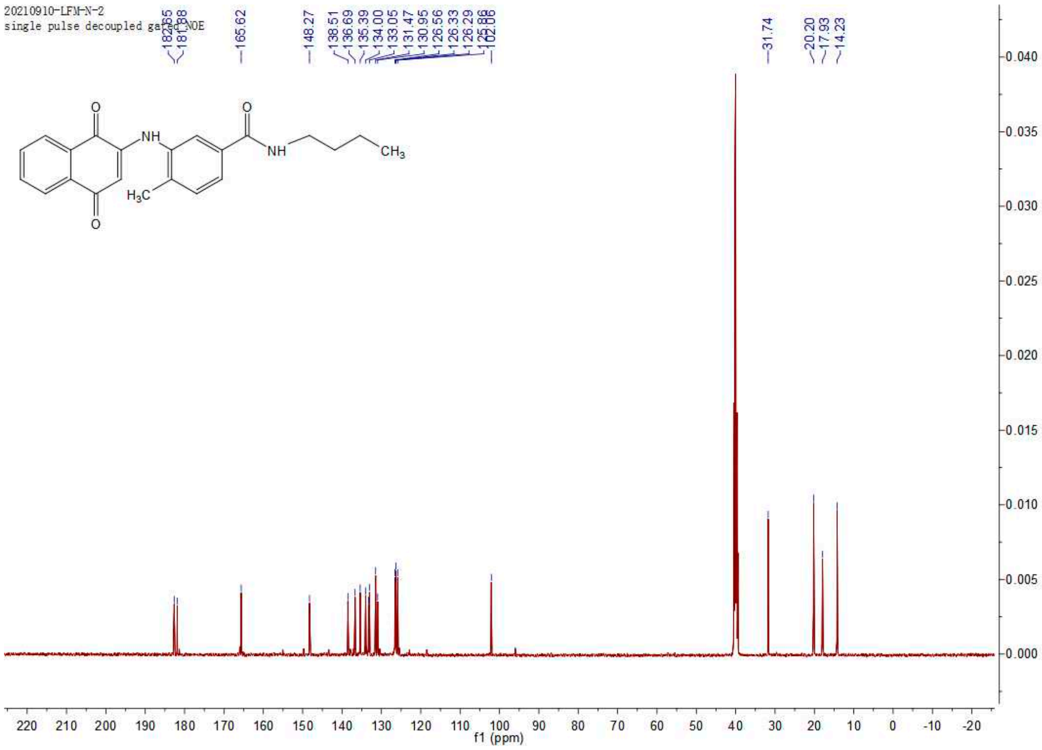

Fig S74. The ^{13}C NMR Spectrum of compound **9b**

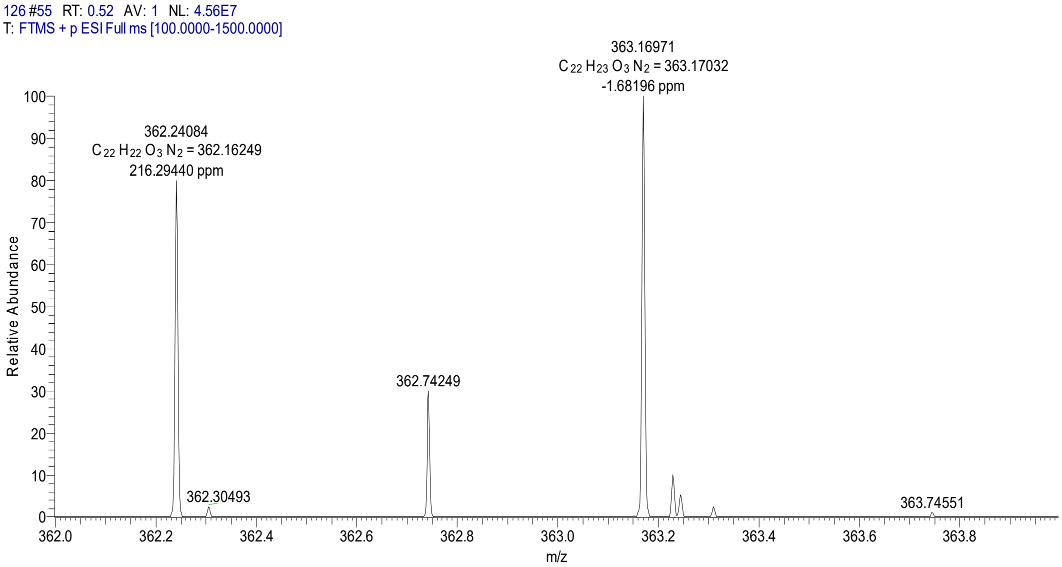

Fig S75. The HRMS spectrum of compound **9b**

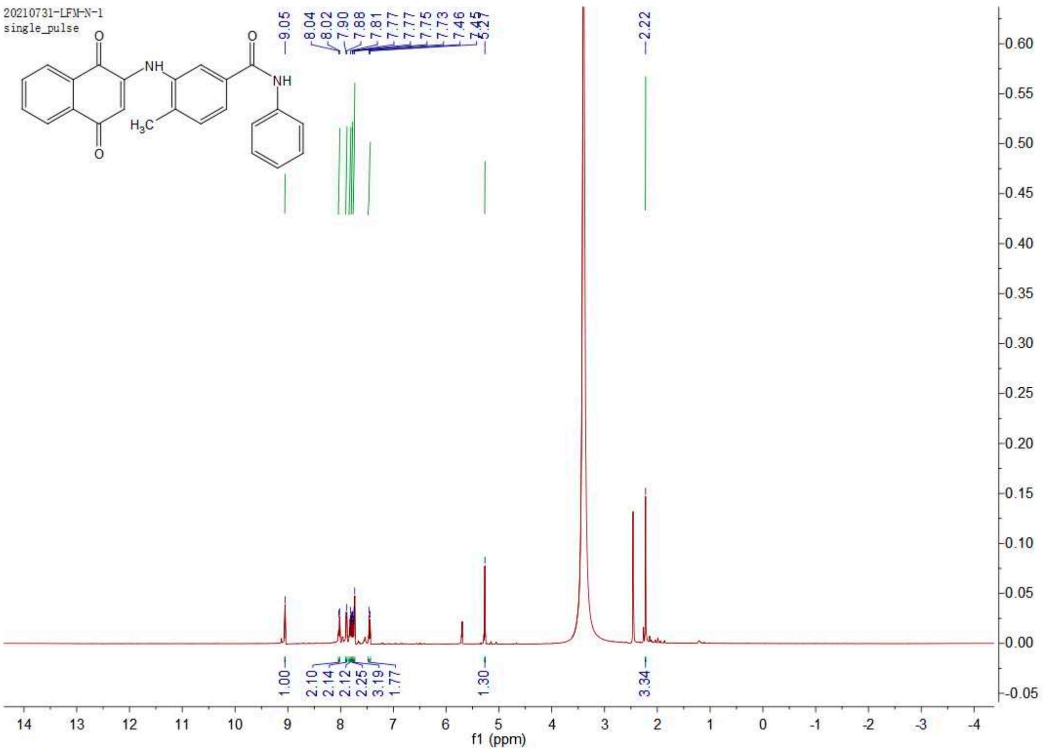

Fig S76. The ^{1}H NMR Spectrum of compound **9c**

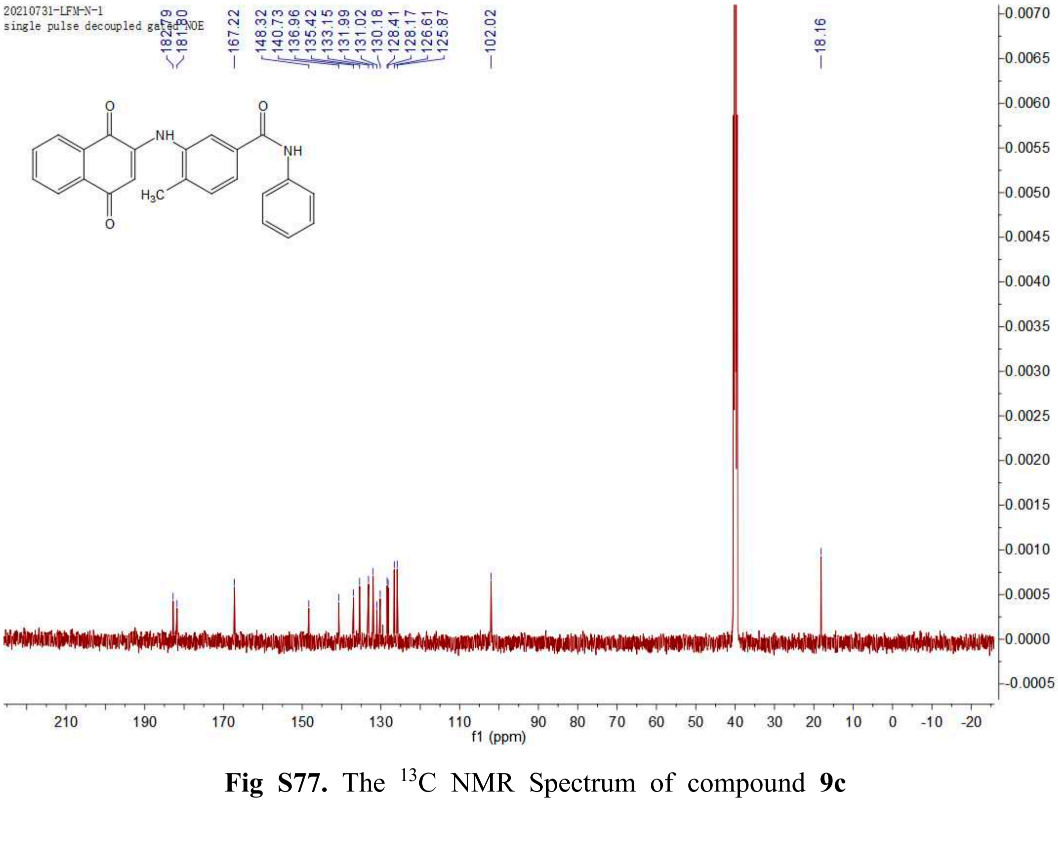

Fig S77. The ^{13}C NMR Spectrum of compound **9c**

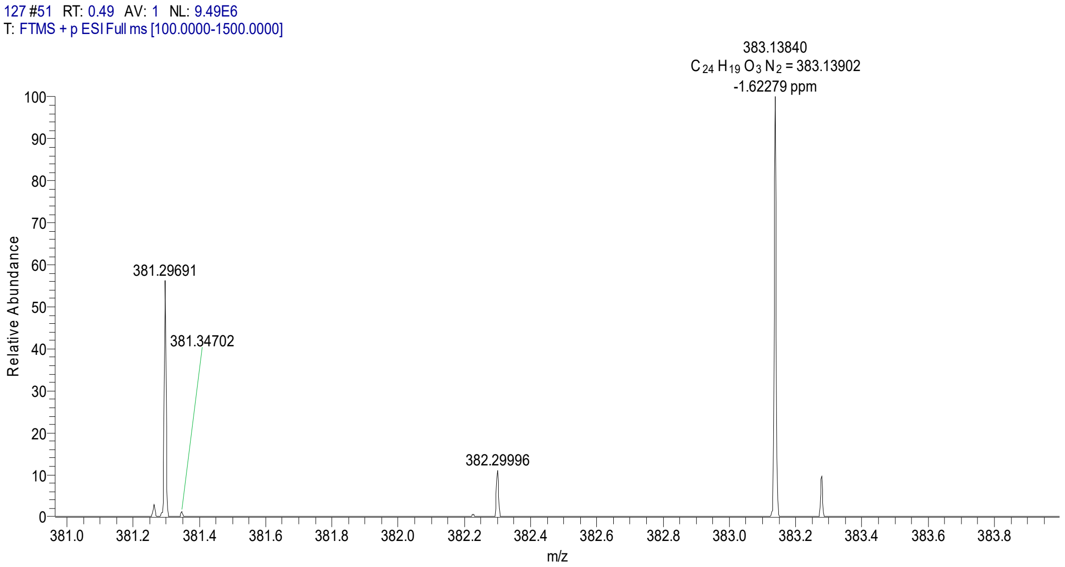

Fig S78. The HRMS spectrum of compound **9c**

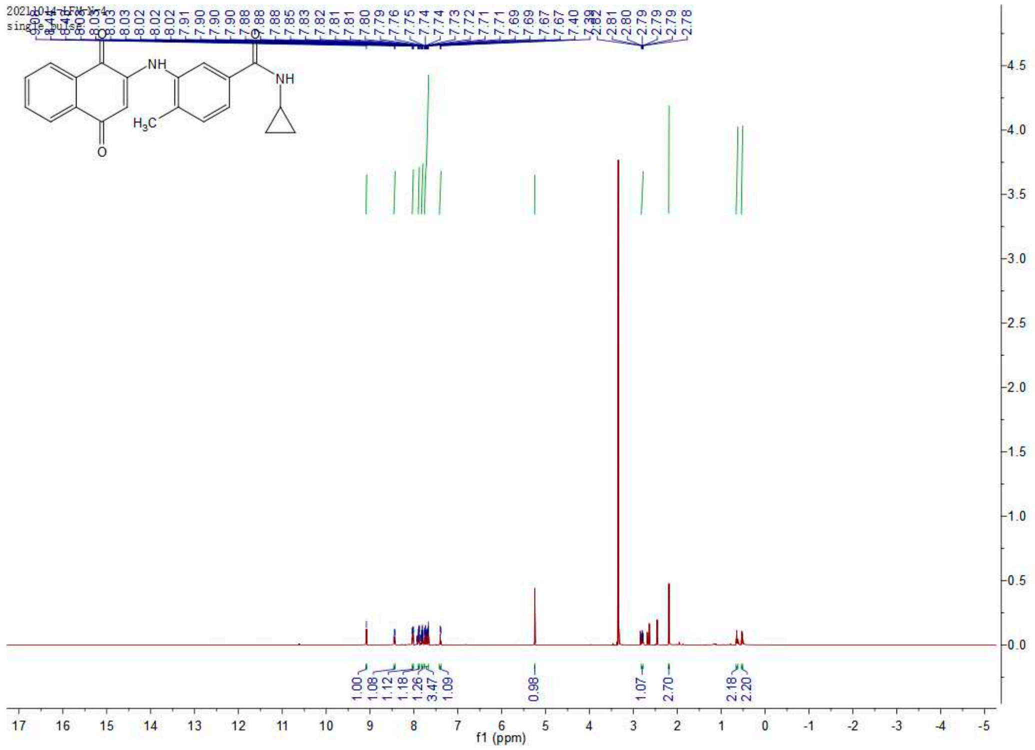

Fig S79. The ^{1}H NMR Spectrum of compound **9d**

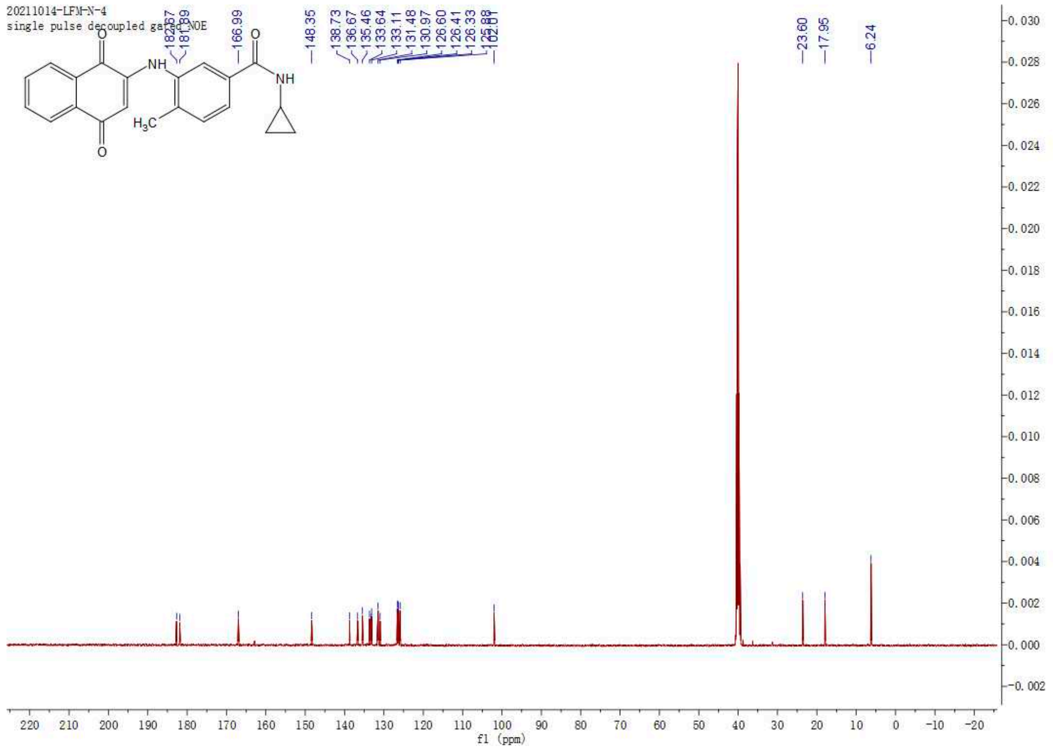

Fig S80. The ^{13}C NMR Spectrum of compound **9d**

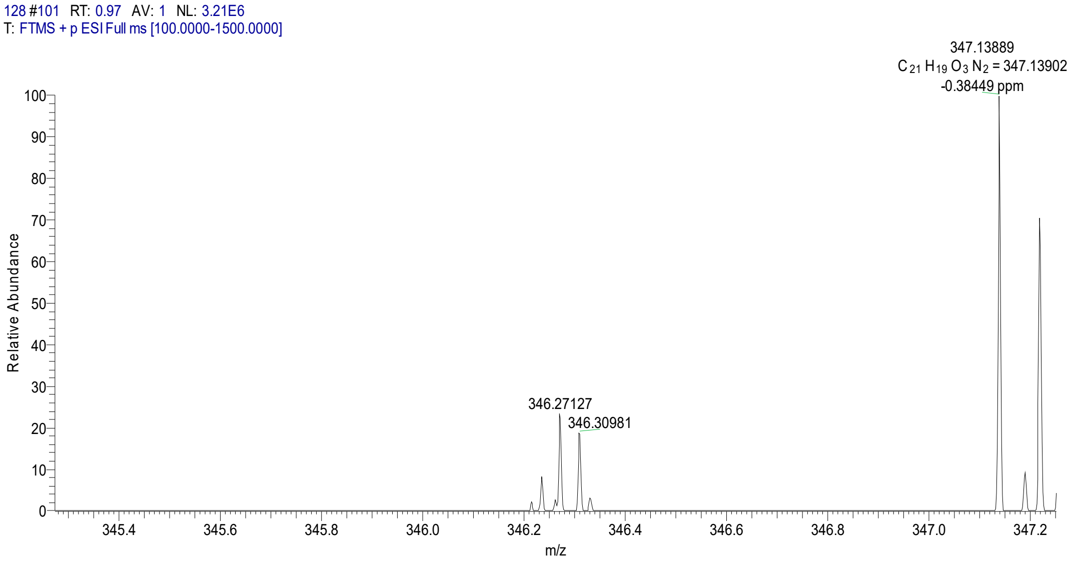

Fig S81. The HRMS spectrum of compound **9d**

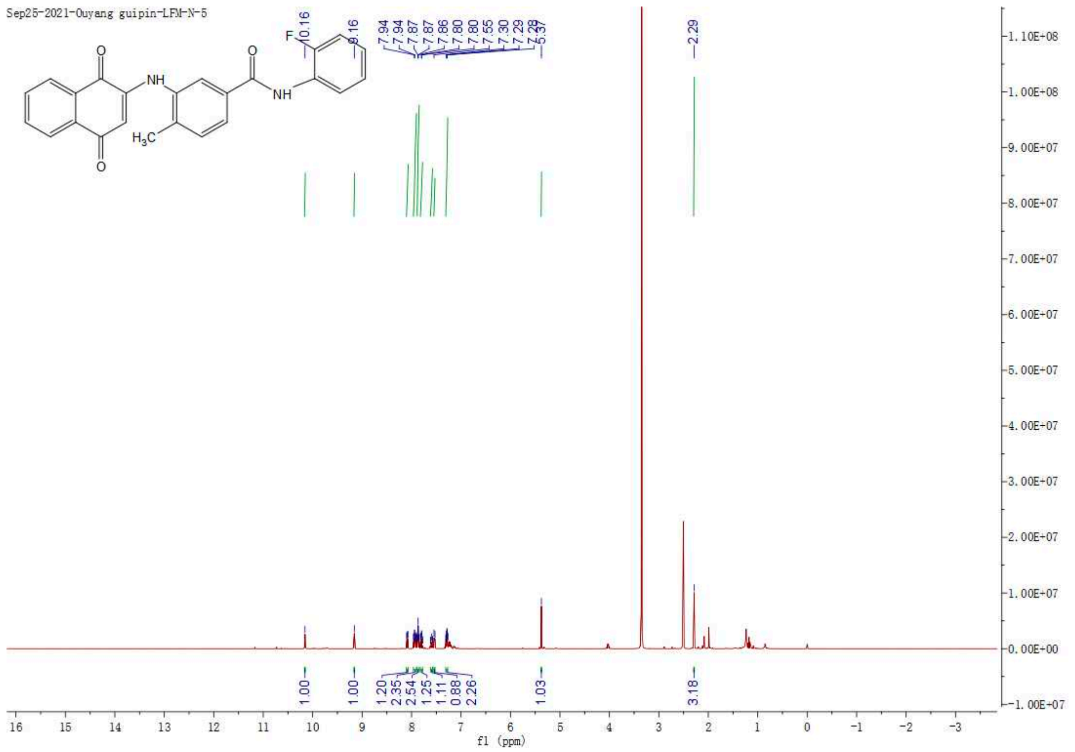

Fig S82. The ^{1}H NMR Spectrum of compound **9e**

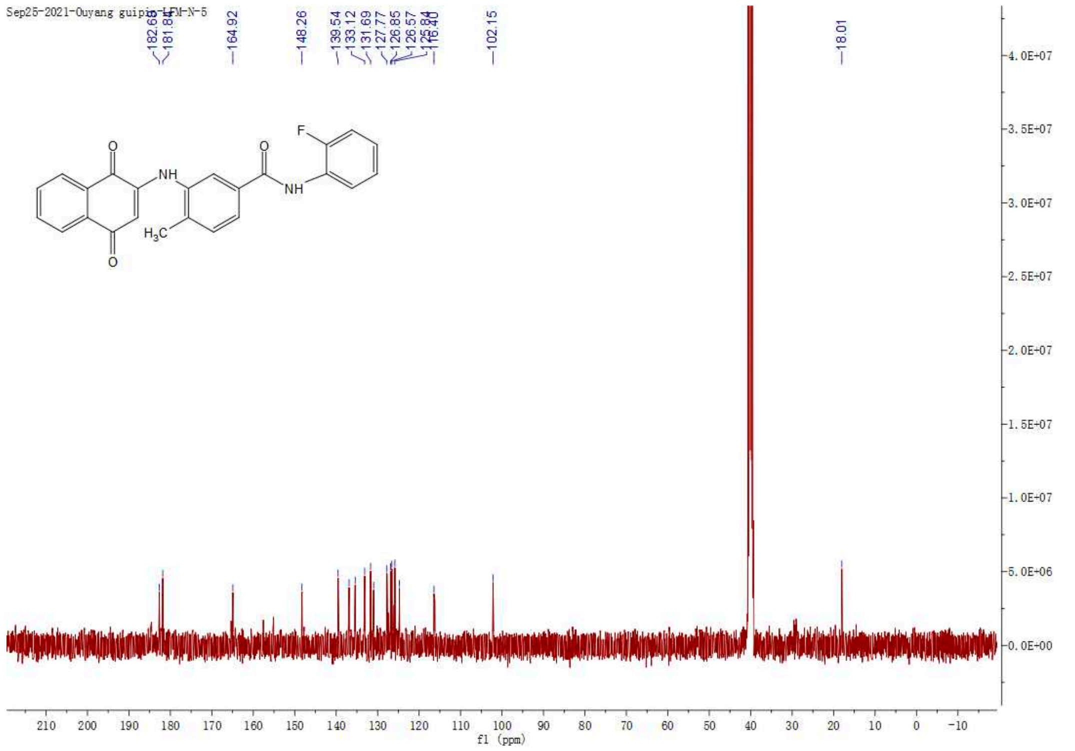

Fig S83. The ^{13}C NMR Spectrum of compound **9e**

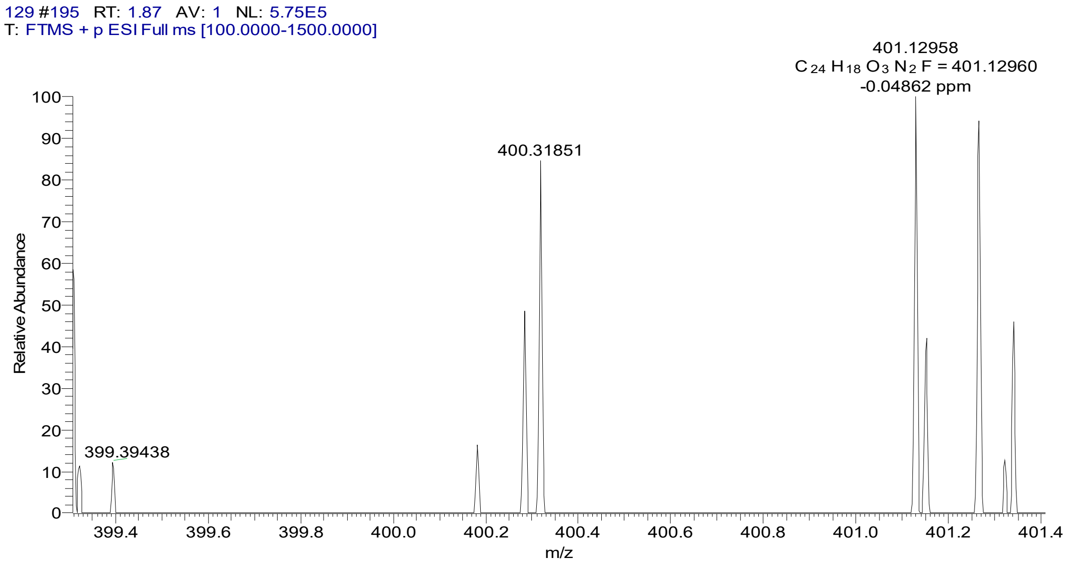

Fig S84. The HRMS spectrum of compound **9e**

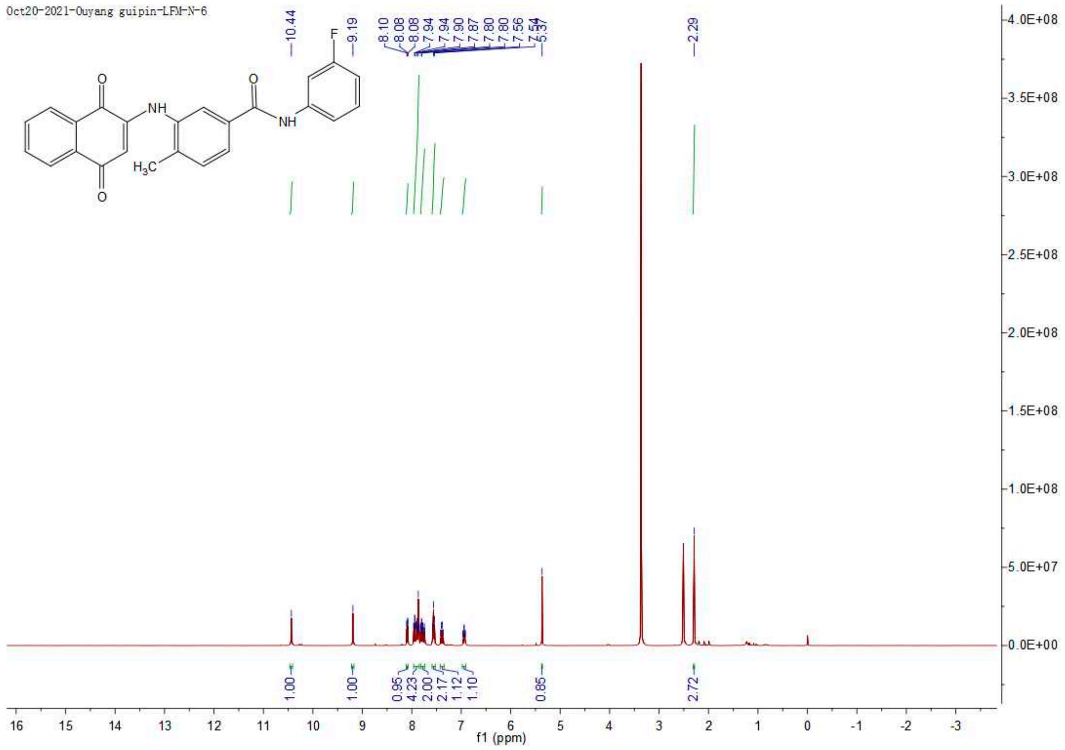

Fig S85. The ^{1}H NMR Spectrum of compound **9f**

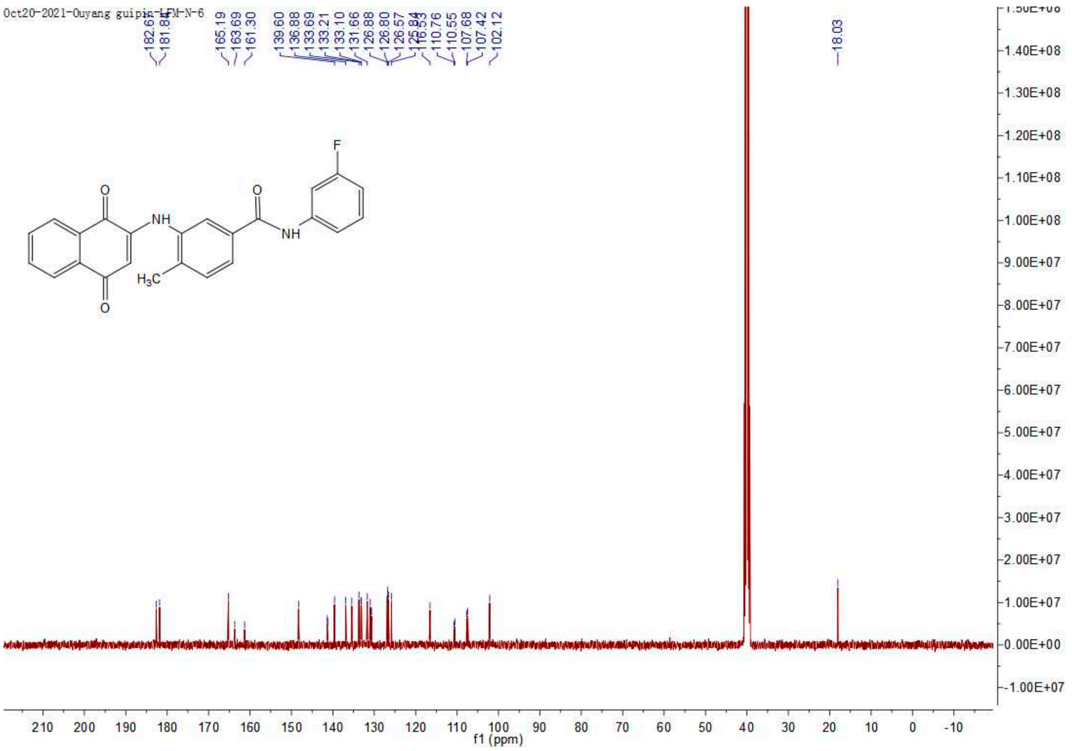

Fig S86. The ^{13}C NMR Spectrum of compound **9f**

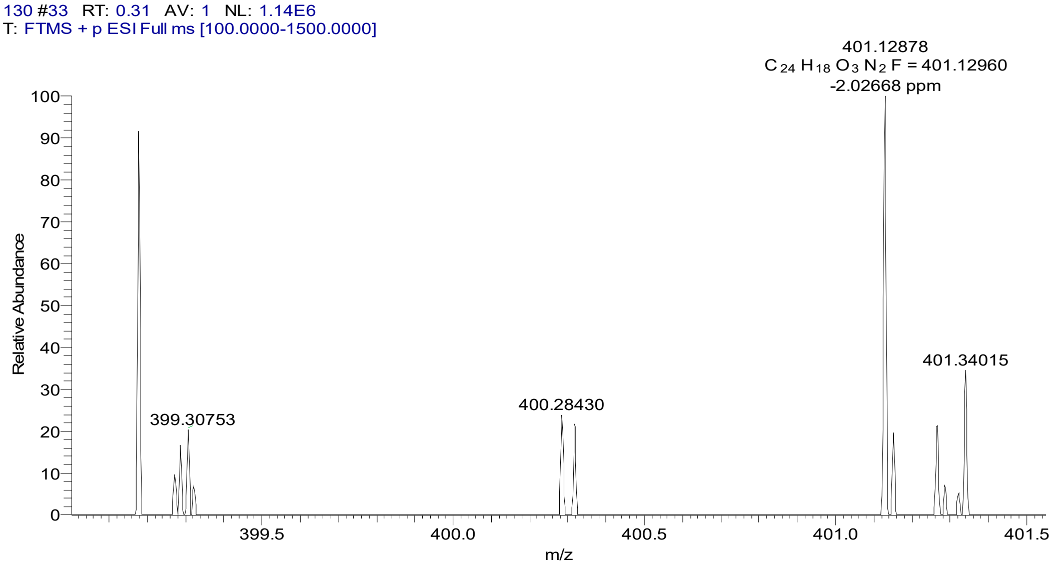

Fig S87. The HRMS Spectrum of compound **9f**

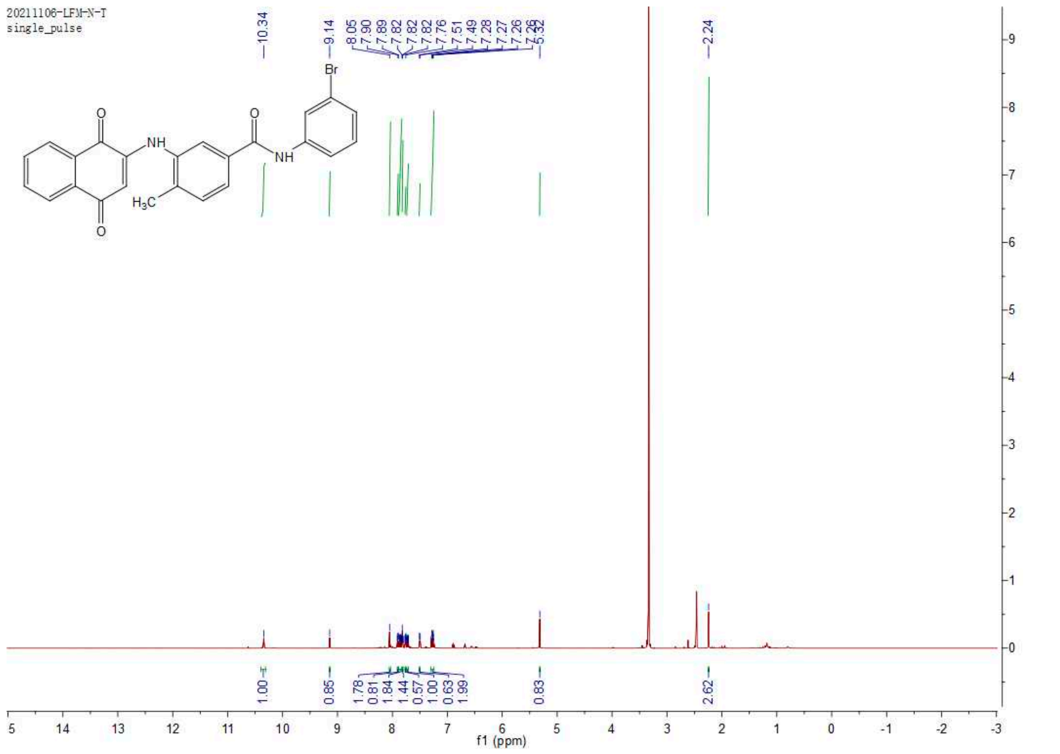

Fig S88. The ^{1}H NMR Spectrum of compound **9g**

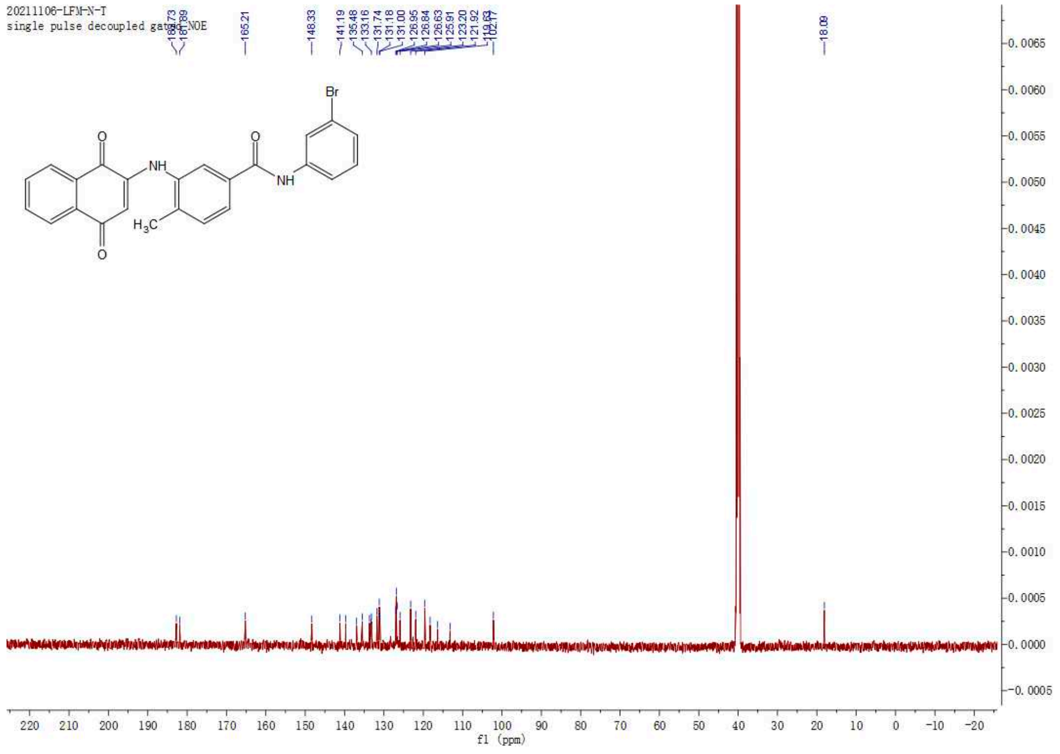

Fig S89. The ^{13}C NMR Spectrum of compound **9g**

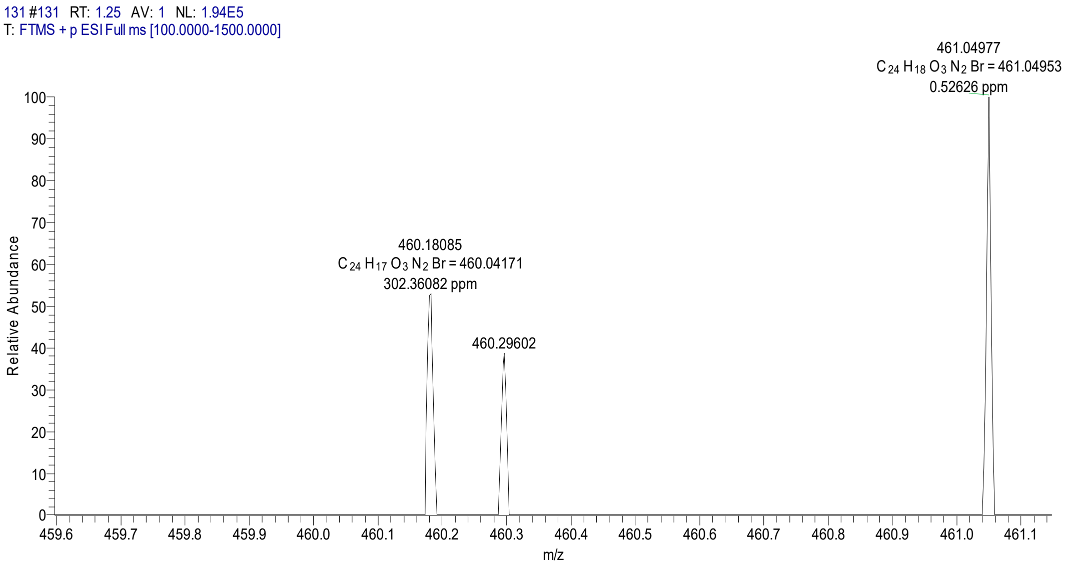

Fig S90. The HRMS Spectrum of compound **9g**

8. Crystal raw data

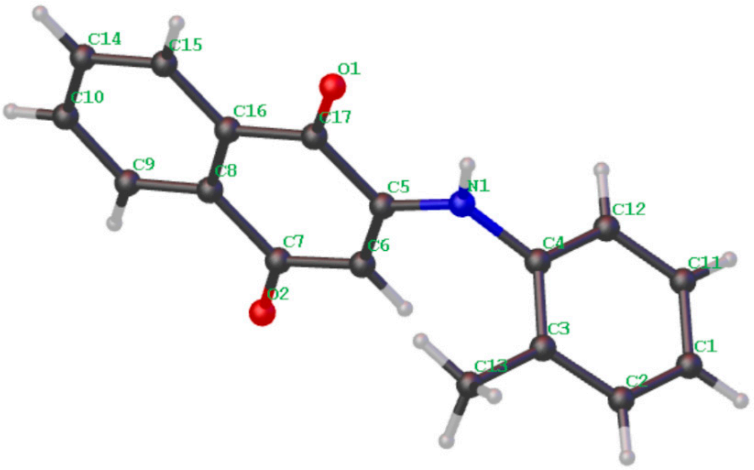

Fig S91. crystal structure of compound **5a**

Table S1 Crystal data of compound **5a**

Empirical formula	$C_{17}H_{13}NO_2$
Formula weight	262.29
Temperature/K	273.15
Crystal system	orthorhombic
Space group	$P2_12_12_1$
a/Å	7.3985(3)

b/Å	12.1825(4)
c/Å	15.1213(5)
α/°	90
β/°	90
γ/°	90
Volume/Å^3	1362.92(8)
Z	4
ρ_{calc}g/cm^3	1.278
μ/mm^{-1}	0.68
F (000)	549.8
Radiation	Cu Kα (λ = 1.54178)
2Θ range for data collection/°	9.32 to 133.14
Index ranges	$-8 \leqslant h \leqslant 8, -14 \leqslant k \leqslant 14, -11 \leqslant l \leqslant 17$
Reflections collected	7003
Independent reflections	2353 [R_{int} = 0.0514, R_{sigma} = 0.0512]
Data/restraints/parameters	2353/0/182
Goodness-of-fit on F^2	0.752
Final R indexes [I>=2σ (I)]	R_1 = 0.0526, wR_2 = 0.1543
Final R indexes [all data]	R_1 = 0.0643, wR_2 = 0.1819
Largest diff. peak/hole / e Å^{-3}	0.17/-0.17
Flack parameter	-0.2(5)

9. Reference

1. Li, K.; Yang, K.; Zheng, L.; Li, Y.; Wang, Q.; Lin, R.; He, D. Anti-acute myeloid leukemia activity of 2-chloro-3-alkyl-1,4-naphthoquinone derivatives through inducing mtDNA damage and GSH depletion. *Bioorganic & Medicinal Chemistry*, **2018**, 26(14), 4191–4200.
2. Lisboa, C. da S.; Santos, V. G.; Vaz, B. G.; de Lucas, N. C.; Eberlin, M. N.; Garden, S. J. C−H Functionalization of 1,4-Naphthoquinone by Oxidative Coupling with Anilines in the Presence of a Catalytic Quantity of Copper(II) Acetate. *The Journal of Organic Chemistry*, **2011**, 76(13), 5264–5273.
